# Discovery of Fusadapamides, Accessory Chromosome-Associated Metabolites Incorporating l‑2,3-Diaminopropionic Acid in *Fusarium poae*

**DOI:** 10.1021/acs.jnatprod.5c01104

**Published:** 2025-12-10

**Authors:** Thomas E. Witte, Linda J. Harris, Luke Albert Paquette, Anne Hermans, Amanda Sproule, Anne Johnston, Jason Ma, Michael G. Darnowski, Whynn Bosnich, Danielle Schneiderman, Xiben Wang, Benjamin A. G. Beavington, Izhar U. H. Khan, Christopher N. Boddy, David P. Overy

**Affiliations:** † 6337 Agriculture and Agri-Food Canada, Ottawa Research and Development Centre, Ottawa K1A 0Z2, Canada; ‡ Department of Chemistry and Biomolecular Sciences, 6363 University of Ottawa, Ottawa K1N 6N5, Canada; § 6337 Agriculture and Agri-Food Canada, Morden Research and Development Centre, Morden R6M 1Y5, Canada

## Abstract

Genome mining of fungal plant pathogens has uncovered biosynthetic gene clusters encoded on lineage-specific accessory chromosomes, revealing untapped potential for novel natural product discovery by metabolomic assessment of fungal populations. In *Fusarium poae*, a species contributing to *Fusarium* head blight on cereals, whole-genome sequencing and comparative metabolomics identified an accessory chromosome-associated biosynthetic gene cluster responsible for the production of a novel family of secondary metabolites, the fusadapamides. These linear tripeptides contain l-2,3-diaminopropionic acid (l-Dap), a rare nonproteinogenic amino acid not previously reported in fungi. Biochemical and genetic analyses revealed that *F. poae* synthesizes l-Dap via an accessory chromosome-encoded two-gene module that uniquely utilizes l-alanine as a substrate, diverging from known bacterial and plant l-Dap biosynthesis pathways. While fusadapamide production appears limited within *Fusarium*, homologous l-Dap biosynthetic modules were identified across diverse ascomycetes, suggesting a broader role in fungal secondary metabolism. This study highlights the power of using untargeted metabolomics at population-scale to uncover accessory chromosome-linked biosynthetic innovations and expands our understanding of fungal natural product biosynthesis.

## Graphical abstract

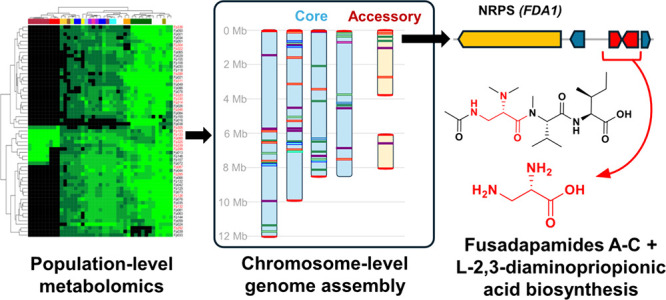

Fungal plant associates, including pathogens and endophytes, are a rich source of bioactive natural products, yet much of their biosynthetic potential remains unexplored. The use of untargeted metabolomic profiling combined with secondary metabolite biosynthetic gene cluster (BGC) mining in high quality, contiguous genomes has proven to be a powerful approach for revealing novel biosynthetic pathways in understudied species.^1,2^ *Fusarium poae* is a prolific secondary metabolite producer and a globally distributed plant pathogen commonly isolated from Fusarium Head Blight (FHB)-symptomatic wheat, barley and oats. While various trichothecenes, fusarins and beauvericin are well-known mycotoxin metabolites produced by *F. poae*, the full breadth of the secondary metabolite biosynthetic potential of *F. poae* remains largely uncharacterized.^3,4^ Recent genomic and metabolomic studies have highlighted the dynamic nature of the *F. poae* genome, particularly the presence of small, rapidly evolving ‘accessory’ chromosomes harboring transcriptionally active BGCs.^2,5^ Accessory chromosomes are known to contribute to genome plasticity in fungal plant pathogens, exhibiting frequent transposable-element mediated genome rearrangements and gene copy number variations. These dynamic genome regions may also contain lineage-specific BGCs that drive metabolic diversification and contribute to host-specific pathogenicity or other forms of niche adaptation within a population.^6,7^ The structural organization of *F. poae* accessory chromosomes remains poorly understood due to a lack of ‘closed’ or telomere-to-telomere genome assemblies. However, the identification of an accessory chromosome-associated BGC responsible for the production of cytotoxic apicidins,^2^ in addition to copy number expansion of koraiol BGCs^8^ on accessory chromosomes, suggests that accessory chromosomes are hotspots for secondary metabolism evolution.

In this study, an untargeted metabolomics approach coupled with long-read genome sequencing was applied to systematically investigate novel secondary metabolism associated with *F. poae*. By screening axenically cultured populations of Western Canadian *F. poae* strains, lineage-specific metabolite production was observed – and hypothesized to be indicative of novel biosynthetic genes encoded on accessory chromosomes. Long-read sequencing of an individual *F. poae* strain exhibiting a distinct chemical phenotype enabled the assembly of a high-quality genome, resolving the contiguous structure of two accessory chromosomes, a first for this species. A novel nonribosomal peptide synthetase (NRPS)-associated BGC on an accessory chromosome was identified and functionally characterized through gene deletion and metabolomic analysis. Structural elucidation using nuclear magnetic resonance (NMR), tandem MS/MS, and isotopic labeling revealed a previously unreported tripeptide natural product incorporating the rare nonproteinogenic amino acid l-2,3-diaminopropionic acid (l-Dap), the biosynthesis of which has not been previously characterized in fungal systems. Genetic and biochemical characterization of the key biosynthetic genes responsible for l-Dap formation in *F. poae* revealed an enzymatic pathway distinct from those previously described in bacteria and plants.^9–11^ This study demonstrates the power of untargeted metabolomics in revealing new biosynthetic pathways and biochemical space associated with fungal accessory chromosomes, expanding our understanding of fungal natural product biosynthesis.

## Results and Discussion

### Chemical Screening of *F. poae* Populations Identifies a Novel, Strain-Specific Molecular Family

Comparison of the chemical phenotypes of 59 Canadian *F. poae* strains cultured axenically on four media conditions revealed all strains expressed core phenotypes consistent with typical *F. poae-*associated metabolism, along with distinct lineage-specific mass features. All strains were isolated from barley, wheat and oat surveys and were confirmed as *F. poae* by analysis of *tef1α* gene sequence. The observed *F. poae* chemical phenotype was characterized by the production of type A- and B-trichothecenes (4,15-diacetoxyscirpenol, 4,15-diacetoxynivalenol, 15-monoacetoxyscirpenol, fusarenon-X, neosolaniol, nivalenol and scirpentriol), the cyclic depsipeptide beauvericin, cyclic lipodepsipeptides W-493 A and B, the hybrid peptide-polyketide fusarins, and the naphthoquinones aurofusarin and rubrofusarin (Figure 1A). T-2 and HT-2 toxins were not detected in any of the phenotypes, further confirming these important toxins are not produced by this species.^12^

**Figure 1:**
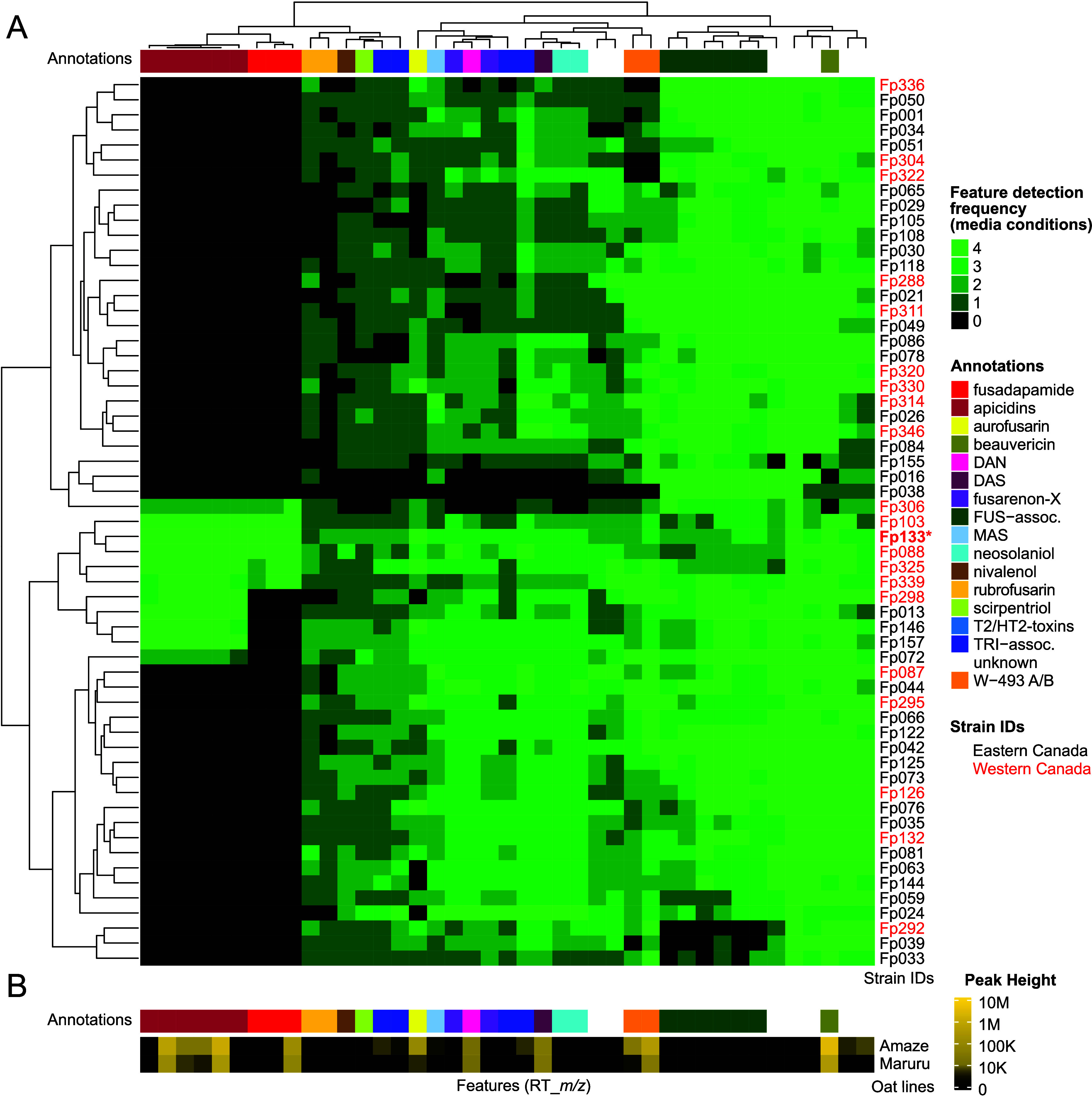
Untargeted metabolomic analysis of *F. poae* strains. (A) Simplified metabolomes of 59 strains of *Fusarium poae* isolated from Eastern Canada (black strain IDs) and Western Canada (red strain IDs). Red asterisk denotes strain *Fp*133. Heatmap values represent the number of media conditions on which a given mass feature (column) was detected for a specific strain (row), ranging from 0 (black) to 4 (bright green). Dendrograms on both axes represent hierarchical cluster analyses of detection frequencies. Strain-specific mass features clustered on the left side of the heatmap represent apicidins (dark red color bar) and fusadapamide (red color bar). Abbreviations: DAN (diacetylnivalenol); DAS (4,15-diacetoxyscirpenol); FUS-assoc. (fusarin-associated mass features); MAS (15-monoacetoxy-scirpenol); TRI-assoc. (trichothecene-associated mass features other than those verified by comparison to commercial standards or published MS/MS spectra). (B) Annotated mass feature intensities from organic solvent extracts of infected caryopses from two oat varieties, RC Amaze and Makuru, harvested 21 days after being inoculated with *Fp*133 conidia.

Several strains (*Fp*088, *Fp*103, *Fp*133, *Fp*306, *Fp*325 and *Fp*339) produced novel metabolite mass features which were without a match to our database of *Fusarium*-associated secondary metabolites, in addition to apicidins, which were previously associated with accessory chromosomes in *F. poae* (Figure 1A, bright red color bar).^2^ A mass feature showing most abundant peak intensity within the novel, lineage-specific grouping had an observed *m*/*z* of 401.2754, corresponding to a predicted chemical formula of C_19_H_37_O_5_N_4_ ([M + H]^+^). This formula was consistent with a small peptide of 3 or 4 residues. Metabolomic analysis of oat spikelets from two cultivar lines, RC Amaze and Makuru, infected with the *F. poae* strain *Fp*133, revealed the molecule associated with the observed *m*/*z* 401.27 mass feature was also produced *in planta*; however, the other mass features associated with this lineage-specific grouping were below the detection limit in infected plant samples (Figure 1B). Additionally, *in planta* production of apicidins, aurofusarin, beauvericin, trichothecenes (primarily diacetoxyscirpenol) and W-493 A and B from *Fp*133 were also observed (Figure 1B). To further investigate the lineage-specific secondary metabolite production by *Fp*133, whole genome sequencing and assembly were undertaken and scaled up fermentation was carried out to purify the corresponding metabolites (including predicted analogs) for structural elucidation.

### Fusadapamide Isolation and Structural Elucidation

Scaled up fermentation in an ^15^N-enriched medium yielded 12 mg of purified *m*/*z* 401.27 (**1**) in addition to a 2.0 mg semipure fraction containing a less abundant *m*/*z* 401.27 mass feature (**2**) that eluted at a later retention time. A third semipure fraction (2.4 mg) contained a related molecule (**3**) with a protonated *m*/*z* of 387.2604 (Figure 2A). MS^1^ [M + H]^+^ isotope ratios from the HRMS spectrum of **1** indicated approximately 91% efficiency of ^15^N uptake for a total of 57% overall ^15^N enrichment. ^1^H–^1^H COSY coupled to a 1D proton of **1** dissolved in D_6_-DMSO revealed spin systems consistent with 2,3-diaminopropionic acid (Dap), valine and isoleucine residues (Table 1) (Figure 2B, NMR spectra can be found in Supporting Information Figures S1–S12). ^15^N-HSQC and ^1^H–^1^H COSY experiments readily identified the ^15^N–H protons of Dap and Ile (δ_H_ 7.76 and 8.33 ppm, respectively). ^1^H NMR chemical shifts of all α-amino protons and the Dap β-protons were in the expected range of 3.0 to 4.0 ppm, Ile and Val β-protons were between 1.6 and 2.4 ppm, and Ile γ1-CH_2_ protons were between 1 to 1.4 ppm. Ile and Val methyl proton signals were clearly resolved in the aliphatic region of the spectrum, with Ile γ2- and δ-protons assigned to doublet and triplet peaks, respectively, and Val methyl assignments as two doublets. Three large ^1^H NMR singlets indicated the presence of four additional methyls in **1**: one methyl (δ_H_ 2.63 ppm) and one dimethyl (δ_H_ 2.37 ppm) singlet displayed chemical shifts consistent with heteroatom attachment, and another methyl singlet (δ_H_ 1.72 ppm) exhibited a chemical shift consistent with a methyl attached to a carbonyl carbon. Comparison of (^1^H,^13^C)-HSQC correlations to the expected number of carbons in **1** indicated that four carbons without attached hydrogens were present. All were attributable to amide or carboxyl carbonyl functionalities by their chemical shifts (δ_C_ 175–168 ppm). A broad singlet in ^1^H NMR (δ_H_ 8.30 ppm) was consistent with a carboxylic acid proton, indicating one carboxylic acid (δ_C_ 173.7 ppm). The three remaining carboxyl signals (δ_C_ 168.2, 170.0, 170.8 ppm) were assigned as amides, including the acetyl functional group (δ_C_ 170.0 ppm) identified by the methyl singlet (δ_H_ 1.72 ppm). Taken together, these functionalities accounted for all four degrees of unsaturation in the predicted molecular formula C_19_H_36_O_5_N_4_, suggesting **1** was a linear peptide containing Dap, Ile and Val, and an acetyl moiety, with additional features including one methylated amide and one dimethylated amine.

**Figure 2:**
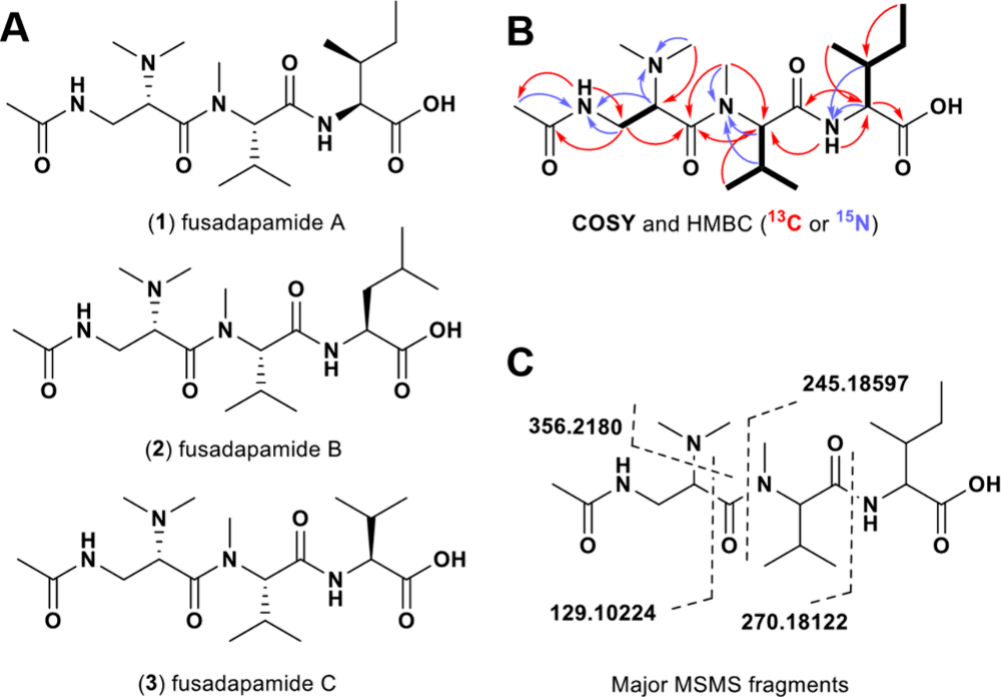
Fusadapamide structures. (A) Structure of fusadapamides A–C (**1**–**3**). (B) Fusadapamide A structure illustrating COSY correlations (bold lines), ^1^H-^13^C HMBC correlations (red arrows), and ^1^H-^15^N HMBC correlations (blue arrows). (C) Major pseudomolecular ions detected from fusadapamide A MS/MS fragmentation of mass feature *m*/*z* 401.2574 [M + H]^+^.

**Table 1:** NMR Spectra for Fusadapamide A (**1**) in Deuterated DMSO (600 MHz)

	Assignment	δ-C	δ-H (*J* in Hz)
Isoleucine			
**1**	α-CH	57.7	4.07 (dd, 8.5, 5.5)
**2**	β-CH	38.4	1.66 (m)
**3a**	γ_1_-CH_2_	25.4	1.37 (m)
**3b**		24.6	1.02 (m)
**4**	γ_2_-CH_3_	16.0	0.77 (d, 6.9)
**5**	δ-CH_3_	12.1	0.8 (t, 7.4)
**6**	COOH	173.7	
**7**	NH		8.33 (d, 8.6)
*N*-Methyl Valine			
**8**	N-CH_3_	28.4	2.63 (s)
**9**	α-CH	65.2	3.80 (d, 10.8)
**10**	β-CH	25.7	2.16 (m)
**11a**	γ_1_(CH_3_)_2_	20.3	0.89 (d, 6.4)
**11b**		18.1	0.69 (d, 6.8)
**12**	C=O	168.2	
*N*,*N*-Dimethyl Diaminopropionic Acid			
**13**	N(CH_3_)_2_	40.7	2.37 (s)
**14**	α-CH	62.2	3.70 (dd, 8.8, 4.0)
**15a**	β-CH_2_	32.9	3.59 (m)
**15b**		32.9	3.19 (m)
**16**	C=O	170.6	
**17**	AcNH	22.9	7.76 (t, 5.9)
Acetyl			
**18**	C=O	170.0	
**19**	CH_3_	22.0	1.72 (s)

Connectivity between the amino acid spin systems (Figure 2B, blue and red arrows) was supported by detailed examination of the HMBC cross-peaks correlating ^1^H NMR to both ^13^C NMR and ^15^N NMR spectra. Accordingly, HMBC correlations from the acetyl methyl protons (δ_H_ 1.72 ppm) to the Dap amide nitrogen on the β-carbon (δ_N_ 112.7 ppm), and from the dimethyl amine protons (δ_H_ 2.37 ppm) to the Dap α carbon (δ_C_ 61.2 ppm), supported the assignment of the N-terminus of the peptide as a N-α-dimethyl, N-β-acetyl Dap residue. HMBC correlations between the N–CH_3_ protons (δ_H_ 2.63 ppm) and both Dap and Val α-carbons (δ_C_ 62.2 and 65.2 ppm) revealed that Dap is connected to *N*-methyl Val. Lastly, HMBC correlations between the Ile amide N–H proton (δ_H_ 8.33 ppm) and the Val α-carbon (δ_C_ 65.2 ppm) established that Ile was connected to the *N*-methyl Val residue, and finally the presence of a carbonyl carbon (δ_C_ 173.7 ppm) with HMBC correlation to the Ile α-proton (δ_H_ 4.07 ppm) supported the Ile residue C terminus to be a carboxylic acid, making **1** a linear peptide free acid with the configuration Acetyl-Dap-Val-Ile.

MS/MS fragmentation in the HRMS system was consistent with the proposed structure of **1**, with observed a-, b- and y-ions typical of linear peptide fragmentation (Figure 2C, Figure S13). A fragment neutral loss corresponding to the mass of dimethylated amine on the Dap residue (NL = 45.05 amu) was also characteristic of the fragmentation pattern. MS/MS fragmentation analysis was also assisted by an isotopically labeled feeding experiment wherein exogenous, isotopically labeled Dap was supplemented into the growth media (discussed below).

The structures of **2** and **3** were proposed based on comparison of exact mass, NMR spectra and MS/MS fragmentation pattern to **1**. ^1^H NMR, COSY and HSQC spectra for **2** supported the presence of a β-CH_2_ and γ-CH coupled to two doublet methyls, consistent with a Leu in place of the Ile in **1** (Figures S8 to S10). ^1^H NMR and COSY spectra analysis supported the presence of a second Val residue in place of the Ile observed in **1** for **3**, which is consistent with the observed [M + H]^+^ of *m*/*z* 387.2604 (Figures S11 and S12).

**1** was named fusadapamide A, based on its discovery in *Fusarium* and the presence of a Dap residue in the molecule. Structures **2** and **3** were accordingly assigned letters B and C. The stereochemistry of *N*-methyl Val, Val, Ile and Leu residues in **1–3** were assigned as l using Marfey’s method (Figures S14–S22). Marfey’s analysis of the α-N-dimethyl Dap residues was inconclusive. Derivatization of hydrolyzed fusadapamides and an α-N-dimethyl Dap synthetic standard treated under identical conditions with both d and l N_α_-(2,4-dinitro-5-fluorophenyl)-alaninamide, generated peaks consistent with a mixture of Dap enantiomers. As enantiopure (ee >95%) Dap was used for the synthetic standard, this result suggests that Dap is epimerized upon hydrolysis (Figure S22). Epimerization of Dap has been observed in Marfey’s analysis of other DAP containing natural products.^13,14^ Dap stereochemistry was nevertheless assigned as l based on the biosynthesis (absence of an epimerization domain in Fda1) as discussed in detail below. A lack of chromatographic alignment between hydrolyzed fusadapamides and a synthetic standard of β-N-dimethyl Dap using Marfey’s method confirmed the assignment of the *N*-methyls on the α carbon for all fusadapamides.

### Fusadapamide BGC Resides on One of Two Accessory Chromosomes in *Fp*133

A combination of long-read and short-read sequencing enabled the production of a telomere-to-telomere, chromosome-level assembly for *Fp*133 (Figure 3A). The *Fp*133 genome assembly contains six chromosomes, including the four core chromosomes typical for *F. poae* karyotypes and two shorter chromosomes, Chr5 (∼3.8 Mb) and Chr6 (∼2.1 Mb)­(Figure 3B). Both Chr5 and Chr6 show indicative signatures of accessory chromosomes in *F. poae,* ^2,5^ including low gene density, high numbers of repetitive elements, and minimal areas predicted to be affected by repeat-induced point mutation (RIP) as compared to core chromosomes (Table S1). Both Chr5 and 6 exhibited fragmented or “meso” synteny compared to the accessory chromosome-associated contigs in *Fp*157.^2^ The sizes of Chr5 and Chr6 were corroborated by contour-clamped homogeneous electric field (CHEF) karyotype analysis, revealing two bands corresponding to Chr5 and Chr6 from lysed *Fp*133 protoplasts (Figure S23). Other notable features of the assembled *Fp*133 genome included two centromere-like regions of low GC content (>40 kb of <15% GC) on Chr5. Additionally, the Chr3 termini appear inverted and swapped compared to *Fp*157 and the subtelomeric region of *Fp*133 Chr3 aligns with *Fp*157 contig 2 (Figure 3B, yellow ribbons).

**Figure 3:**
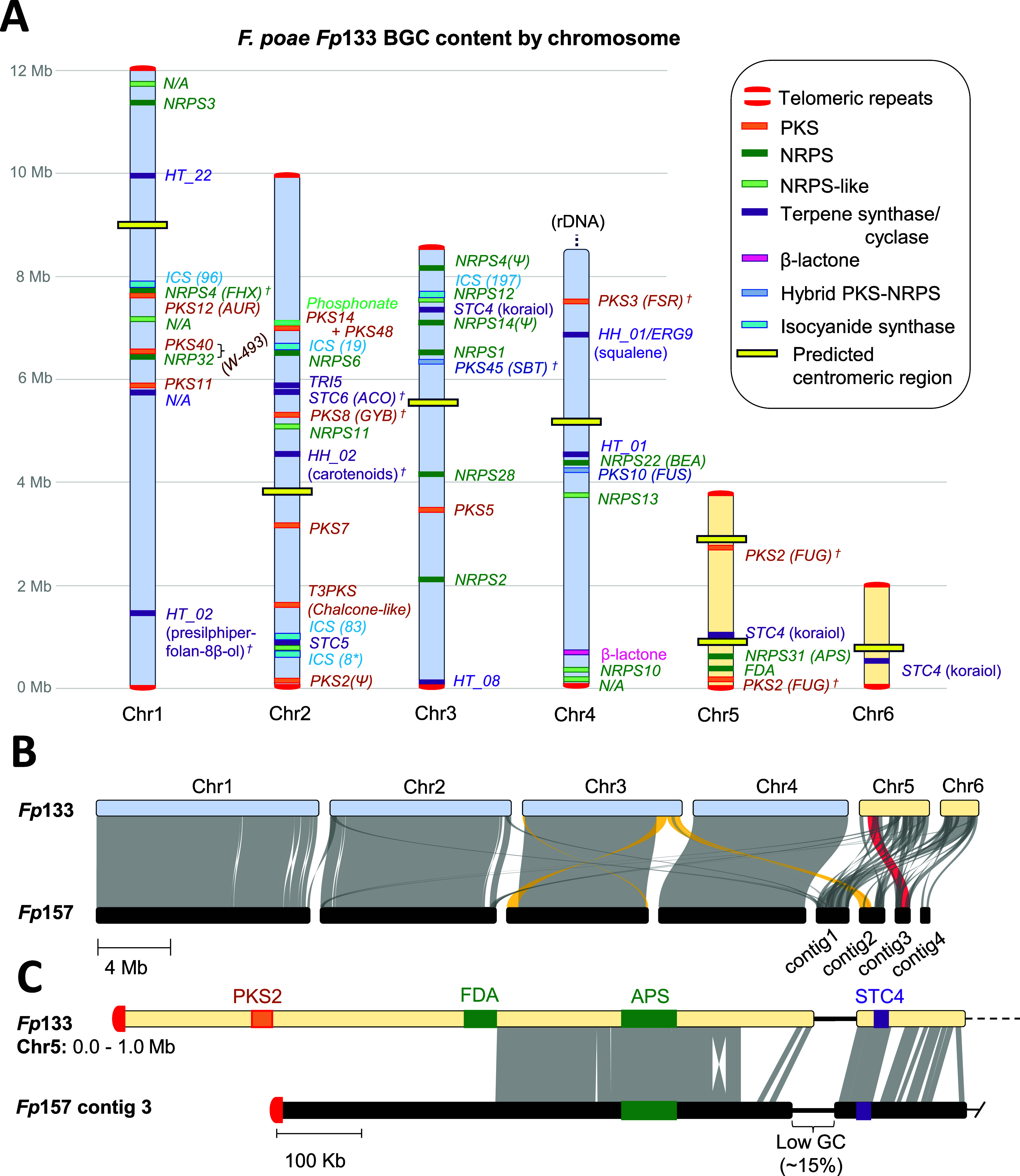
Structure of the *Fp*133 genome. (A) Visualization of the *Fp*133 genome with secondary metabolite biosynthetic gene clusters (BGCs) overlaid and annotated using broadly defined *Fusarium* nomenclature (core chromosomes colored blue; accessory chromosomes colored orange). BGCs are annotated based on similarity to known BGCs in *Fusarium*, or else, in the case of isocyanide synthase clusters (ICSs), are numbered based on the GCF family associated with the ICS core gene. The symbol † indicates the predicted BGC product has not been detected yet in *F. poae* extracts. Abbreviations: ACO (α-acorenol); APS (apicidin); AUR (aurofusarin); BEA (beauvericin); FDA (fusadapamide); FHX (fusahexin); FSR (fusarubin); FUG (fugralin); FUS (fusarin); GYB (gibepyrone); SBT (sambutoxin); NRPS (nonribosomal peptide synthetase); PKS (polyketide synthase); N/A (not annotated). (B) Whole genome synteny map comparing *Fp*133 and *Fp*157. Gray ribbons connect syntenic sequences; yellow ribbons highlight swapped subtelomeric ends of Chr3 and synteny to *Fp*157 contig 3. Red ribbon highlights syntenic sequence shared by *Fp*133 Chr5 and *Fp*157 contig 3, containing BGCs of interest. (C) Comparison of the first megabase of *Fp*133 Chr5 and *Fp*157 contig 3 (region corresponds to red ribbon in panel B).

Transcriptome-assisted gene modeling predicted a total of 14,712 gene models in the *Fp*133 genome, and the assembly showed a 99.6% completeness score by analysis of BUSCO genes from the Hypocreales_ODB10 data set (*n* = 4494). BGC prediction of the *Fp*133 genome detected a novel NRPS-associated BGC colocalizing with the apicidin, PKS2, and koraiol BGCs in a region of Chr5 with partial synteny to contig 3 in *Fp*157 (“FDA”, Figure 3C). A PCR assay confirmed the presence of the NRPS gene from this BGC (*fda1*) uniquely in the genomes of all strains denoted as fusadapamide-producing by metabolomics screening (Tables S2 and S3). The encoded NRPS amino acid sequence (Fda1) did not align with any known *Fusarium* NRPS as classified by Hansen et al. 2015.^15^

In addition to the novel NRPS *fda1* (locus ID *FPOAC2_*13301), four neighboring genes were predicted to be involved in fusadapamide production based on transcriptomic analysis of *Fp*133 cultured on media eliciting robust fusadapamide production (Table S4). Genes expressed in the vicinity of *fda1* encoded a major facilitator superfamily transporter (*fda2; FPOAC2_13302*), a FAD-dependent oxidoreductase (*fda3; FPOAC2_13303*), a PLP-dependent enzyme with rhodanese-like domain annotated as belonging to the “cysteine synthase” secondary metabolite gene family or SMCOG 1081 (*fda4; FPOAC2_13305*), and an N-acetyltransferase (*fda5; FPOAC2_13306*) (Figure 4A). Metabolomic profiling of *Fp*133 *Δfda1 - Δfda5* gene deletion mutants (Figures S24–S31) confirmed that *Δfda1* resulted in a total loss of fusadapamide production, confirming this BGC as the fusadapamide BGC. *Δfda2* and *Δfda5* strains were unaffected compared to wild type *Fp*133. Unlike with the *Δfda1* strain, the fusadapamide mass feature signal intensities were not eliminated in the *Δfda3* and *Δfda4* strains. However, they were significantly reduced (*p* < 0.01 for fusadapamide A [M + H]^+^) by approximately 100-fold compared to wild type (Figure 4B). Thus, *fda1*, *fda3* and *fda4* are critical for fusadapamide biosynthesis.

**Figure 4:**
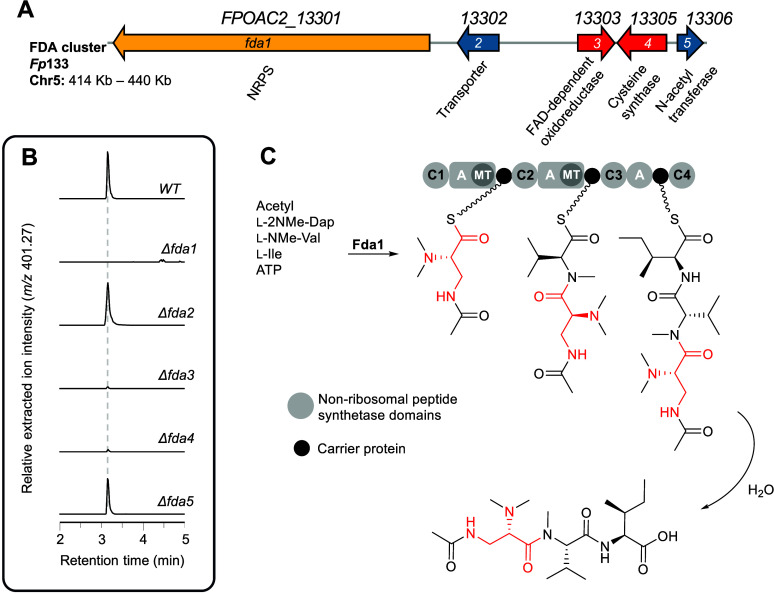
Proposed fusadapamide biosynthesis. (A) The five-gene FDA biosynthetic gene cluster from *F. poae Fp*133, which includes a 2,3-diaminopropionic acid biosynthesis “module” (red genes). (B) Extracted ion chromatograms of *m*/*z* 401.27 (fusadapamide A), comparing wild type (WT) *Fp*133 to FDA biosynthetic gene cluster knockouts in the WT background (*Δfda1–5*). (C) The proposed biosynthesis of fusadapamide. Domain names: C (condensation); A (adenylation); MT (*N*-methyl transferase embedded in A-domain).

### Domain Architecture of the NRPS Fda1

Analysis of the Fda1 NRPS domain architecture supported the proposed structures of fusadapamides. Fda1 is trimodular, comprising three canonical “C-A-T” modules, including two with *N*-methyltransferase (nMT) domains embedded between conserved A8 and A9/A10 motifs of the A-domains (sometimes described as “interrupted” A-domains),^16^ and terminates with a condensation domain (C) (Figure 4C). Fda1 therefore has the following domain architecture: C-A-nMT-T-C-A-nMT-T-C-A-T-C. Consistent with the first two modules containing *N*-methyltransferase domains, the first two residues of fusadamamide are *N*-methylated. The lack of epimerization domains in Fda1 supports the assignment of all amino acids, including Dap, as l configured. Top BLASTp hits to sequences in the highly curated MIBIG 3.0 BGC database indicated the Fda1 A-domains, which are selective for Dap, Val and Val/Ile, closely aligned with *Metarhizium anisopliae* destruxin synthetase A-domains, which are selective for Ala, Val, and Val/Ile. Their amino acid identity matches are between 48.4 to 59.1% (Table S5).^17^ This is consistent with homologous biochemical function.

Although the alignment-based module predictions were consistent with the proposed structure of fusadapamide, the linear nature of fusadapamide appeared at odds with the presence of the Fda1 terminal C domain. Terminal C domains in fungal NRPS systems are typically associated with peptide release via macrocyclization.^18^ To further evaluate the C domain, a phylogenetic analysis of 198 C- and E- (epimerization) domain amino acid sequences from 34 characterized fungal NRPS systems was performed. This analysis showed five clear C-domain clades, including four corresponding to previously documented ^D^C_L_ (condensing d- to l-residues), ^L^C_L_ (condensing l- to l-residues), Ct_(cyc)_ (terminal cyclization domains), and E (epimerization) domain clades,^19,20^ in addition to a new fifth clade, here tentatively named the C_(nMT)_ clade (Figure 5, Table S6; see Supporting Information 2 and additional FASTA alignment provided in Supporting Information). The well-defined C_(nMT)_ clade included the C1 and C2 C domains from Fda1 as well as C-domains from NRPSs associated with biosynthesis of cyclosporine, AbT1, aspergillicin, beauvericin, destruxin, KK1, tentoxin and sansalvamide. This new clade was named based on the presence of nMT domains in all of the corresponding NRPSs, and has not been characterized in previous C-domain phylogenetic analyses (a recent analysis of over 34,000 fungal C domains appears to have filtered out C_(nMT)_ clade domains).^21^ Additionally, *fda1* modules 1 and 2 shared an unusual level of sequence duplication, encompassing approximately 500 base pairs of the C1 and C2 domains, and 1300 base pairs spanning the nMT and T domains (Figure S32). Importantly, the terminal C4 domain from Fda1 did not clade with other fungal Ct terminal cyclization domains. Rather C4 can be found in the ^D^C_L_ branch, underscoring the highly unique nature of the biochemistry catalyzed by this domain.

**Figure 5:**
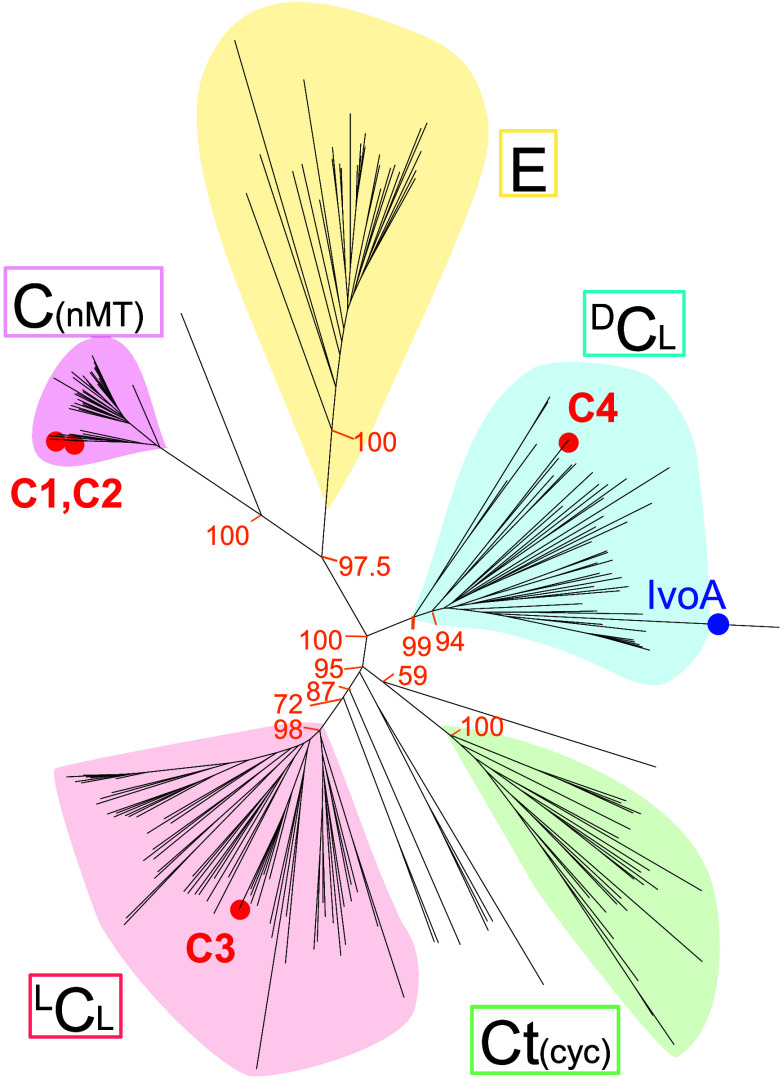
Unrooted maximum-likelihood phylogenetic tree incorporating 198 condensation (C) and epimerization (E) domain amino acid sequences from 34 fungal NRPSs. Red tips and labels are Fda1 C-domains C1–C4; blue tip is the terminal C-domain of *Aspergillus nidulans* IvoA. Phylogeny was constructed with IQTree2 using the LG+F+R9 substitution model. Values at main branchpoints are SH-aLRT bootstrap support values (%, *n* = 1000). Clade colors represent distinct functional clades: Epimerization (E, yellow); C domains in modules with nMT domains (C_(nMT)_, purple), ^D^C_L_ (blue), ^L^C_L_ (red), and terminal C domains associated with product macrocyclization (Ct_(cyc)_, green).

### Role of Dap in Fusadapamide Biosynthesis

Based on the annotation of Fda4 as a PLP-dependent enzyme and Fda3 as an FAD-dependent oxidoreductase, it was hypothesized that these two proteins were responsible for Dap biosynthesis. To test this hypothesis, *Fp*133 *Δfda3* and *Δfda4* strains were inoculated into a culture medium supplemented with exogenous l-Dap. Fusadapamide production was restored in both *Δfda3* and *Δfda4* strains, supporting the involvement of both genes in Dap biosynthesis (Figure 6A). Exogenous l-Dap supplementation also increased fusadapamide production in the WT by approximately two- fold (*p* < 0.01). Repeated feeding experiments with the Δ*fda4* strain, employing isotopically labeled l-Dap (enriched with ^13^C for all C’s and ^15^N for all N’s for a total of +5 amu), further confirmed exogenous l-Dap incorporation into fusadapamides (Figure 6A).

**Figure 6:**
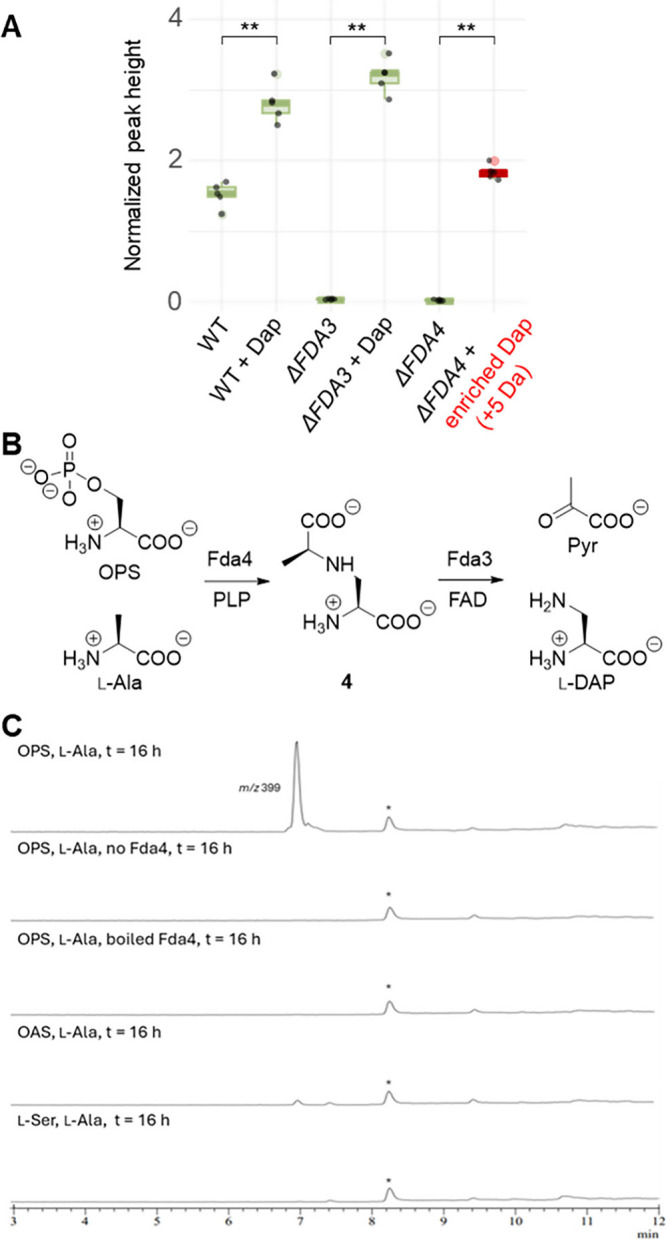
Fda4 and Fda3 are responsible for l-Dap biosynthesis. (**A**) Boxplot comparing fusadapamide A (*m*/*z* 401.27) peak heights from Dap chemical complementation experiments. WT and knockout strains of *fda3* and *fda4* were each fed exogenous l-Dap (*n* = 5). All boxplots peak heights represent the fusadapamide A *m*/*z* 401.27 [M + H]^+^ ion, except for the treatment of *fda4*, which was fed isotopically enriched Dap and produced a signal at *m*/*z* 406.27, which was absent in all other treatments (colored red). *p*-values were measured via the nonparametric Wilcoxon test (** = *p* < 0.01). (B) Biochemical formation of l-Dap from OPS and l-Ala. (C) Recombinant purified Fda4 catalyzes the condensation of OPS (10 mM) with l-Ala (10 mM) to produce the serine-alanine conjugate **4** ([M + H]^+^ *m*/*z* 399). In the absence of Fda4 or in boiled Fda4 treatments, no **4** is produced. Replacement of OPS with OAS (10 mM) produces trace but detectable **4**, and replacement with l-Ser (10 mM) produces no detectable **4**. Buffer conditions: DTT (5 mM), PLP (100 μM), Fda4 (10 μM), KCl (100 mM), phosphate buffer (50 mM, pH 7.4). Reactions were derivatized with Fmoc-Cl and run on LCMS. Ion extractions for Product [M + H]^+^ = 399 shown. *LCMS system peak, not associated with the assay.

### Fda4 Catalyzes Addition of l-Ala to O-Phosphoserine (OPS)

Recombinant purified Fda4 was shown to condense OPS with l-Ala to generate the serine-alanine conjugate (**4**) (Figure 6B). Production of **4** is Fda4 dependent as the no enzyme control and boiled Fda4 treatment do not generate **4** (Figure 6C). Replacement of OPS with O-acetyl serine (OAS) produces only trace amounts of **4**; while, replacement with Ser does not produce detectable amounts of **4** (Figure 6C). Substitution of l-Ala with d-Ala also abolishes production of any product. Interestingly neither l-Asp nor l-Glu can replace l-Ala as a nitrogen source in this first step of Dap biosynthesis (Figures S33 and S34). Fda3, which is annotated as a FAD-dependent oxidoreductase, completes l-Dap biosynthesis via oxidation and subsequent loss of pyruvate. This is consistent with the chemical complementation of the *Δfda3* strain with l-Dap, however the biochemical activity remains to be confirmed *in vitro*.

### Fusadapamide BGC Is Rare in *Fusarium*

Results from the *fda1* PCR screening of 370 *F. poae* strains revealed that ∼6.5% of the sampled population (24/370 strains) had a copy of *fda1*, including strains from three of the five Canadian provinces sampled (Saskatchewan, *n* = 11/35; Manitoba, *n* = 10/85; Ontario, *n* = 3/202; Quebec, *n* = 0/38; Prince Edward Island, *n* = 0/1). None of the *F. poae* strains containing *fda1* were from the population of Eastern Canadian strains previously screened for secondary metabolite production (Table S2).^2^ Additionally, all but one of the *F. poae* strains testing positive for *fda1* also tested positive for the apicidin NRPS *APS1*, indicating a strong linkage exists between the two synthetases. The sampled *F. poae* strain collection included 10 strains isolated from outside Canada, none of which tested positive for *fda1* or *APS1*. From a local BLASTn search querying 1311 *Fusarium* genomes (representing 134 *Fusarium* spp.) downloaded from the NCBI nucleotide repository (accessed December 2022), only two genomes were found to contain the fusadapamide BGC: *F*. *langsethiae* strain MFG 217701 (one of four *F. langsethiae* strains included in the database), and *F. poae* strain NRRL 26941 (Figure 7A). A third genome, identified as *F. camptoceras* strain NRRL 13381, contained homologues of *fda3*, *fda4* and *fda5* at over 80% nucleotide identity, but lacked *fda1* and *fda2* homologues. Therefore, occurrence of the fusadapamide BGC should be considered as rare in the genus *Fusarium*, including within producing species populations (such as *F. poae* and *F. langsethiae*).

**Figure 7:**
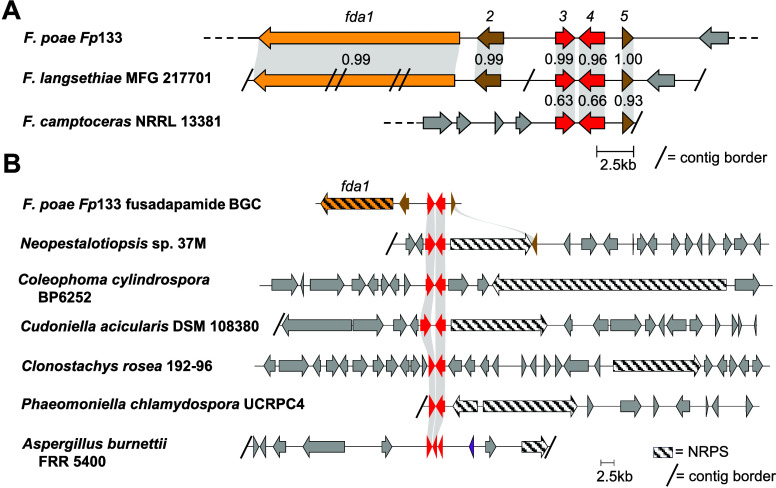
FDA cluster and Dap biosynthetic module in other ascomycete genomes. (A) Comparison of fusadapamide BGC to similar clusters in *Fusarium* genomes. The depicted *F. langsethiae* cluster was manually assembled from 5 short contigs, as the genome is highly fragmented. To achieve complete coverage of the query *fda1* gene, a small *F. langsethiae* contig was duplicated, spanning the duplicated regions of *fda1* modules 1 and 2. Gray genes are not predicted to be associated with secondary metabolite biosynthesis. Light gray blocks connect genes with similar predicted protein sequences with overlaid numbers representing percent identity match of the associated amino acid sequences. (B) Detected NRPS biosynthetic gene clusters encoding the putative diaminopropionic acid (Dap) biosynthesis module from diverse fungi. Dap biosynthesis module genes are colored red; NRPSs are striped. Light gray ribbons connect BLASTp hits with greater than 30% amino acid identity matches over 70% of the length of the query sequence. The NRPS sequences and domain structures are divergent between taxa, and their products are not known.

### Dap Biosynthesis Is Predicted in Fungal NRPS-Containing Gene Clusters

Blastp queries seeking colocalization of Fda3 and Fda4 encoding homologues in the genomic neighborhood of any NRPS in the NCBI protein sequence repository returned hits in six fungal genomes (Figure 7B). The hits are from diverse Ascomycete genera, including *Neopestalotiopsis*, *Coleophoma*, *Cudoniella*, *Clonostachys*, *Phaeomoniella* and *Aspergillus* (Table S7). Two of the six genomes also contained Fda5 encoding homologues. The associated NRPSs in these genomes had low similarity to Fda1, suggesting they produce structurally diverse, Dap-containing peptides.

### Fusadapamide A Does Not Possess Antibiotic Activity

The ability of fusadapamide to inhibit bacterial growth was evaluated using a standardized microtiter dilution assay. At up to a maximum of 10 μM, fusadapamide A did not inhibit the growth of plant-associated, Gram-negative bacterial strains *Pseudomonas syringae pv*. tomato (DC3000), *Pontoea allii* (13–12A) isolated from a wheat leaf, *Pseudomonas trivializ* (LMG2146, type) and *Pseudomonas tolaasii* (LMG2432, type), nor did it inhibit growth of human pathogenic bacterial strains *Salmonella enterica diarizonae* (ATCC 12325), *Escherichia coli* (ATCC 35218) and *E. coli* (O157:H7). Furthermore, at tested concentrations of up to a maximum of 80 μM, fusadapamide did not inhibit the growth of *E. coli* (BW25113) and *Pseudomonas aeruginosa* (PA01) and the Gram-positive bacteria *Bacillus subtilis* (168) and methicillin resistant *Staphylococcus aureus* (USA300).

### Oat Infection Assays Do Not Reveal Differences between Groats of Plants Infected with *Fp*133 WT vs *Fp*133Δ*fda1*

Growth chamber challenge experiments in oat plants were conducted at early anthesis using point-inoculation of *Fp*133 WT and *Fp*133Δ*fda1* strains to compare the extent of discoloration and overall weight loss on mature groats as compared to uninfected controls. The results indicated that despite the loss of fusadapamide production, Δ*fda1* mutants are still capable of colonizing oat plants. Groats from plants inoculated with *Fp*133 WT or *Fp*133Δ*fda1* spores had statistically negligible groat weight loss (between 2 and 9%) compared to mock water-inoculated controls (*p* = 0.061, Kruskal–Wallis nonparametric test). Groats from plants inoculated with *Fp*133 WT or *Fp*133Δ*fda1* had equal groat coat discoloration frequencies (approximately 67%, *p* = 0.8 by nonparametric pairwise Wilcoxon test), which significantly differed from healthy, mock-inoculated groats (*p* = 0.01 by Krusal-Wallis nonparametric test) (Figure S35). Given the very subtle and inconsistent disease phenotypes of oats inoculated with *F. poae*, this data is inconclusive regarding the role fusadapamide A may play in promoting observable disease symptoms in oat caused by *Fp*133.

### l-Dap Biosynthesis in the Fungal Kingdom

In this study, fusadapamides were discovered by metabolomic profiling of a population of *F. poae*, a species shown to contain highly dynamic accessory chromosomes with active secondary metabolite biosynthetic gene clusters. The fusadapamides all contain l-Dap, an unusual and rare nonproteinogenic amino acid, the biosynthesis of which was not known from a fungal source prior to this study. l-Dap is an important structural feature in natural products from bacteria and plants that exhibit neurotoxic,^22^ antioomycete,^23^ anticancer,^24,25^ antibiotic,^26,27^ and siderophore^28^ activities. During bacterial l-Dap formation by *Staphylococcus aureus,* ^9^ SbnA, a PLP-dependent cysteine synthase, produces a serine-glutamate conjugate from OPS and l-Glu. SnbB, a NAD^+^-dependent oxidoreductase, then oxidatively hydrolyzes the serine-glutamate conjugate producing free l-Dap and α-ketoglutarate.^9^ In the legume grass pea, O-acetyl-l-Ser (OAS) is condensed with the l-Asn derivative isoxazolin-5-one to generate the β-amino substitution during production of the neurotoxin β-N-oxalyl-l-α,β-diaminopropionic acid (β-ODAP).^29^ Aspergillomarasmine A synthase, a fungal PLP-dependent cysteine synthase-like homologue that has been shown to produce Toxin A and aspergillomarasmine A in the fungus *Aspergillus oryzae*, condenses OPS and l-Asp (Figure 8), but does not produce free l-Dap.^30^ In contrast to the above biochemical pathways, *F. poae* Fda4 condenses OPS with l-Ala to generate a serine-alanine conjugate, which is oxidized by Fda3 to form l-Dap and pyruvate. The use of l-Ala by Fda4 represents a unique nitrogen donor for this β-substitution biochemistry.

**Figure 8:**
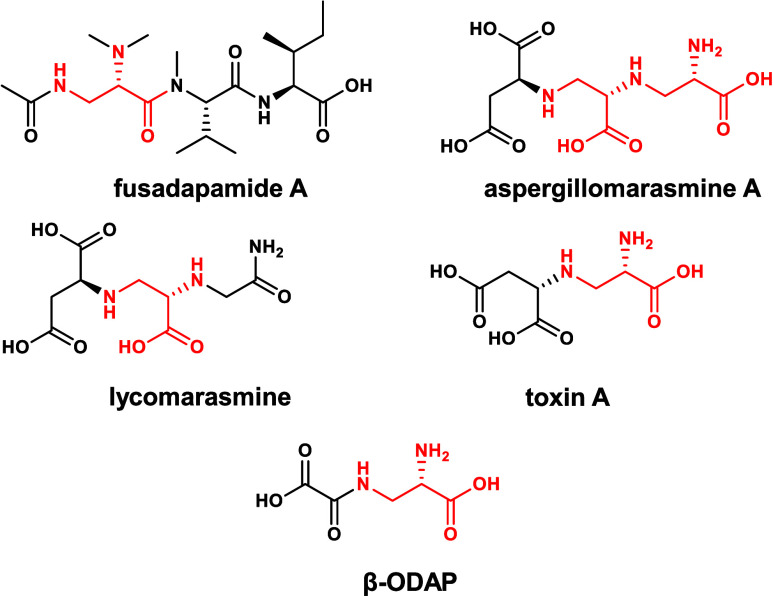
Molecules containing l-Dap substructures. l-2,3-Diaminopropionic acid substructures colored red.

Natural products from the fungal kingdom containing l-Dap substructures are considered rare and are uniquely found in the ‘marasmin’ molecular family, which includes aspergillomarasmine A. Marasmins are metal-chelating natural products detected from agronomically relevant fungal plant pathogens, including aspergillomarasmines from *Aspergillus ssp*., lycomarasmine from *F. oxysporum f.sp. lycopersici* and ‘toxins A-D’ from *Pyrenophora teres* (Figure 8).^31–34^ Interestingly, *F. poae* genomes also exhibit potential for lycomarasmine production, as they contain a two-gene cluster homologous to the recently characterized aspergillomarasmine BGC in *A. oryzae* (Figure S36).^30^

### Fungal NRP Biosynthesis Insights from the Fda1 Domain Structure

In addition to the discovery of a novel biochemical pathway in fungi, the analysis of *F. poae* accessory chromosome-associated BGCs advances our understanding of fungal NRPS-associated biosynthesis. The domain-level architecture of Fda1 has at least two features worth consideration in the context of fungal NRPS evolution. First, the presence of an atypical terminal condensation domain in Fda1 implies fusadapamides are offloaded from Fda1 via condensation with a water molecule. Free acid release via a C-domain is rare among NRPS systems. Although a few bacterial NRPSs have demonstrated this functionality,^35^ only a single fungal natural product is reportedly produced by a condensation domain-associated free acid release. The single-module synthetase IvoA in *Aspergillus nidulans* offloads d-tryptophan in free acid form^36^ via a terminal C-domain which clustered in the ^D^C_L_ amino acid sequence clade as reported in this study (Figure 5). The IvoA terminal C-domain differs from Fda1 C4, however, in that it is preceded by an epimerization domain. Further characterization of the Fda1 C4 domain is necessary to substantiate its proposed role in fusadapamide offloading. The prediction of hydrolyzing terminal C-domains will be important for predicting NRPS product scaffolds as cyclic or linear.

The other unusual feature of the proposed fusadapamide biosynthesis is the α-dimethylation and β-acetylation of Dap by Fda1 module 1. The *N*-methyltransferase domain presumably dimethylates the α-amino of T-bound Dap. Dimethylation is particularly rare and thus detailed characterization of this biochemistry will help illuminate control mechanisms in NRPS *N*-methyltransferases. The source of Dap β-acetylation is yet to be resolved. This could either be catalyzed by the Fda1 C1 domain or by the putative N-acetyl-transferase Fda5. Although the latter possibility is not seemingly supported by the observation that the *Fp133Δfda5* mutant produced fusadapamides at levels comparable to the wild-type strain, it is possible that other N-acetyltransferases compensate for the loss of *fda5*. At least nine additional *Fp133* proteins contain similar predicted N-acetyltransferase domains and are expressed under fusadapamide-producing conditions (Table S8). Furthermore, characterization of the C_(nMT)_ clade of fungal C-domains associated with NRPSs containing *N*-methyltransferase domains (including Fda1 C1 and C2) may provide insight into the fundamental biochemistry of amide bond formation involving *N*-methylated amino acid nucleophiles and/or the spatial accommodation of interrupted A domains in NRPS proteins. Thus, while fusadapamides possess relatively common functional groups, it is clear that fusadapamide biosynthesis utilizes a number of atypical biosynthetic functions, characterization of which expands upon our current understanding of NRPS biosynthesis.

### Accessory Chromosomes and NRPS Evolution in *Fp*133

The fusadapamide BGC is located within a BGC-rich region of Chr5 in *Fp*133. *Fp*133 Chr5 and Chr6 are here labeled as “accessory chromosomes” due to patterns in their genetic content being suggestive of the accessory genome in fungi (Table S1). The structural dynamism of *F. poae* accessory chromosomes is evident in Chr5, which contains two centromere-like sequences, suggesting its formation via the fusion of two smaller chromosomes – a process also proposed to account for the length and polymorphism distribution in *F. graminearum* chromosomes,^37^ with which *F. poae* core chromosomes share macrosynteny.^5^ Extensive repetitive sequences on *Fp*133 accessory chromosomes suggest Repeat-Induced Point mutation (RIP), a premeiotic genome defense mechanism which induces nonsense mutations in repetitive DNA in certain fungi,^37,38^ is inactive in *F. poae.* Speculatively, detection of a duplicated nucleotide sequence (∼1.3 Kb) within *fda1* (Figure S32) indicates any advantage gained by fusadapamide production could be lost by RIP-associated destruction of *fda1* during meiosis, a scenario which could help explain *fda1*’s rarity in *Fusarium*, although meiosis frequency has not yet been investigated in *F. poae* populations.

### Addressing Challenges in *F. poae* Research Systems

Elucidating the biological function of fusadapamide A presents a considerable challenge due to the complex and understudied nature of *F. poae* colonization of host plants, including the agronomic crops from which the strains used in this study were isolated. Despite these challenges, the discovery of lineage-specific *F. poae* mycotoxin production has potential health implications for consumers^2^ and underscores an urgent need for continued analyses of *F. poae* chemical phenotypes at the population level, both in Canada and globally.

Uncovering the extent of accessory chromosome occurrence within *F. poae* populations necessitates a phylogenetic analysis of this species’ population structure to be completed, ideally using a population representing a greater global distribution. Moving forward, further investigation into the biological functions of fusadapamides, their potential role in plant colonization by *F. poae*, and the evolutionary mechanisms shaping NRPS gene diversity in the accessory genome, will be essential for a deeper understanding of fungal natural product biosynthesis and plant-fungal interactions. Excitingly, the discovery of a Dap biosynthetic module ortholog in diverse fungal genera (Figure 7B) suggests more Dap-containing fungal natural products await discovery.

## Experimental Section

### General Experimental Section

Twenty-three Fusarium poae strains isolated from FHB surveys of cereals in Saskatchewan were obtained from the research culture collection of Dr. Allen Xue,^39^ and 91 F. poae strains isolated from FHB surveys of cereals in Manitoba/Saskatchewan were obtained from the research culture collection of Dr. Xiben Wang.^40^ See Table S2 for a list of strains included. All but four strains were isolated from oats (*Avena sativa*). Both culture collections are maintained at Agriculture & Agri-Food Canada in Ottawa, ON and Morden, MB, respectively. Of these, 21 strains were selected for intensive metabolomic profiling (6 from Saskatchewan, 15 from Manitoba). Strain selection was based on geographic location, host plants, a preliminary genetic test for divergence, and diversity of metabolomic features detected using ultrahigh performance liquid chromatography coupled to high resolution mass spectrometry (UPLC-HRMS) analysis of extracts from strains grown on a single media condition (MMK2).

Detailed protocols for DNA extractions, PCR and Sanger sequence analysis confirming the species identification of all isolates were performed as published previously.^2^

### Untargeted Metabolomics Profiling

The extraction and metabolomic profiling of strains using ultrahigh pressure liquid chromatography coupled to high resolution mass spectrometry (UPLC-HRMS) was performed exactly as detailed in Witte et al. (2021) with the exception that the media condition “S2M” was not included in the profiling of the Western Canadian *F. poae* strains, as it was determined that this media condition induced minimal chemical phenotype profiles.^2^ Data from Witte et al. 2021 was reanalyzed in this analysis to produce Figure 1. In brief, strains were inoculated into 4 different liquid media conditions (CYA, MMK2, YES and YESIO; see Supporting Information 1 for media formulations) in triplicate, and then incubated in the dark for 14 days. Mycelium and spent broth were then separated and extracted in ethyl acetate for 1 h. Organic solvents were transferred to borosilicate scintillation vials, dried under vacuum, reconstituted in methanol and analyzed on a Thermo Ultimate 3000 UPLC coupled to a Thermo LTQ Orbitrap XL equipped with a THERMO Dionex Ultimate 3000 Diode array detector (190–800 nm). A Phenomenex C18 Kinetex column (50 mm × 2.1 mm ID, 1.7 μm) was used for chromatography with a flow rate of 0.35 mL/min, running a gradient of water (+ 0.1% formic acid) and acetonitrile (+ 0.1% formic acid): starting at 5% acetonitrile increasing to 95% acetonitrile by 4.5 min, held at 95% acetonitrile until 8.0 min, returning to 5% acetonitrile by 9 min and held to 10 min to equilibrate the column to starting conditions. The HRMS was operated in ESI+ mode (monitoring a range of 100–2000 *m*/*z*).

Mass spectrometry data preprocessing was performed using MZMine 2.37 with Ion Identity Networking module.^41,42^ For more detailed parameters please see.^2^ The preprocessed raw data files were converted into a data matrix of discriminate variables consisting of retention time (RT) and *m*/*z*. Variables are referred to here as mass features. Data was imported into the R environment, where mass features representing associated adducts and in-source fragments of the same parent ion were grouped using Pearson correlation analysis of exported and normalized peak areas over a sliding window of elution time. These groupings were compared to data generated during raw data preprocessing via the Ion Identity Networking module which correlated peak shapes of coeluting signals.^42^ Mass feature data from the two extraction types (mycelium and broth) were summed, converted to binary form, and then averaged across the four media conditions to form a ‘pseudobinary’ matrix of detection frequencies for each mass feature. Features were then annotated either by comparison to purified or commercial lab standards: 3A-deoxynivalenol was purchased from Sigma-Aldrich (St. Louis, USA), and beauvericin, 3,15-diacetoxyscirpenol, 15-monoacetoxyscirpenol, neosolaniol, fusarenon-X and nivalenol were purchased from Fermentek (Israel). All remaining annotations were given based on exact mass comparison to known *Fusarium* secondary metabolites and by in silico based structural predictions or by comparison to experimentally derived molecular fragmentation patterns from annotated molecules.^43,44^ Mass features were filtered for visualization based on their relevance as either annotated features or unannotated features with high intensities. All heatmaps were produced using the ComplexHeatMap package^45^ in R.

### Oat Growth Chamber Experiments

Oat seed from two variety lines, RC Amaze and Makuru, were surface-sterilized in 2% bleach, rinsed and planted in 6-in. pots for growth in cabinets set to 25 °C daytime and 20 °C night temperature cycles, with a 16 h photoperiod and with 360 mol m^–2^s^–1^ light intensity. The plants were fertilized at 5 weeks with 20–20–20-NPK. Two sets of pathogenicity trials were carried out: a preliminary experiment was attempted first using the wildtype (WT) strain *Fp*133 (harvested 21 days post inoculation/day 73 after planting); and a second experiment using *Fp*133 WT and *Fp*133*Δfda1* using RC Amaze plants only (harvesting at plant maturity/day 91 after planting, with watering stopped on day 81). Plants were point inoculated at midanthesis (day 52 after planting) with 10 μL of spore suspension ([5.0 × 10^4^ conidia/mL] in sterilized water; ∼500 conidia per spikelet). Control treatments were prepared using sterile water without conidia added. The control plants were covered during inoculations and for a week immediately afterward.

At harvest, representative groats from each treatment were hulled, counted, photographed, weighed and sorted into “normal” or “discolored” based on visual comparison to noninoculated groats. To confirm successful colonization by the inoculated strain (Koch’s postulates), four groats from each treatment (two discolored and two normal, or four normal from the mock water treatment) were surface sterilized and germinated in Petri dishes containing PDA medium with 100 μg/mL chloramphenicol. Mycelia grew only from the roots and shoots of germinated seedlings obtained from discolored groats from spikelets inoculated with fungal conidia. PCR amplification of primers for *APS1, Fda1* and the Hygromycin B resistance gene from genomic DNA isolated from the mycelia indicated successful colonization of the intended fungal strains. For metabolite analysis, approximately 15–20 whole spikelets (groats and glumes) from each treatment were flash frozen in liquid N_2_ and stored at −80 °C, before being ground in liquid N_2_ (3 replicates per pot). Ground tissues were lyophilized for 48 h and a 500 mg aliquot was extracted with 4.0 mL of 100% acetonitrile + 0.1% formic acid (to which 1 ppm reserpine was added) in borosilicate glass scintillation vials by shaking (150 rpm) at room temperature for 1 h, centrifuged for 20 min on a Genevac centrifuge (2150 rpm), and profiled by UPLC-HRMS using the same chromatography and HRMS parameters as described for the untargeted metabolomics screening methods.

### Targeted Metabolomics Analysis of Oat Extracts

The HRMS data raw files from the oat extracts were preprocessed using MZMine v2.37 using the ‘Targeted peak detection’ module, supplied with a list of target mycotoxin masses and associated retention times. Apicidin mass feature annotation was as described in Witte et al. 2021. Noise intensity cutoff was set at 1.0E3. All predicted peaks were manually checked against the raw data using extracted ion chromatograph visualization in Thermo Xcalibur. Peaks which were inconsistent across replicates, or which only occurred at trace levels below the 1.0E3 noise threshold were not included in the reported profiles. Trace or inconsistent signals included those with matching *m*/*z* and RT to 15-monoacetoxyscirpenol, fusarenon-X, neosolaniol and nivalenol. Fusarins were not detected in the plant extracts.

### Genomic DNA Isolation, Sequencing, and Assembly

Genomic DNA (gDNA) extraction and sequencing for both Illumina NextSeq 500 and Nanopore MinION was performed exactly as described previously for sequencing of *Fp*157.^2^ Assembly of the Nanopore long-read sequences was performed using CANU v1.8 ^46^ using default settings with an estimated genome size of 40 Mb (genomeSize = 40m). Nanopolish v. 0.11.1 was then used to correct the CANU assembly, followed by one round of Pilon v1.23 correction.^47^ The resulting contigs were then aligned and compared with those of isolate *Fp157* ^2^ to identify chromosomes 1–4 and the mitochondrial genome. The CANU-generated assembly was compared to a second, *de novo* assembly generated using Flye v2.9.^48^ The final assembly was created by merging contigs wherever possible based on consensus between the two assembler outputs. To test the finalized assembly, the corrected and trimmed nanopore reads were mapped to the assembly using minimap2,^49^ and the coverage was inspected at each merged site by visualizing the mapped reads using Geneious Prime v 2022.1.1.

*Fp*133 accessory chromosome lengths were confirmed by chromosome size estimation using contour-clamped homogeneous electric field (CHEF) electrophoretic karyotyping of lysed protoplasts under optimized conditions. Full methodological details are provided in Supporting Information 1.

### *Fp*133 Gene Locus Prediction, Annotation, and Centromere Prediction

The *Fp*133 assembly was repeatmasked, gene predicted and annotated using the RepeatModeler,^50^ funannotate (v1.8.14) and Antismash (v7.0.0) pipelines. Full methodological details are provided in Supporting Information 1. Core chromosome centromere predictions were compared to equivalent centromere locations and lengths in *F. graminearum*.^51^

### PCR Assay for *fda1/APS1* Gene Detection

The methods used to detect *APS1* and *fda1* by PCR were as published.^2^ In short, primers were designed and used in a duplex reaction alongside primers designed to amplify the *TEF1α* gene (see Table S3 for all PCR primers used in this research). *TEF1α* was used as a positive control to ensure the DNA was amplifiable.

### Comparison of Nucleotide Diversity between Fda1 Modules

The NRPS module DNA sequences were extracted along the borders defined by antiSMASH, and compared using DnaSP v6.12.03, averaging nucleotide diversity values over a 100 bp sliding window measured in 25 bp steps.^52^ The linegraph visualization was produced in R.

### Fungal C-Domain Analysis

34 fungal NRPS annotations were sourced from the MIBIG v3.0 database of natural product BGCs^53^ and from manually annotated NRPS genes from *Fp*133 (fusadapamide and fusahexin) and *Alternaria tennuisima* 1166 (tentoxin) (Table S6). C-domain borders were manually defined for each module, as extending from the antiSMASH-annotated C2 motif to the adjacent A-domain motif. C-domains were classified into four categories based on the architecture of the encompassing NRPS module, including: (i) immediately after an epimerization domain, suggesting ^D^C_L_ functionality; (ii) in an NRPS module with an *N*-methyl transferase; (iii) terminal to an NRPS, or (iv) all other condensation domains (assumed to be ^L^C_L_). All annotated epimerization (E) domains were also included, as well as potentially pseudogenized epimerization domains (“E_pseudo”) which were predicted to lack key conserved motifs by antiSMASH. In total there were 198 domains included in the analysis. Details pertaining to sequence alignment and tree building methodology, including Newick format maximum-likelihood tree text can be found in the Supporting Information.

### CRISPR-Cas9 Gene Editing and Metabolomic Profiling of Gene Deletion Mutants

Targeted gene deletions in *Fusarium poae* strain *Fp*133 were performed using a CRISPR-Cas9 ribonucleoprotein (RNP)-mediated approach with homologous-directed repair (HDR), as described in Hicks et al. 2023.^54^ Full details of the CRISPR-mediated gene editing protocol, including crRNA sequences, HDR primer design, and transformation conditions, are provided in Supporting Information 1.

To determine whether the gene deletion mutants produced fusadapamide during axenic culturing, agar plugs were inoculated from strains grown on SNA media into 15 mL of YES broth in slant tubes. Growth, extraction and processing parameters were exactly as described as above for the untargeted metabolomics experiments. The metabolomics protocol was repeated for *Δfda3* and *Δfda4* on two media types (MMK2 and YES) in quintuplicate, to confirm of the low fusadapamide signals detected from those gene deletion mutants.

### Fusadapamide Isolation, NMR Structural Elucidation, and Marfey’s Method Details

Conidia from *F. poae* isolate *Fp*133 were inoculated into 4 L of Minimal Media broth (final concentration 1.33 × 10^6^ conidia/mL) containing 5 g of^15^ N-labeled NaNO_3_ (Sigma-Aldrich) in addition to 3 g of unlabeled NaNO_3_ (for a total of 8 g), divided in 15 mL slant tubes and incubated in the dark at 25 °C for 15 days. The broth was then filtered to remove mycelium to which 250 g of activated Diaion HP20 resin beads were added and the mixture was shaken for 1 h on a rotary shaker (250 rpm). The HP20 resin was then vacuum filtered from the broth, washed with 500 mL of deionized water, and then extracted three times in 300 mL of HPLC-grade methanol. The methanol fractions were combined and rotary-evaporated under vacuum at 30 °C, yielding approximately 280 mg of crude extract. The crude extract was then reconstituted in 10% acetonitrile/water and fractionated by preparatory HPLC (Agilent Technologies 1260 Infinity) on a Kinetex 5 μm C18 100 Å LC Column (100 × 30 mm) with an ACN/water gradient (flow rate of 20 mL/min) beginning at 5% ACN for 2 min, ramping to 30% ACN from 2 to 6 min, then to 50% ACN from 6 to 8 min, and last up to 100% ACN over 8–12 min, before returning to start conditions. Fractions were collected every 0.2 min using an Agilent Technologies 1260 Infinity preparatory-scale fraction collector and a representative 0.5 mL aliquot of each fraction was reserved for UPLC-HRMS analysis. Collected fractions were transferred to weighed borosilicate scintillation vials and dried under vacuum.

NMR spectra were recorded on a Bruker AVANCE III 600 MHz spectrometer with a cryoprobe at the University of Ottawa, Ottawa, Canada. Fraction 39 was dissolved in deuterated DMSO, to which the spectral signals were referenced. NMR data was processed and visualized in MestReNova v14.2.0 (Mestrelab).

For the assignment of residue stereochemistry, Marfey’s method was used. Full hydrolysis was performed on 0.25 mg of each purified compound, dissolved in 100 μL of 6 M HCl made with 1:1 D_2_O:H_2_O and shaken in a sealed tube on a thermomixer at 100 °C for 24 h. The hydrolyzed samples were dried under N_2_. Samples were derivatized by the addition of 20 μL of 1 M NaHCO_3_ and 380 μL of l-FDAA stock solution (6 mg in 5.7 mL acetone) followed by incubating at 40 °C for 1 h. The reactions were cooled to room temperature and then neutralized with 20 μL of 1 M HCl. The samples were then filtered through 0.2 μM PTFE syringe filters into fresh vials, and injected into the UPLC-HRMS using a gradient starting at 5% acetonitrile with 0.1% formic acid (solvent B), increasing linearly to 40% B over 55 min, increasing to 100% B over 3 min and held for 3 min before returning to initial conditions. For dimethylated l-Dap standards synthesis details, see Supporting Information 1.

#### Fusadapamide A (**1**)

Colorless oil (enriched fraction); UV (1:4 ACN:H2O) λ_max_ 210; HRMS (ESI-orbitrap) *m*/*z* 401.2763 [M + H]^+^ (calcd for C_19_H_37_N_4_O_5_^+^, 401.2759); ^1^H NMR ((CD_3_)_2_SO, 600 MHz): δ_H_ 8.33 (d, 1H, *J* = 8.6 Hz), 7.76 (t, 1H, *J* = 5.9 Hz), 4.07 (dd, 1H, *J* = 8.5, 5.5 Hz), 3.80 (d, 1H, *J* = 10.8 Hz), 3.70 (dd, 1H, *J* = 8.8, 4.0 Hz), 3.59 (m, 1H), 3.19 (m, 1H), 2.63 (s, 3H), 2.37 (s, 6H), 2.16 (m, 1H), 1.72, (s, 3H), 1.66 (m, 1H), 1.37 (m, 1H), 1.02 (m, 1H), 0.89 (d, 3H, *J* = 6.4 Hz), 0.8 (t, 3H, *J* = 7.4 Hz), 0.77 (d, 3H, *J* = 6.9 Hz), 0.69 (d, 3H, *J* = 6.8 Hz). ^13^C NMR ((CD_3_)_2_SO, 151 MHz): δ 173.7, 170.6, 170.0, 168.2, 65.2, 62.2, 57.7, 40.7, 38.4, 32.9, 28.4, 25.7, 24.6, 22.0, 18.1, 16.0, 12.1

#### Fusadapamide B (**2**)

Colorless to pale yellow oil (partially purified fraction); UV (1:4 ACN:H2O) λ_max_ 210; HRMS (ESI-orbitrap) *m*/*z* 401.2761 [M + H]^+^ (calcd for C_19_H_37_N_4_O_5_^+^, 401.2759); ^1^H NMR ((CD_3_)_2_SO, 600 MHz): δ_H_ 8.32 (d, *J* = 8.7 Hz, 1H), 7.73 (t, *J* = 5.9 Hz, 1H), 4.05 (q, *J* = 8.0 Hz, 1H), 3.78 (d, *J* = 10.9 Hz, 1H), 3.73 (dd, *J* = 8.7, 3.9 Hz, 1H), 3.61 (ddd, *J* = 11.2, 6.8, 4.0 Hz, 1H), 3.19 (ddt, *J* = 12.9, 8.8, 4.6 Hz, 1H), 2.63 (s, 3H), 2.35 (s, 6H), 2.18–2.14 (m, 2H), 1.73 (s, 3H), 1.50–1.43 (m, 2H), 1.29 (t, *J* = 8.2 Hz, 1H), 0.88 (dd, *J* = 8.6, 6.4 Hz, 3H), 0.86–0.77 (m, 6H), 0.69 (dd, *J* = 6.8, 2.9 Hz, 3H). ^13^C NMR ((CD_3_)_2_SO, 151 MHz): δ 74.4, 64.7, 61.7, 51.8, 42.4, 39.5, 32.1, 27.6, 25.0, 24.1, 22.5, 22.1, 19.6, 17.3 (HSQC-assisted carbon assignments; weak ^13^C S/N in fraction; partial set).

#### Fusadapamide C (**3**)

Colorless to pale yellow oil (partially purified fraction); UV (1:4 ACN:H2O) λ_max_ 210; HRMS (ESI-orbitrap) *m*/*z* 387.2604 [M + H]^+^ (calcd for C_18_H_35_N_4_O_5_^+^, 387.2602); ^1^H NMR ((CD_3_)_2_SO, 600 MHz): δ 8.32 (d, *J* = 8.7 Hz, 1H), 7.73 (t, *J* = 5.9 Hz, 1H), 4.05 (q, *J* = 8.0 Hz, 1H), 3.78 (d, *J* = 10.9 Hz, 1H), 3.73 (dd, *J* = 8.7, 3.9 Hz, 1H), 3.61 (ddd, *J* = 11.2, 6.8, 4.0 Hz, 1H), 3.19 (ddt, *J* = 12.9, 8.8, 4.6 Hz, 1H), 2.63 (s, 3H), 2.35 (s, 6H), 2.18–2.14 (m, 1H), 1.73 (s, 2H), 1.50–1.43 (m, 3H), 1.29 (t, *J* = 8.2 Hz, 1H), 0.88 (dd, *J* = 8.6, 6.4 Hz, 4H), 0.86–0.77 (m, 7H), 0.69 (dd, *J* = 6.8, 2.9 Hz, 3H).

### Dap Feeding Assays

Wild-type *Fp*133, Fp133Δ*fda3* and Fp133Δ*fda4* conidia were inoculated into 2 mL of liquid YES broth at either 10 mM l-Dap or without l-Dap added (final concentration of conidia was 5 × 10^3^ spores/mL). For the Fp133Δ*fda4* culturing, 10 mM of isotopically enriched l-Dap was used (all carbons and nitrogens enriched with ^13^C and ^15^N respectively); all other treatments were supplemented with filter-sterilized, commercially available l-2,3-Diaminopropionic acid hydrochloride (Santa Cruz BioTechnology, California). Incubation, extraction and UPLC-HRMS protocols were as described above, with the exception that only broths were extracted, and resuspensions were performed in 100 μL of a stock solution of methanol with 5 μM reserpine (for subsequent data normalization). Isotopically labeled l-Dap was synthesized using enriched asparagine as a precursor.

### Cloning, Expression, and Purification of Fda4

The *fda4* gene was synthesized as a codon-optimized, intron-free sequence and cloned into the pET28a expression vector using restriction enzyme digestion and ligation. The recombinant plasmid (pLAP01) was transformed into *E. coli* XL1-Blue, confirmed by Sanger sequencing, and used for heterologous expression. For protein expression, *E. coli* BL21 (λDE) cells were transformed with pLAP01 and cultured in LB medium with kanamycin. Gene expression was induced with IPTG at mid log phase, and the culture was incubated at room temperature for 18 h. Cells were harvested, lysed by sonication, and the soluble fraction was purified using Ni-NTA affinity chromatography. The recombinant Fda4 protein was eluted with imidazole, concentrated, and buffer-exchanged into phosphate buffer. Protein concentration was determined by Bradford assay. Full experimental details, including primer sequences, enzyme conditions, and purification steps, are provided in Supporting Information 1.

### In Vitro Fda4 Substrate Specificity Assays

Enzyme assays were performed in 50 mM phosphate buffer pH 7.4, 100 mM KCl, 5 mM DTT, 100 μM PLP, 10 μM Fda4, and 10 mM of l-Ser, O-phospho Ser, or O-Acetyl Ser and 10 mM of the nitrogen nucleophile (either l-Ala, d-Ala, l-Glu or l-Asp). The reactions were incubated overnight at room temperature. Fmoc-Cl derivatization quenched the reactions which were analyzed by LCMS. Extracted ion chromatograms were extracted for the expected products. For further details on Fmoc-Cl derivatization and LCMS analysis of *in vitro* assays; see Supporting Information 1.

### Minimum Inhibitory Concentration Determination via Microtiter Broth Dilution

For assessing the antibacterial effect of fusadapamide A, bacterial strains (*Bacillus subtilis* 168, Methicillin resistant *Staphylococcus aureus* USA300, *Pseudomonas aeruginosa* PA01, *Escherichia coli* BW25113) were plated on LB-agar plates and grown overnight. Fusadapamide A stocks were prepared to 64 μg/mL in Mueller Hinton (MH) broth. MH broth was added to 96-well plates with the first wells containing 32 μg/mL (80 μM) fusadapamide A in triplicate. The wells were diluted in series to 0.125 μg/mL, including no bacteria and no fusadapamide A controls in final wells. Bacteria were prepared in MH broth to an OD600 = 0.070 and added to all wells except for the no bacteria control. The 96-well plates were incubated at 37 °C overnight and the presence of bacterial growth was analyzed by measuring the OD600.

In addition, plant-associated bacterial strains *Pseudomonas syringae pv* Tomato strain DC3000 (LMG1247), *Pontoea allii* strain 13–12A isolated from wheat leaf (Alberta; LMG24248), *Pseudomonas trivializ* type strain (LMG2146) and *Pseudomonas tolaasii* type strain (LMG2432) (graciously provided by Dr. James Tambong at the Ottawa Research and Development Center, Agriculture and Agri-Food Canada), and human pathogenic bacterial strains *Salmonella enterica diarizonae* (ATCC 12325), *E. coli* ATCC 35218 and *E. coli* O157:H7 were also treated with fusadapamide A at 0.1, 1, and 10 uM final concentration, using the same methods as above with the exception that plant associated bacteria were incubated at 28 °C instead of 37 °C.

## Supplementary Material





## Data Availability

Raw reads from Illumina, Nanopore, and RNaseq gene expression data are all available at the NCBI SRA and are linked to bioproject accession PRJNA924782. The assembled and annotated genome of Fp133 has been deposited at genbank with accession number JBJHSE000000000. The NMR data for the following compounds have been deposited in the Natural Products Magnetic Resonance Database (NP-MRD; https://np-mrd.org): fusadapamide A, fusadapamide B, and fusadapamide C.

## Notes

Experimental procedures; Newick tree (condensation-domain phylogeny); NMR spectra of fusadapamides A–C; NMR spectra of compound **4** (α-*N*-dimethyl Dap) and compound **5** (β-*N*-dimethyl Dap); MS/MS fragmentation of fusadapamide A; Marfey’s analysis of fusadapamides; CHEF gel confirmation of Fp133 small chromosome sizes; PCR primer design strategy; PCR/Southern blot validation of FDA BGC deletion mutants; comparison of *fda1* modules 1 and 2; Fda4 expression and in vitro nitrogen source characterization; oat groat mass and discoloration after *F. poae* infection; synteny analysis of the *F. poae* fusadapamide BGC; chromosome statistics for Fp133; list of *F. poae* isolates and PCR results; PCR primer sequences; transcript expression of FDA BGC genes; BLASTp hits comparing Fda1 to the MIBiG database; fungal NRPS BGCs included in C-domain phylogenetic analysis; BLASTp hits comparing Fda3 and Fda4 to GenBank; Fp133 *N*-acetyltransferases and expression in two media (PDF)
Untrimmed alignment of all fungal condensation and epimerization domain protein sequences (FASTA file) (ZIP)

## References

[1] Witte, T. E. ; Overy, D. P. Untargeted metabolomic profiling of fungal species populations. In Proteomics in Systems Biology: Methods and Protocols; Geddes-McAlister, J. , Ed.; Springer Nature, 2022; Vol. 2456. 10.1007/978-1-0716-2124-0_24 35612754

[2] Witte T. E., Harris L. J., Nguyen H. D. T., Hermans A., Johnston A., Sproule A., Dettman J. R., Boddy C. N., Overy D. P. (2021). Apicidin
biosynthesis is linked to accessory chromosomes in *Fusarium
poae* isolates. BMC Genomics.

[3] Stenglein S. A. (2009). *Fusarium poae*: A
pathogen that needs more attention. Journal
of Plant Pathology.

[4] Thrane U., Adler A., Clasen P. E., Galvano F., Langseth W., Lew H., Logrieco A., Nielsen K. F., Ritieni A. (2004). Diversity in metabolite
production by *Fusarium langsethiae*, *Fusarium
poae*, and *Fusarium sporotrichioides*. Int. J. Food Microbiol..

[5] Vanheule A., Audenaert K., Warris S., van de Geest H., Schijlen E., Höfte M., De Saeger S., Haesaert G., Waalwijk C., van der
Lee T. (2016). Living apart
together: crosstalk between the core and supernumerary genomes in
a fungal plant pathogen. BMC Genomics.

[6] Tsuge T., Harimoto Y., Akimitsu K., Ohtani K., Kodama M., Akagi Y., Egusa M., Yamamoto M., Otani H. (2013). Host-selective
toxins produced by the plant pathogenic fungus *Alternaria
alternata*. FEMS Microbiology Reviews.

[7] Witte T. E., Villeneuve N., Boddy C. N., Overy D. P. (2021). Accessory chromosome-acquired
secondary metabolism in plant pathogenic fungi: the evolution of biotrophs
into host-specific pathogens. Frontiers in Microbiology.

[8] Hoogendoorn K., Barra L., Waalwijk C., Dickschat J. S., van der Lee T. A. J., Medema M. H. (2018). Evolution and diversity
of biosynthetic
gene clusters in *Fusarium*. Frontiers in Microbiology.

[9] Kobylarz M. J., Grigg J. C., Takayama S. J., Rai D. K., Heinrichs D. E., Murphy M. E. P. (2014). Synthesis of L-2,3-diaminopropionic
acid, a siderophore
and antibiotic precursor. Chemistry & Biology.

[10] Thomas M. G., Chan Y. A., Ozanick S. G. (2003). Deciphering
tuberactinomycin biosynthesis:
isolation, sequencing, and annotation of the viomycin biosynthetic
gene cluster. Antimicrob. Agents Chemother..

[11] Kuo Y.-H., Khan K. J., Lambein F. (1994). Biosynthesis
of the neurotoxin β-odap
in developing pods of *Lathyrus sativus*. Phytochemistry.

[12] Witte T. E., Hicks C., Hermans A., Shields S., Overy D. P. (2024). Debunking
the myth of *Fusarium poae* T-2/HT-2 toxin production. J. Agric. Food Chem..

[13] Nord C., Bjerketorp J., Levenfors J. J., Cao S., Strömstedt A. A., Guss B., Larsson R., Hughes D., Öberg B., Broberg A. (2020). Isopedopeptins A–H: cationic cyclic lipodepsipeptides
from *Pedobacter cryoconitis* up508 targeting WHO top-priority
carbapenem-resistant bacteria. ACS Chem. Biol..

[14] Kjær A., Larsen P. O., Sillén L. G., Andersson G., Stenhagen E., Palmstierna H. (1959). Amino acid
studies. part ii. structure
and synthesis of albizziine (l-2-amino-3-ureidopropionic acid), an
amino acid from higher plants. Acta Chem. Scand..

[15] Hansen F. T., Gardiner D. M., Lysøe E., Fuertes P. R., Tudzynski B., Wiemann P., Sondergaard T. E., Giese H., Brodersen D. E., Sørensen J. L. (2015). An update
to polyketide synthase and non-ribosomal
synthetase genes and nomenclature in *Fusarium*. Fungal Genetics and Biology.

[16] Labby K. J., Watsula S. G., Garneau-Tsodikova S. (2015). Interrupted
adenylation domains:
unique bifunctional enzymes involved in nonribosomal peptide biosynthesis. Natural Product Reports.

[17] Wang B., Kang Q., Lu Y., Bai L., Wang C. (2012). Unveiling
the biosynthetic puzzle of destruxins in *Metarhizium* species. Proc. Natl. Acad. Sci. U. S. A..

[18] Gao X., Haynes S. W., Ames B. D., Wang P., Vien L. P., Walsh C. T., Tang Y. (2012). Cyclization
of fungal nonribosomal
peptides by a terminal condensation-like domain. Nat. Chem. Biol..

[19] Rausch C., Hoof I., Weber T., Wohlleben W., Huson D. H. (2007). Phylogenetic analysis of condensation domains in NRPS
sheds light on their functional evolution. BMC
Evolutionary Biology.

[20] Wheadon M. J., Townsend C. A. (2021). Evolutionary and
functional analysis of an NRPS condensation
domain integrates β-lactam, d-amino acid, and dehydroamino
acid synthesis. Proc. Natl. Acad. Sci. U. S.
A..

[21] He R., Zhang J., Shao Y., Gu S., Song C., Qian L., Yin W.-B., Li Z. (2023). Knowledge-guided
data
mining on the standardized architecture of NRPS: Subtypes, novel motifs,
and sequence entanglements. PLOS Computational
Biology.

[22] Rao S. L. N., Adiga P. R., Sarma P. S. (1964). The isolation and
characterization
of β-N-oxalyl-L-α,β-diaminopropionic acid: a neurotoxin
from the seeds of *Lathyrus sativus*. Biochemistry.

[23] Silo-Suh L. A., Lethbridge B. J., Raffel S. J., He H., Clardy J., Handelsman J. (1994). Biological activities of two fungistatic antibiotics
produced by *Bacillus cereus* UW85. Appl. Environ. Microbiol..

[24] Hecht S. M. (2000). Bleomycin:
new perspectives on the mechanism of action. J. Nat. Prod..

[25] Li Y., Lee S. R., Han E. J., Seyedsayamdost M. R. (2022). Momomycin,
an antiproliferative cryptic metabolite from the oxytetracycline producer *Streptomyces rimosus*. Angew. Chem..

[26] Ermolenko D. N., Spiegel P. C., Majumdar Z. K., Hickerson R. P., Clegg R. M., Noller H. F. (2007). The antibiotic viomycin
traps the
ribosome in an intermediate state of translocation. Nature Structural and Molecular Biology.

[27] Wang M., Gould S. J. (1993). Biosynthesis of
capreomycin. 2. Incorporation of L-serine,
L-alanine, and L-2,3-diaminopropionic acid. J. Org. Chem..

[28] Drechsel H., Freund S., Nicholson G., Haag H., Jung O., Zähner H., Jung G. (1993). Purification and chemical characterization
of staphyloferrin B, a hydrophilic siderophore from staphylococci. Biometals.

[29] Ikegami F., Ongena G., Sakai R., Itagaki S., Kobori M., Ishikawa T., Kuo Y.-H., Lambein F., Murakoshi I. (1993). Biosynthesis
of β-(isoxazolin-5-on-2-yl)-l-alanine by cysteine synthase in *Lathyrus sativus*. Phytochemistry.

[30] Guo Q., Wu D., Gao L., Bai Y., Liu Y., Guo N., Du X., Yang J., Wang X., Lei X. (2020). Identification of the
AMA synthase from the aspergillomarasmine A biosynthesis and evaluation
of its biocatalytic potential. ACS Catal..

[31] Bach E., Christensen S., Dalgaard L., Larsen P. O., Olsen C. E., Smedegård-Petersen V. (1979). Structures,
properties and relationship
to the aspergillomarasmines of toxins produced by *Pyrenophora
teres*. Physiological Plant Pathology.

[32] Ballio A., Bottalico A., Buonocore V., Carilli A., Di Vittorio V., Graniti A. (1969). Production and isolation of aspergillomarasmin B (lycomarasmic
acid) from cultures of *Colletotrichum gloeosporioides* Penz. (Gloeosporium olivarum Aim.). Phytopathologia
Mediterranea.

[33] Haenni A.
L., Robert M., Vetter W., Roux L., Barbier M., Lederer E. (1965). Structure
chimique des aspergillomarasmines A et B. Helv.
Chim. Acta.

[34] Plattner Pl.A., Clauson-Kaas N. (1945). Über ein Welke erzeugendes Stoffwechselprodukt
von *Fusarium lycopersici* Sacc. Helv. Chim. Acta.

[35] Müller S., Rachid S., Hoffmann T., Surup F., Volz C., Zaburannyi N., Müller R. (2014). Biosynthesis of crocacin involves
an unusual hydrolytic release domain showing similarity to condensation
domains. Chem. Biol..

[36] Hai Y., Jenner M., Tang Y. (2019). Complete stereoinversion
of L-tryptophan
by a fungal single-module nonribosomal peptide synthetase. J. Am. Chem. Soc..

[37] Cuomo C. A., Güldener U., Xu J. R., Trail F., Turgeon B. G., Di Pietro A., Walton J. D., Ma L. J., Baker S. E., Rep M. (2007). The *Fusarium graminearum* genome reveals
a link between localized polymorphism and pathogen specialization. Science.

[38] Selker E. U., Garrett P. W. (1988). DNA sequence duplications trigger gene inactivation
in *Neurospora crassa*. Proc.
Natl. Acad. Sci. U.S.A..

[39] Xue A. G., Chen Y., Seifert K., Guo W., Blackwell B. A., Harris L. J., Overy D. P. (2019). Prevalence of Fusarium species causing
head blight of spring wheat, barley and oat in Ontario during 2001–2017. Canadian Journal of Plant Pathology.

[40] Islam M. N., Tabassum M., Banik M., Daayf F., Dilantha
Fernando W. G., Harris L. J., Sura S., Wang X. (2021). Naturally
occurring *Fusarium* species and mycotoxins in oat
grains from Manitoba, Canada. Toxins.

[41] Pluskal T., Castillo S., Villar-Briones A., Orešič M. (2010). MZmine 2:
Modular framework for processing, visualizing, and analyzing mass
spectrometry-based molecular profile data. BMC
Bioinformatics.

[42] Schmid R., Petras D., Nothias L. F., Wang M., Aron A. T., Jagels A., Tsugawa H., Rainer J., Garcia-Aloy M., Dührkop K. (2020). Ion identity molecular networking in the GNPS
environment. bioRxiv.

[43] Wang M., Carver J. J., Phelan V. V., Sanchez L. M., Garg N., Peng Y., Nguyen D. D., Watrous J., Kapono C. A., Luzzatto-Knaan T. (2016). Sharing and community
curation of mass spectrometry
data with Global Natural Products Social Molecular Networking. Nat. Biotechnol..

[44] Dührkop K., Fleischauer M., Ludwig M., Aksenov A. A., Melnik A. V., Meusel M., Dorrestein P. C., Rousu J., Böcker S. (2019). SIRIUS 4:
a rapid tool for turning tandem mass spectra into metabolite structure
information. Nat. Methods.

[45] Gu Z., Eils R., Schlesner M. (2016). Complex heatmaps
reveal patterns
and correlations in multidimensional genomic data. Bioinformatics.

[46] Koren S., Walenz B. P., Berlin K., Miller J. R., Bergman N. H., Phillippy A. M. (2017). Canu: Scalable and accurate long-read
assembly via
adaptive κ-mer weighting and repeat separation. Genome Res..

[47] Walker B. J., Abeel T., Shea T., Priest M., Abouelliel A., Sakthikumar S., Cuomo C. A., Zeng Q., Wortman J., Young S. K. (2014). Pilon: an integrated tool for comprehensive
microbial variant detection and genome assembly improvement. PLoS One.

[48] Kolmogorov M., Yuan J., Lin Y., Pevzner P. A. (2019). Assembly
of long,
error-prone reads using repeat graphs. Nat.
Biotechnol..

[49] Li H. (2018). Minimap2:
Pairwise alignment for nucleotide sequences. Bioinformatics.

[50] Flynn J. M., Hubley R., Goubert C., Rosen J., Clark A. G., Feschotte C., Smit A. F. (2020). RepeatModeler2 for
automated genomic
discovery of transposable element families. Proc. Natl. Acad. Sci. U. S. A..

[51] King R., Urban M., Hammond-Kosack M. C.
U., Hassani-Pak K., Hammond-Kosack K. E. (2015). The completed genome sequence of the pathogenic ascomycete
fungus *Fusarium graminearum*. BMC Genomics.

[52] Rozas J., Ferrer-Mata A., Sánchez-DelBarrio J. C., Guirao-Rico S., Librado P., Ramos-Onsins S. E., Sánchez-Gracia A. (2017). DnaSP 6: DNA
sequence polymorphism analysis of large data sets. Mol. Biol. Evol..

[53] Terlouw B. R., Blin K., Navarro-Muñoz J. C., Avalon N. E., Chevrette M. G., Egbert S., Lee S., Meijer D., Recchia M. J. J., Reitz Z. L. (2023). MIBiG 3.0: a community-driven
effort to annotate experimentally validated biosynthetic gene clusters. Nucleic Acids Res..

[54] Hicks C., Witte T. E., Sproule A., Hermans A., Shields S. W., Colquhoun R., Blackman C., Boddy C. N., Subramaniam R., Overy D. P. (2023). CRISPR-Cas9 gene editing and secondary metabolite screening
confirm *Fusarium graminearum* C16 biosynthetic gene
cluster products as decalin-containing diterpenoid pyrones. Journal of Fungi.

