# Supplementary material for: Discovery of Fusadapamides, Accessory Chromosome-Associated Metabolites Incorporating l‑2,3-Diaminopropionic Acid in Fusarium poae
Source: J Nat Prod. 2025 Dec 10;88(12):2883–96. doi: 10.1021/acs.jnatprod.5c01104 (PMC12751119; doi:10.1021/acs.jnatprod.5c01104)
Supplement: Supplementary file 1 [file np5c01104_si_001.pdf]

Supporting Information

Discovery of fusadapamides, accessory chromosome-associated metabolites incorporating L-2,3-diaminopropionic acid in *Fusarium poae*

Thomas E. Witte[1, 2], Linda J. Harris[1], Luke Albert Paquette[2], Anne Hermans[1], Amanda Sproule[1], Anne Johnston[1], Jason Ma[2], Michael G. Darnowski[2], Whynn Bosnich[1], Danielle Schneiderman[1], Xiben Wang[3], Benjamin A. G. Beavington[2], Izhar U. H. Khan[1], Christopher N. Boddy[2*], David P. Overy[1*]

[1]Agriculture and Agri-Food Canada, Ottawa Research and Development Centre, Ottawa, Canada.

[2]Department of Chemistry and Biomolecular Sciences, University of Ottawa, Ottawa, Canada.

[3]Agriculture and Agri-Food Canada, Morden Research and Development Centre, Morden, Canada.

* Corresponding authors

E-mail: Christopher.boddy@uottawa.ca (CNB), E-mail: david.overy@agr.gc.ca (DPO)

Table of Contents

Supporting Information

Supporting Info 1	Materials and methods details	3-12
Supporting Info 2	Newick tree for condensation domains	13-15

Supporting Figures

Figures S1 to S7	NMR spectra of fusadapamide A	16-22
Figures S8 to S10	NMR spectra of fusadapamide B	23-25
Figures S11 to S12	NMR spectra of fusadapamide C	26-27
Figure S13	MS/MS fragmentation analysis of fusadapamide A	28
Figures S14-S17	NMR spectra of compound 4 (α-N-dimethyl Dap)	29-33
Figures S18-S21	NMR spectra of compound 5 (β-N-dimethyl Dap)	34-38
Figure S22	Marfey's analysis of fusadapamides	39
Figure S23	CHEF gel confirmation of Fp133 small chromosome sizes	40
Figure S24	PCR primer design strategy	54
Figures S25 to S31	PCR/Southern blot validation of FDA BGC deletion mutants	55-60
Figure S32	Comparison of *fda1* modules 1 and 2	64
Figure S33-S34	Fda4 expression and in vitro Nitrogen source characterization	65-66
Figure S35	Oat groat mass and discoloration after *F. poae* infection	67
Figure S36	Synteny analysis of *F. poae* fusadapamide BGC	68

Supporting Tables

Table S1	Chromosome statistics for *Fp*133	41
Table S2	List of *F. poae* isolates and PCR results	42-51
Table S3	PCR primer sequences	52
Table S4	Transcript expression of FDA BGC genes	53
Table S5	BLASTp hits comparing Fda1 to MIBIG database	61
Table S6	Fungal NRPS BGCs included in C-domain phylogenetic analysis	62
Table S7	BLASTp hits comparing Fda3 and Fda4 to genbank	63
Table S8	*Fp*133 N-acetyltransferases and their expression in two media	69-70

Supporting Information 1: Materials and Methods details

Fp133 protoplast generation and karyotyping details

To generate protoplasts, conidial stocks were thawed and immediately inoculated into 100 mL of potato dextrose broth (PDB, BD Difco Brand, NJ, USA) and incubated for 13 hours at 25°C, shaken at 100 rpm in the dark. At this point, conidial germlings were inspected using a compound microscope to estimate their growth stage, ensuring they are freshly germinated and have not formed confluent mycelial masses. The germlings were then filtered from the broth using a doubled layer of miracloth, and washed once with 15 mL of sterile water and twice more with 30 mL of 1.2M KCl. The washed germlings were scraped from the miracloth and added to a lysing enzyme solution consisting of 300 mg *Trichoderma harzianum* lysing enzymes (Sigma Aldrich, Missouri), 400 mg yatalase (Takara Bio, USA) and 150 mg driselase (Sigma Aldrich, Missouri) added to 20mL of 1.2M KCl. After approximately 1 hour and 45 minutes of incubation at 30°C in the dark under very gentle rotation at 80 rpm, the germling/enzyme solution was diluted with 20 mL cold 1.2 M KCl, gently mixed, and then filtered through a 40 micron mesh into a sterile falcon tube. The filter was washed with an additional 10 mL cold KCl. The solution was then centrifuged at 5000 rpm for 10 minutes at 4°C. The supernatant was discarded and the pellet resuspended in 20 mL of cold STC buffer. The pellet was resuspended in 20 mL of cold SE buffer, then centrifuged again and resuspended in approximately 2 mL of SE buffer. 100 µL agarose plugs were then made, each containing approximately $1X10^9$ protoplasts suspended in a final concentration of 0.5% low-melt agarose containing proteinase K (BioShop Canada Inc). Plugs were then incubated for 48 hours at 50°C in the dark, each immersed 2 mL of ET buffer containing 0.5 mg/mL proteinase K and 1% (w/v) N-lauroyl-sarcosine (Sigma Aldrich). Finally, plugs were washed three times with 50 mM EDTA with a 1 hour incubation at room temperature between each wash, and occasional agitation by tube inversion. Plugs were then stored in ET buffer at 4°C until ready for use.

Electrophoretic karyotyping was performed using a CHEF DRII system (BioRad, QC) using the following settings: 3.0 V/cm, 50-hour runtime, 250-900 s switch time gradient. Plugs were embedded into 0.8% megabase agarose (BioRad, Spain) dissolved in TAE buffer. TAE was used as the buffer during the CHEF DRII operation, chilled at 14°C for the length of the run. Gels were then stained using ethidium bromide, and visualized using a Gel Doc (BioRad, QC). Chromosome sizes were inferred by comparison to *Saccharomyces cerevisiae* and *Hansenula wingeii* chromosomal "ladders" (BioRad, QC).

Transposable element prediction, repeat content masking and repeat-induced point mutation (RIP) analysis

RepeatModeler v2.0.1 [1] was used to generate a *de novo* library of repeated sequences for *Fp*133. TEs were annotated using the RepBase 2018 library merged with the *F. poae* 2516 library published in Vanheule et al. 2016.[2] RepeatMasker v4.2.1-p1 was then used to softmask the assemblies in preparation for gene annotation. Repeat element content per chromosome was calculated based on the percentage of base pairs masked by RepeatMasker using the custom repeat element library. To calculate the percentage of RIP-affected regions per chromosome, the RIPper online portal [3] was used (accessed August 29, 2024) with default parameters, and the *Fp*133 genome was submitted with predicted centromeres and telomeres removed manually.

Gene and centromere prediction in Fp133 and syntenic comparison to Fp157

The *Fp*133 repeat-masked assembly was annotated using v1.8.14 of the Funannotate pipeline [4], using the RNAseq transcript evidence generated in this study to assist in gene prediction. Transposable elements were filtered from the final gene models based on BLASTn similarity to previously annotated TEs from *F. poae* strain 2516 [2] merged with a small database of fungal TEs built into Funannotate. The *Fp*133 genome was then passed to Antismash v7.0.0 [5] for biosynthetic gene cluster prediction using fungal parameters. BGC chromosome location outputs (Figure 3A) were adapted from antiSMASH v7.0.0 graphical output to build chromosome diagrams in post-processing graphics design software (Affinity Designer). Genes were further annotated using SignalP6,[6] Interproscan v5.52-86.0,[7] Eggnog-mapper v2.1.9,[8] Diamond BLASTp search of the UniProt DB version 2022_04, and Phobius.[9] Annotations were generated and merged using Funannotate *annotate* using default parameters. Manual annotation corrections were made to the final gene names, including renaming the APS genes, as well as generic

Fusarium PKS and NRPS numbered genes to match those in the *Fusarium* literature.[10,11] Isocyanide synthase clusters were annotated based on the gene cluster family assignments of Nickles et al. (2023).[12] Terpene synthases were annotated based on nomenclature applied to *F. langsethiae* and *F. fujikuroi*.[13,14]

Centromeres were predicted manually, based on the detection of low GC areas (<15% GC content) spanning at least 39K bases. Core chromosome centromere predictions were compared to equivalent centromere locations and lengths in *F. graminearum*.[15] Collinearity between *Fp*157 and *Fp*133 was mapped with MCScanX, using Blastp-derived pairwise homology between the two proteomes as input, and visualized using Synvisio.[16] The APS regions of Fp133 and Fp157 were compared using Blastn and visualizations were adapted from EasyFig v2.2.5 output (Figure 3C).

Fp133 RNA isolation and sequencing

*Fp*133 was cultured on two solid agar media types, MMK2 and YES, in the dark at 25 C. RNA was harvested from the mycelium at days 2 and 6 after inoculation using a Qiagen RNeasy Plant Mini kit followed by a Qiagen DNAse treatment, according to manufacturers instructions. RNA concentration and quality was verified using a QuickDrop spectrophotometer (Molecular Devices) and a 2100 BioAnalyzer (Agilent Technologies). RNAseq libraries were prepared using the Illumina NextSeq 500/550 High Output v2 kit, according to manufacturers instructions, and the RNA sequencing was conducted as 150 nucleotide single reads.

N-acetyltransferase homolog search

Putative acetyltransferases which could be compensating for the loss of *fda5* were identified by scanning the predicted Fp133 proteome against HMMs corresponding to Pfam domains PF00583 (Acetyltransf_1), PF13508 (Acetyltransf_7), and PF13673 (Acetyltransf_10) using HMMER v3.3.2. Searches were conducted with hmmscan using default parameters and model-specific gathering thresholds (--cut_ga). Hits with domain-level E-values ≤ 0.01 and query coverage ≥60 amino acids were retained. *Fda5* (*FPOAC2_13306*) matched primarily to the Acetyltransf_10 model but also exhibited weaker homology to Acetyltransf_7 and Acetyltransf_1, consistent with its predicted GNAT-type fold.

The resulting list of 51 GNAT-domain proteins (**Table S8**) was filtered to remove sequences with annotations corresponding to housekeeping NAT/HAT enzymes, including *GCN5*, *NAA20*, *NAA30*, *SPT10*, *ATS1*, *GNA1*, *ARD1*, *ELP3*, *HPA3*, and *ESA1*. Remaining candidates were cross-referenced with RNA-seq–derived gene expression data produced in this study. Transcripts with transcripts per million (TPM) >10 on average across conditions were considered expressed.

Fungal C-domain alignment and sequence analysis details

Fungal C- and E-domain sequences sourced from the MIBIG v3.0 database were aligned using MAFFT v7.457 (2020/Nov/23) [17] using automated strategy finding (-auto) parameters and default penalties. The alignment was manually trimmed at both ends and then a second, automated trimming was performed using trimal v1.4.rev15 [18] using the “-gt 0.5 -cons 60” flags, which removes any site with >= 50% gaps while preserving at least 60% of the original alignment. The trimmed alignment consisted of 662 amino acid sites, of which 621 were parsimony informative. A fasta file of the trimmed aligned sequences can be found in **Supplemental data file “198_CE_alignment.fasta”** (not included in thesis submission). IQTree2 v2.1.2 [19] was then used to build consensus and maximum-likelihood trees, with automated model finding supplying LG+F+R9 as the best substitution model based on Bayesian information criteria (BIC) [20]. SH-aLRT with 1000 bootstrap replicates and Ultrafast bootstrapping [21] with 1000 replicates were both used to assign confidence values to the nodes. The Newick format of the ML tree can be found in **Supplemental Info 3** with boot strap values labeled as SH-aLRT:Ultrafast after each node (eg 89.7:95). The maximum likelihood tree was then visualized with SH-aLRT bootstrap supports at key nodes using the interactive Tree of Life (iTOL) web-based server.

CRISPR-mediated gene deletion protocol details

Design of crRNA

crRNAs for all genes were designed using Genenious Software (version 2022.1.1). Parameters were set to search for presence of Protospacer-Adjacent Motifs (PAMs) with the sequence NGG, where N was any nucleotide. Sequence length was 20bp and off target sites were determined by blasting against whole genome sequence of *Fusarium poae* (Fp133) in Geneious Software.

crRNA sequences for 5' and 3' of each gene

Gene	crRNA
FPOAC2_13301	TCAAGCGACAACGATATGCC
	TAGAGTTTGCGGTTATTGGG
FPOAC2_13302	TGATTTAGCGCCAGACACAA
	GAGATACATCCTGATGCCTC
FPOAC2_13303	AAAAATCGAATTTTAACACT
	TAACGCTCCAGCTCCATTCG
FPOAC2_13305	AATTCCTCAATGTGTATAGT
	ATCAAGCCACCGATACAGAG
	TGATTTAGCGCCAGACACAA
FPOAC2_13306	ACGTACATGAGCAACAGTCA
	AACTCGACAATTCTCTCCAG
APS1 FPOAC2_13359	AATGGCAAACCAACAAACTT
	TCGGCTGAGAATGTTCCAGG

Construction of donor DNA for target gene replacement

The Homologous directed repair (HDR) templates encompassed the pTrpC promoter (*Aspergillus nidulans*) fused to the hygromycin resistance gene flanked by 35-50 bp of homologous sequence upstream and downstream of the target gene. Primers were designed by including 35-50 bp of DNA upstream and downstream of 5' region and 3' region of gRNA cut sites. The 3' region of both the forward and reverse primers were fused to 5' TCGACAGAAGATGATATTG 3' (binds to promoter pTrpC) and 5'CTATTCCTTTGCCCTCGGACGA3'(Hygromicin resistance gene stop site) respectively. For APS1, the HDR template encompassed the pTrpC promoter fused to the geneticin resistance gene (neo). The 3' region of both the forward and reverse primers were fused to 5' TCGACAGAAGATGATATTG 3' (binds to promoter pTrpC) and 5' CTCAGAAGAACTCGTCAAGA 3'(geneticin resistance gene stop site) respectively. The pRF-HU2 vector (ref) and pRF-GU2 (ref) (30ng each) was used as templates for amplification of HDR templates for the hygromycin and geneticin resistance genes respectively. PCR amplification of HDR templates was done using Advantage Taq Polymerase (Takara Bio) as per manufacturer's instructions. Purification of PCR product was done using the GenepHlow Gel/PCR kit (FroggaBio) as per manufacturer's instructions. All HDR products were sequenced to confirm the absence of any PCR errors.

crRNA and HDR primers sequences for each gene

Gene	crRNA	HDR primers sequence (homologous to Hygromycin R)

FPOAC2_ 13301	TCAAGCGACAAC GATATGCC	ACGGAGCCCCTGCACTGCCTATTTCAAGCGACAACGATATTCG ACAGAAGATGATATTG
	TAGAGTTTGCGG TTATTGGG	TATTAGTAAGACGGGGTTAGCAACTAGAGTTTGCGGTTATTCT ATTCCTTTGCCCTCGGACGA
FPOAC2_ 13302	TGATTTAGCGCC AGACACAA	CTCATTGTTCATTTGCAACCGTTCTGATTTAGCGCCAGACATC GACAGAAGATGATATTG
	GAGATACATCCT GATGCCTC	TGCAGCTGACGTTTCTAGTCAGAGATACATCCTGATGCCTATT CCTTTGCCCTCGGACGA
FPOAC2_ 13303	AAAAATCGAATTT TAACACT	CGCCCTGATCGGCTTTAAGATGGAAAAATCGAATTTTAACTCG ACAGAAGATGATATTG
	TAACGCTCCAGC TCCATTCG	ATGGTAGATGAACTTATCCTGGTAACGCTCCAGCTCCATCTAT TCCTTTGCCCTCGGACGA
FPOAC2_ 13305	AATTCCTCAATGT GTATAGT	TCCTTGTTATCATGGCAAAAACCGAATTCCTCAATGTGTATTCG ACAGAAGATGATATTG
	ATCAAGCCACCG ATACAGAG	TAGAGCCCTAACTACTGCCTGACATCAAGCCACCGATACACTA TTCCTTTGCCCTCGGACGA
FPOAC2_ 13306	ACGTACATGAGC AACAGTCA	CGAATTGAGATCCAGTTTTGAGAACGTACATGAGCAACAGTCG ACAGAAGATGATATTG
	AACTCGACAATTC TCTCCAG	GTACGTTACGGAATATATATAAACTCGACAATTCTCTCCACTAT TCCTTTGCCCTCGGACGA
FPOAC2_ 13359	AATGGCAAACCA ACAAACTT	TTATCATTTGTTCATATTATCAAAATGGCAAACCAACAAATCGA CAGAAGATGATATTG
	TCGGCTGAGAAT GTTCCAGG	GTTATGTTATCCTTCTCTGACACAATTGAGCACTCGGCTGAGA ATGTTCCTCAGAAGAACTCGTCAAGA

Crispr mediated gene disruption

gRNA duplexes were constructed by combining 0.5 µmoles of a crRNA and tracrRNA (IDT) in Nuclease free duplex buffer (IDT) for a final concentration 33 µM in separate microfuge tubes for each crRNA. The mixtures were heated 95ºC for 5 min, followed by cooling at room temperature (22-25ºC) for 15 mins. The duplex mixtures (0.05 µM) were incubated separately for 10 min at RT with 0.75 ug of Cas9 enzyme (IDT) in 20 mM HEPES, 150 mM KCl, pH 7.5 in a final volume of 13.25 µL. The duplex mixtures for 5' and 3' region (RNP complex) of the gene were combined and incubated for a further 5 min. The RNP complex was combined with *F. paoe* 133 protoplast (200 µl, 10^8 /mL), 6-8 µg of the purified HDR template in 25 µL of PEG (4000)- $CaCl_2$ buffer- 60% w/v PEG 4000, 50 mM $CaCl_2 \cdot H_2O$, 450 mM Tris-HCl, pH 7.5 and incubated for 1 hour on ice. Following the incubation, add 1.5 mL of PEG (4000)-$CaCl_2$ buffer, and incubate at RT for 20 minutes. After incubation, total volume was brought to 2 mL by addition of STC (1.2 M Sorbitol, 10 mM Tris-HCl (pH 8.0), 50 mM $CaCl_2$) buffer followed by addition of liquid TB3 media (0.3% yeast extract, 0.3% casamino acids, 20% sucrose) bringing total volume to 4-5 mL. The mixture was incubated in a rotary shaker for 18-20 hours at 25°C. A series of dilutions 300 µL, 500 µL, 800 µL and 1000 µL of regenerated fungal protoplasts were mixed with 20 mL of molten TB3 (45°C) containing 100 mg/L of hygromycin B or geneticin, poured in sterile petri plates and incubated at 25°C for 5-6 days for selection. Putative transformants were then transferred to PDA (potato dextrose agar) plates containing 150 mg/L of hygromycin B or geneticin and allowed to grow for 3-5 days at 25°C. Single spores were isolated by scraping with mycelia using a sterile inoculating loop and diluted in 500 µL and plated onto 2% agar plates. After 14 hr incubation at 25ºC, single spores were isolated using a microscope and transferred to PDA for 5-6 days.

To determine whether the gene deletion mutants produced fusadapamide during axenic culturing, agar plugs were inoculated from strains grown on SNA media into 15mL of YES broth in slant tubes. Growth, extraction and processing parameters were exactly as described as above for the untargeted metabolomics experiments. The metabolomics protocol was repeated for *ΔFDA3* and *ΔFDA4* on two media types (MMK2 and YES) in quintuplicate, to confirm of the low fusadapamide signals detected from those gene deletion mutants.

Cloning of fda4 into an expression vector (pLAP01).

The *fda4* gene was purchased from Thermo Fisher Scientific as a codon-optimized string assembly with no introns. The gene was amplified using 1 ng of *fda4*, 0.5 µM of both forward and reverse primers (*fda4f* and *fda4r*, respectively), 200 µM dNTPs, 1X Q5 High-Fidelity DNA polymerase buffer (NEB), and 1U Q5 High-Fidelity DNA polymerase (NEB). The optimal annealing temperature was determined to be 72˚C using the NEBTm calculator. Nonetheless, PCR amplification was performed using NEB's "PCR using Q5-High Fidelity DNA polymerase" protocol with a gradient of annealing temperature ranging from 62˚C to 72˚C. The resulting amplicons were gel purified and digested by endonucleases NheI and BamHI-HF for 4 hours at 37˚C using 1 ug DNA, 1X NEBuffer (NEB) 1.1, 20 U BamHI-HF (NEB), 10 U NheI (NEB). Digestion of the pET28a vector was completed using the same reagents and conditions. 2.5 U of recombinant shrimp alkaline phosphatase (NEB) was added to the vector digestion to avoid recircularization of the vector during ligation. Insert and linearized vector were gel purified (Omega Biotek EZNA cycle pure kit) following the manufacturer's instructions. Purified *fda4* was ligated into linear pET28a using a 3:1 ratio of amplicon to vector. The ligation was completed using 1X T4 DNA ligase buffer, 50 ng of digested pET28a, 43 ng digested *fda4* and 40 U T4 DNA ligase. The reaction was incubated at 16˚C for 16 hours and transformed into chemically competent XL1-Blue *E. coli*. Transformants were selected on LB agar plates supplemented with 50 ug/mL kanamycin. A single colony was inoculated into 10 mL of LB with 50 ug/mL of kanamycin. This culture was incubated at 37˚C overnight. The recombinant plasmid DNA (pLAP01) was isolated with the Omega Biotek EZNA plasmid DNA extraction kit using the manufacturer's protocol. The resultant plasmid, pLAP01, was confirmed via Sanger sequencing (Génome Québec).

fda4 synthetic gene sequence

atgggcagcagccatcatcatcatcatcacagcagcggcctggtgccgcgcggcagccatatggctagcatggccaaaaccgaatttctgaatgtt
tatagcggtccgcaggcactgcagcagtattttgatccggatgaacagccgctgatgccgctggttgaactgcctccgcgtctgaatacctttagcggt
gatggtgttcgtatttttgccaaaatgctgaccacactgcctgcacagaatgttaaagcactgccagcactgaatatgattcgtaatgcaccgggtagc
gaacgtaaaagcgttgttgaagcaagcagcggtagcaccgttattagcctggcactggcaagccgtattctgaatggtaatgatgatgttaccgccta
cgttaccaataaaaccgaaaaatcacgcctgcagaccctgagctttttcggtctgaaaattaacctgtatggtggtccgagccagccggaaattacc
gatgaacgtggtattatgcagaaaatcaaacgttgggctgaagaggatgatagcctgtgggcaccgagccagtatgaaaatgaaagcaattatcgt
agccatatgctgtggacaggtccgcagctgctgaaacagctgccgaatattaacatcttttgtgcaggtatgggtagcgcaggttgtgttaccggcac
cggtctgtatctgaaagaacataaaccggatgtgaaaattatcggcgtgtgtaatcagaaaggtgatccgattccgggtccgcgtccggcaagcctg
tttgaacatattagctttccgtggaaaaaggtggtggataccattgaagaagttgatagcgttcagagctatcgtctgagcatgcagctgagccgtgaa
ggtctgatttgcggtccgagcagtggtatggccctgcaagcactgatgaattatctgcagaaagcaaaagatgaaggctatctggcagaacatgcc
ggtgcagatggtcaggttcgtgcagtttttccgtgttgtgatctgccgtatcagtatatggataactacttcaaaaagctggaaccgagcgattttccgcct
gttgcaaattatcatctgctggatattgatacccacaactatgatagcacctgggaattagatgttcatgcagttctggatctgctgcagacaggcctgc
agccgaccgatgatatgggtgcaagcagcaaagttcaggttattgatattcgtgcaccggaagattttcatagcggcaccgcactgagcgccaaaa
atctgccgtttgcaagcgttaccagcaataccgcaagtccgtttgatgatgcagccgttctggaaagccagtggctggaaatgaaaacccgtgttattc
atgatgccgaagatgatttttccaacgatggtaaacaggtggtgattatttgctataatggtgaaagcgcacgtgttctgaccagcattctgcgtgcaca
tggtgttaaagcctatagcgttaaaggtggtattagctcactgtatcgttggctggatgttcgtcagtaa

Expression and purification of Fda4.

Chemically competent *E. coli* BL21 (λDE) cells was transformed with pLAP01 and plated onto LB agar plate supplemented with 50 µg/mL kanamycin and incubated at 37˚C overnight. A single colony was inoculated into 10 mL of LB supplemented with 50 ug/mL kanamycin and incubated overnight. 2 mL of the dense overnight culture (0.05% v/v) was added to 400 mL of LB and kanamycin. The culture was incubated at 37˚C at 200 rpm until an OD600 of 0.5 was reached. Gene expression was induced by adding 1 mM IPTG to the culture and incubated for 18 hours at room temperature and 200 rpm. The cells were pelleted by centrifugating for 20 minutes at 3 600 g and 4˚C. The pellet was resuspended in 10 mL

of cell lysis buffer (300 mM NaCl, 100 mM Tris, 10% v/v glycerol, 1 mg/mL lysozyme, 1 mg/mL pepstatin A, 1 mg/mL leupeptin, pH 7.4). The resuspended cells were lysed by sonication. The cell debris was pelleted by centrifuging for 1 hour at 12 000 g and 4˚C. The lysate (supernatant) was decanted into a pre-chilled falcon tube. The lysate was incubated with 400 µL of Ni-NTA resin (Qiagen) for 1.5 hours at 4˚C with constant shaking on an orbital shaker. The resin was placed into a column and flowthrough was then collected into a clean chilled falcon tube. The resin was washed with 10 collum bed volumes (CBV) of 0 mM imidazole (100 mM Tris, 300 mM NaCl, X mM imidazole, pH 7.4). The resin was washed a second time with 10 CBV of 20 mM imidazole. Fda4 was eluted from the resin using two 5 CBV elutions of 100 mM imidazole and two 5 CBV elutions 250 mM imidazole. The 100mM and 250 mM elutions were combined and concentrated and buffere exchanged into 50 mM phosphate buffer (pH 7.4) by ultrafiltration using a Vivaspin20 Centrifugal Concentrator (10 kDa MWCO PES, Sartorius). Fda4 concentration was then determined via Bradford Assay.

Fda4 substrate specificity assay experimental methods details

Enzyme assays were performed in 50 mM phosphate buffer pH 7.4, 100 mM KCl, 5 mM DTT, 100 µM PLP, 10 µM Fda4, and 10 mM of L-Ser, O-phospho Ser, or O-Acetyl Ser and 10 mM of the nitrogen nucleophile (either L-Ala, D-Ala, L-Glu or L-Asp). The reactions were incubated overnight at room temperature. Fmoc-Cl derivatization quenched the reactions which were analyzed by LCMS. Extracted ion chromatograms were extracted for the expected products.

Fmoc-Cl derivatization and LCMS analysis of in vitro assays

Reactions were quenched using 1V (assay volume) of 15 mg/mL Fmoc-Cl in acetone. These were mixed and left to incubate at room temperature for 10 minutes. Excess Fmoc-Cl and its hydrolysis product Fmoc-OH was washed out of solution by liquid-liquid extraction using pentane. Acetonitrile (0.5V) was added and mixed before being injected into the LCMS for analysis.

10 uL samples were injected and analyzed on a Shimadzu Prominence UFLC coupled to a Shimadzu LCMS-2020 equipped with a Shimadzu SPD-20A/SPD-20AV UV-VIS detector. An Agilent Eclipse plus C18 column (150 mm x 4.6 mm, 3.5 um) was used for chromatographic seperation with a flow rate of 1 mL/min, running a gradient of water (0.05% formic acid) and acetonitrile (0.05% formic acid) starting at 5% acetonitrile for 2 mins, increasing to 99% acetonitrile by 8 mins, held at 99% acetonitrile until 12 mins, returning to 5% acetonitrile by 13 mins and held at 5% acetonitrile until 15 mins. The LCMS was operated in ESI+ mode, monitoring the range of 250-550 *m*/*z*.

Synthesis of dimethylated L-Dap standards for Marfey's analysis

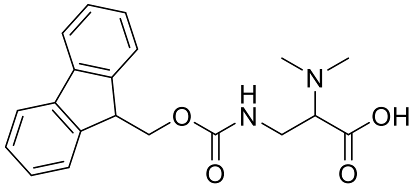

4: α-N-dimethyl-Dap (Fmoc protected**)**

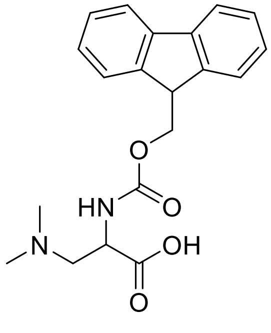

5: β-N-dimethyl-Dap (Fmoc protected)

Compound 4: $(CH_3)_2$-DAP(Fmoc)-OH = α-N-dimethyl-Dap (Fmoc protected)

Boc-DAP(Fmoc)-OH (100 mg, 0.49 mmol, 1 eq) was dissolved in 5 mL TFA:H_2O (1:1) and the solution was stirred at room temperature for 2 h or until the reaction was judged to be complete by TLC. The mixture was dried *in vacuo* and used directly without further purification. At 0 °C, the resulting mixture was dissolved in EtOH (0.1 M). Formaldehyde (110 uL, 1.47 mmol, 37% aqueous solution) was added to the mixture, and allowed to stir for 10 minutes, then $NaBH_3CN$ (123 mg, 1.96 mmol, 4 eq) was added to the

mixture and allowed to stir at rt overnight. The solvent was removed *in vacuo*. The crude residue was purified by reverse-phase HPLC (5%– 95% ACN/H_2O +0.1% TFA) and then lyophilized to afford **(4)** as a colorless solid.

Compound 5: Fmoc-DAP$(CH_3)_2$-OH = β-N-dimethyl-Dap (Fmoc protected)

Fmoc-DAP(Boc)-OH (100 mg, 0.49 mmol, 1 eq) was dissolved in 5 mL TFA:H_2O (1:1) and the solution was stirred at room temperature for 2 h or until the reaction was judged to be complete by TLC. The mixture was dried *in vacuo* and used directly without further purification. At 0 °C, the resulting mixture was dissolved in EtOH (0.1 M). Formaldehyde (110 uL, 1.47 mmol, 37% aqueous solution) was added to the mixture, and allowed to stir for 10 minutes, then $NaBH_3CN$ (123 mg, 1.96 mmol, 4 eq) was added to the mixture and allowed to stir at rt overnight. The solvent was removed *in vacuo*. The crude residue was purified by reverse-phase HPLC (5%– 95% ACN/H_2O +0.1% TFA) and then lyophilized to afford **(5)** as a colorless solid.

Marfey's method for residue stereochemistry assignments

Full hydrolysis was performed on 0.25 mg of each purified compound, dissolved in 100 µL of 6 M HCl made with 1:1 D_2O:H_2O and shaken in a sealed tube on a thermomixer at 100 °C for 24 hours. The hydrolyzed samples were dried under N_2. Samples were derivatized by the addition of 20 µL of 1 M $NaHCO_3$ and 380 µL of L-FDAA stock solution (6 mg in 5.7 mL acetone) followed by incubating at 40 °C for 1 hour. The reactions were cooled to room temperature and then neutralized with 20 µL of 1 M HCl. The samples were then filtered through 0.2 µM PTFE syringe filters into fresh vials, and injected into the UPLC-HRMS using a gradient starting at 5 % acetonitrile with 0.1% formic acid (solvent B), increasing linearly to 40 % B over 55 minutes, increasing to 100% B over 3 min and held for 3 min before returning to initial conditions.

References cited

(1) Flynn, J. M.; Hubley, R.; Goubert, C.; Rosen, J.; Clark, A. G.; Feschotte, C.; Smit, A. F. RepeatModeler2 for Automated Genomic Discovery of Transposable Element Families. *Proceedings of the National Academy of Sciences* **2020**, *117* (17), 9451–9457. https://doi.org/10.1073/PNAS.1921046117.

(2) Vanheule, A.; Audenaert, K.; Warris, S.; van de Geest, H.; Schijlen, E.; Höfte, M.; De Saeger, S.; Haesaert, G.; Waalwijk, C.; van der Lee, T. Living Apart Together: Crosstalk between the Core and Supernumerary Genomes in a Fungal Plant Pathogen. *BMC Genomics* **2016**, *17* (1), 670. https://doi.org/10.1186/s12864-016-2941-6.

(3) Van Wyk, S.; Harrison, C. H.; Wingfield, B. D.; De Vos, L.; Van Der Merwe, N. A.; Steenkamp, E. T. The RIPper, a Web-Based Tool for Genome-Wide Quantification of Repeat-Induced Point (RIP) Mutations. *PeerJ* **2019**, *2019* (7), e7447. https://doi.org/10.7717/peerj.7447.

(4) Palmer, J. M.; Stajich, J. Funannotate v1.8.1: Eukaryotic Genome Annotation. **2020**. https://doi.org/10.5281/ZENODO.4054262.

(5) Blin, K.; Shaw, S.; Kloosterman, A. M.; Charlop-Powers, Z.; van Wezel, G. P.; Medema, M. H.; Weber, T. antiSMASH 6.0: Improving Cluster Detection and Comparison Capabilities. *Nucleic Acids Research* **2021**, *49* (W1), W29–W35. https://doi.org/10.1093/NAR/GKAB335.

(6) Teufel, F.; Almagro Armenteros, J. J.; Johansen, A. R.; Gíslason, M. H.; Pihl, S. I.; Tsirigos, K. D.; Winther, O.; Brunak, S.; von Heijne, G.; Nielsen, H. SignalP 6.0 Predicts All Five Types of Signal Peptides Using Protein Language Models. *Nat Biotechnol* **2022**, *40* (7), 1023–1025. https://doi.org/10.1038/s41587-021-01156-3.

(7) Jones, P.; Binns, D.; Chang, H.-Y.; Fraser, M.; Li, W.; McAnulla, C.; McWilliam, H.; Maslen, J.; Mitchell, A.; Nuka, G.; Pesseat, S.; Quinn, A. F.; Sangrador-Vegas, A.; Scheremetjew, M.; Yong, S.-Y.; Lopez, R.; Hunter, S. InterProScan 5: Genome-Scale Protein Function Classification. *Bioinformatics* **2014**, *30* (9), 1236–1240. https://doi.org/10.1093/bioinformatics/btu031.

(8) Cantalapiedra, C. P.; Hernández-Plaza, A.; Letunic, I.; Bork, P.; Huerta-Cepas, J. EggNOG-Mapper v2: Functional Annotation, Orthology Assignments, and Domain Prediction at the Metagenomic Scale. *Molecular Biology and Evolution* **2021**, *38* (12), 5825–5829. https://doi.org/10.1093/molbev/msab293.

(9) Käll, L.; Krogh, A.; Sonnhammer, E. L. L. A Combined Transmembrane Topology and Signal Peptide Prediction Method. *Journal of Molecular Biology* **2004**, *338* (5), 1027–1036. https://doi.org/10.1016/j.jmb.2004.03.016.
(10) Brown, D. W.; Kim, H.-S.; McGovern, A. E.; Probyn, C. E.; Proctor, R. H. Genus-Wide Analysis of Fusarium Polyketide Synthases Reveals Broad Chemical Potential. *Fungal Genetics and Biology* **2022**, *160*, 103696. https://doi.org/10.1016/j.fgb.2022.103696.
(11) Hansen, F. T.; Gardiner, D. M.; Lysøe, E.; Fuertes, P. R.; Tudzynski, B.; Wiemann, P.; Sondergaard, T. E.; Giese, H.; Brodersen, D. E.; Sørensen, J. L. An Update to Polyketide Synthase and Non-Ribosomal Synthetase Genes and Nomenclature in *Fusarium*. *Fungal Genetics and Biology* **2015**, *75*, 20–29. https://doi.org/10.1016/j.fgb.2014.12.004.
(12) Nickles, G. R.; Oestereicher, B.; Keller, N. P.; Drott, M. T. Mining for a New Class of Fungal Natural Products: The Evolution, Diversity, and Distribution of Isocyanide Synthase Biosynthetic Gene Clusters. *Nucleic Acids Research* **2023**, *51* (14), 7220–7235. https://doi.org/10.1093/nar/gkad573.
(13) Lysøe, E.; Frandsen, R. J. N.; Divon, H. H.; Terzi, V.; Orrù, L.; Lamontanara, A.; Kolseth, A. K.; Nielsen, K. F.; Thrane, U. Draft Genome Sequence and Chemical Profiling of Fusarium Langsethiae, an Emerging Producer of Type A Trichothecenes. *International Journal of Food Microbiology* **2016**, *221*, 29–36. https://doi.org/10.1016/j.ijfoodmicro.2016.01.008.
(14) Niehaus, E. M.; Kim, H. K.; Münsterkötter, M.; Janevska, S.; Arndt, B.; Kalinina, S. A.; Houterman, P. M.; Ahn, I. P.; Alberti, I.; Tonti, S.; Kim, D. W.; Sieber, C. M. K.; Humpf, H. U.; Yun, S. H.; Güldener, U.; Tudzynski, B. Comparative Genomics of Geographically Distant Fusarium Fujikuroi Isolates Revealed Two Distinct Pathotypes Correlating with Secondary Metabolite Profiles. *PLoS Pathogens* **2017**, *13* (10), 1–38. https://doi.org/10.1371/journal.ppat.1006670.
(15) King, R.; Urban, M.; Hammond-Kosack, M. C. U.; Hassani-Pak, K.; Hammond-Kosack, K. E. The Completed Genome Sequence of the Pathogenic Ascomycete Fungus *Fusarium Graminearum*. *BMC Genomics* **2015**, *16* (1), 1–21. https://doi.org/10.1186/s12864-015-1756-1.
(16) Bandi, V.; Gutwin, C. Interactive Exploration of Genomic Conservation. *Graphics interface (Conference).* 2020.
(17) Katoh, K.; Standley, D. M. MAFFT Multiple Sequence Alignment Software Version 7: Improvements in Performance and Usability. *Molecular Biology and Evolution* **2013**, *30* (4), 772–780. https://doi.org/10.1093/molbev/mst010.
(18) Capella-Gutiérrez, S.; Silla-Martínez, J. M.; Gabaldón, T. trimAl: A Tool for Automated Alignment Trimming in Large-Scale Phylogenetic Analyses. *Bioinformatics* **2009**, *25* (15), 1972–1973. https://doi.org/10.1093/bioinformatics/btp348.
(19) Minh, B. Q.; Schmidt, H. A.; Chernomor, O.; Schrempf, D.; Woodhams, M. D.; Von Haeseler, A.; Lanfear, R.; Teeling, E. IQ-Tree 2: New Models and Efficient Methods for Phylogenetic Inference in the Genomic Era. *Molecular Biology and Evolution* **2020**, *37* (5), 1530–1534. https://doi.org/10.1093/molbev/msaa015.
(20) Kalyaanamoorthy, S.; Minh, B. Q.; Wong, T. K. F.; Von Haeseler, A.; Jermiin, L. S. ModelFinder: Fast Model Selection for Accurate Phylogenetic Estimates. *Nature Methods* **2017**, *14* (6), 587–589. https://doi.org/10.1038/nmeth.4285.
(21) Hoang, D. T.; Chernomor, O.; von Haeseler, A.; Minh, B. Q.; Vinh, L. S. UFboot2: Improving the Ultrafast Bootstrap Approximation. *Molecular Biology and Evolution* **2018**, *35* (2), 518–522. https://doi.org/10.5281/zenodo.854445.

Growth Media

CMC Medium (1L)

Carboxymethylcellulose	15 g
NH_4NO_3	1 g
KH_2PO_4	1 g
$MgSO_4 \cdot 7H_2O$	0.5 g
Yeast Extract	1 g
MilliQ Water	to 1.0 L

CYA Medium (1L)

$NaNO_3$	3 g
KH_2PO_4	1 g
KCl	0.5 g
$MgSO_4 \cdot 7H_2O$	0.5 g
$FeSO_4 \cdot 7H_2O$	10 mg
Yeast Extract	5 g
Sucrose	30 g
MilliQ Water	to 1.0 L
Trace Element Solution 1	1000 µL

Trace Element Solution 1 (100mL)

$ZnSO_4 \cdot 7H_2O$	1 g
$CuSO_4 \cdot 5H_2O$	0.5 g
MilliQ Water	100 mL

MM Minimal Medium with ^{15}N enrichment (1L)

$NaNO_3$ (99.9% ^{15}N)	1.875 g
$NaNO_3$	1.125 g
KH_2PO_4	1 g
KCl	0.5 g
$MgSO_4 \cdot 7H_2O$	0.5 g
$FeSO_4 \cdot 7H_2O$	10 mg
Sucrose	30 g
MilliQ Water	to 1.0 L
Trace Elements Solution 2	200 µL

Trace Element Solution 2 (100mL, pH 6.5)

$ZnSO_4 \cdot 7H_2O$	2.2 g
H_3BO_3	1.1 g
$MnCl_2 \cdot 4H_2O$	0.5 g
$FeSO_4 \cdot 7H_2O$	0.5 g
$CoCl_2 \cdot 6H_2O$	0.17 g
$CuSO_4 \cdot 5H_2O$	0.16 g
$Na_2MoO_4 \cdot 2H_2O$	0.15 g
Na_4EDTA	5.0 g
Heat to 60°C to dissolve, then cool.	
KOH to adjust pH	
MilliQ Water	100 mL

MMK2 Medium (1L)

Mannitol	40 g
Yeast Extract	5 g
Murashige & Skoog Salts	4.3 g
MilliQ Water	to 1.0 L

PDB Medium (1L)

Difco Potato Dextrose Broth	36.0 g
MilliQ Water	to 1.0 L

YES Medium (1L)

Yeast Extract	20 g
Sucrose	150 g
$MgSO_4 \cdot 7H_2O$	0.5 g
MilliQ Water	to 1.0 L

YES+IO Medium (1L)

YES Medium	1.0 L
Instant Ocean	18 g

Protoplasting/CHEF digestion Buffers

EDTA Buffer (0.5M, 1L, pH 8.0)

Disodium EDTA dihydrate	186.1 g
NaOH to adjust pH	
MilliQ Water	to 1.0 L

ET Buffer (1L, pH 8.0)

EDTA	186.1 g
Tris-HCL, 1mM	10 mL
MilliQ Water	to 1.0 L

Lysis Buffer (50mL)

EDTA (1M, pH 9.3)	25m L
2% N-lauroyl sarcosine, sodium salt	25m L
Proteinase K (added immediately prior to use)	25 uL/mL of buffer needed

N-lauroyl sarcosine solution (0.1L)

N-lauroyl sarcosine, sodium salt	2.0 g
MilliQ water	to 100 mL

Protoplast Buffer (0.5L, pH 5.6)

$MgSO_4 \cdot 7H_2O$	148 g
MilliQ Water	to 0.5 L

SE Buffer (0.5L, pH 8.0)

Sorbitol	91.36 g
EDTA, 0.5M	50 mL
MilliQ Water	to 0.5 L

STC Buffer (0.5L)

Sorbitol	109.3 g
Tris-HCl, 1M, pH 7.5	5 mL
$CaCl_2 \cdot 2H_2O$	3.67 g

TE Buffer (0.1L, pH 8.0)

1M Tris-HCl	1 mL
0.5M EDTA (pH 8.0)	0.2 mL
MilliQ Water	to 100 mL

Supporting Information 2: Newick Tree

Newick tree generated from IQTree2 analysis of fungal NRPS condensation domains.

(fusadapamide_C1_nMT:0.2871622298,fusadapamide_C2_nMT:0.1367236744,((((((((((((((((((((fusadapa
mide_C3:0.6374014685,destruxin_C1:0.4695122329)29.3/99:0.0512514815,sansalvamide_C2:0.564404
1112)33.2/98:0.0670214086,sansalvamide_C3:0.6886750831)20.5/75:0.0335728753,destruxin_C3:0.485
3514561)0/33:0.0382643010,sansalvamide_C1:0.7606853796)91.5/97:0.1383110075,tentoxin_C2:0.681
8618265)100/100:0.3407326552,((beauvericin_C1:2.2468024032,(cyclochlorotine_C2:1.0213877200,cyc
lochlorotine_C3:1.1237203314)80.1/91:0.1313904083)28.2/80:0.0422186849,(cyclochlorotine_C1:0.8635
194608,cyclochlorotine_C4:1.2125653372)95.4/93:0.2591322538)96.5/96:0.1493498685)57.2/88:0.0727
027419,((((((((aculeacin_C6:0.5819627363,aculeacin_C5:0.5159732192)5.8/90:0.0657058550,aculeacin
_C1:0.6074743146)94.3/99:0.1009429580,(aculeacin_C3:0.5921958707,aculeacin_C4:0.5712404811)28
.5/95:0.0357370690)82.1/97:0.1126680853,aculeacin_C2:0.6675462495)100/100:0.2847336039,((b2raf_
C2:0.2377723803,(((((b2raf_C3:0.2028551009,b2raf_C6:0.3307413928)35.2/79:0.0553628823,b2raf_C4:
0.2372944860)84.4/78:0.0298649330,b2raf_C5:0.2355427340)83.3/82:0.0606779245,W493_C4:0.41324
25441)68.3/83:0.0384261671,W493_C5:0.4080138345)81.5/84:0.0922591751)100/100:0.6346419098,((
beauveriolide_C3:0.6500874923,emericellamide_C3:0.7448192693)20.7/92:0.1290242284,emericellamid
e_C5:0.9140460683)92.5/99:0.1426574386)92.9/99:0.1446786776)98.5/100:0.2186399078,((aspercrypti
n_C1:0.8129793421,aspercryptin_C5:0.9791733942)11.8/93:0.1022198905,aspercryptin_C3:0.88927041
34)11.6/47:0.0724865179)91/98:0.1134251944,((cyclo_C2:0.7658128100,fusahexin_C4:0.8735706081)9
6.4/100:0.2450160041,fusahexin_C2:1.0502915357)99.8/100:0.2833304093)51.8/58:0.0586612975,((api
cidin_C3:0.7321613753,HCToxin_C3:0.7775884555)100/100:0.8751595064,(apicidin_C2:0.6132560223,
HCToxin_C2:0.6646326463)100/100:0.5800977962)97.5/98:0.2332090421)42.4/50:0.0450858993,((leuci
nostatin_C2:1.1456683092,((leucinostatin_C4:0.7675981741,(leucinostatin_C7:0.8416823698,leucinostat
in_C8:0.8562061543)15.6/96:0.0919459105)93.5/100:0.1430909451,(leucinostatin_C5:0.9546941110,(le
ucinostatin_C9:0.7021191005,leucinostatin_C10:0.8315402643)74.4/100:0.1298669131)94.5/99:0.16461
58870)81/98:0.0619400034)90.2/100:0.1246348799,(leucinostatin_C3:0.8505273543,leucinostatin_C6:1.
1428240812)69.1/89:0.1821116771)88.1/87:0.1264065162)65/56:0.0543507573)73.9/67:0.0997970422,(
((chrysogine_C1:0.9699225386,(serinocyclin_C3:0.8311949776,serinocyclin_C4:0.7096728476)94.3/100:
0.1942900600)36.9/85:0.1054469006,(((ergotamine_LPSB_C1:1.0830808247,psychrophylin_C1:0.79839
57816)91.5/97:0.1813185827,(notoamide_C1:1.0515273219,okaramine_C1:1.3005862828)4.7/34:0.042
1021590)81.3/76:0.0761748536,((fumiquinazoline_C1:0.4554702629,tryptoquialanine_C1:0.5602539982
)99.5/100:0.2833376026,(oxepinamide_C1:0.7441821852,acetylaszonalenin_C1:0.6858677394)99.6/100
:0.3460640566)99.1/100:0.2761400250)87.9/75:0.1070708906)94.8/84:0.1284609966,(ergotamine_LPS
A1_C1:0.6679600092,ergotamine_LPSC_C1:1.2374419587)100/100:0.8232050045)47.9/92:0.04633867
15)98.4/99:0.3048165322,(apicidin_C4_Ct:0.8097219012,HCToxin_C4_Ct:0.8273137223)100/100:1.153
5744700)72.4/97:0.0778271311,penicillin_C1:2.0992954725)86.7/88:0.1487010591,(aspergillicin_C1:2.4
483911596,(aspergillicin_C2:1.0039031422,(aspergillicin_C4:0.8184277955,aspergillicin_C6:0.75222554
55)88.8/100:0.2323709565)99.2/100:0.8386773366)88.9/95:0.2842464421)59.9/82:0.0474462148,(((((Ab
T1_C9_Ct:0.5166087425,cyclosporine_C12_Ct:0.5738575889)37.7/98:0.0989934134,KK1_C10_Ct:0.50
36781666)95.8/99:0.1843161822,beauvericin_C3_Ct:0.7172137098)100/100:0.7086849533,((((((aculeac
in_C7_Ct:0.9980368357,(b2raf_C9_Ct:0.2385250295,W493_C7_Ct:0.5102658994)100/100:1.03704144
50)88.2/100:0.2680692905,emericellamide_C6_Ct:0.9024691719)97.8/100:0.3286206238,(cyclo_C4_Ct:
0.6697436868,fusahexin_C6_Ct:0.8355037014)95.1/100:0.2052567450)99.6/100:0.3636025861,(cycloch
lorotine_C5_Ct:1.9075442371,((destruxin_C6_Ct:0.6272024449,sansalvamide_C5_Ct:0.7176710983)97.
2/100:0.3057488388,tentoxin_C4_Ct:0.9258155785)64.1/100:0.2081686964)94/100:0.2733068815)78.7/
81:0.1199978060,((ergotamine_LPSA1_C3_Ct:1.6237212315,serinocyclin_C8_Ct:0.8796926870)39.7/92
:0.2124159428,((((fumiquinazoline_C3_Ct:0.3465393729,tryptoquialanine_C3_Ct:0.4315257735)100/100
:0.3938345075,oxepinamide_C3_Ct:0.9312197458)54.3/90:0.1255472962,psychrophylin_C2_Ct:0.87754
82174)77.4/95:0.2241979332,notoamide_C2_Ct:1.1494815497)97.8/100:0.2701463841)93.1/98:0.14792
39712)88.2/80:0.1501903847,chrysogine_C2_Ct:1.4325003142)65.6/97:0.1125277213)99.6/100:0.64748
90760,aspergillicin_C3_DCL:2.1671334156)58.9/91:0.1852531252)95.1/92:0.2315946730,((((((((apicidin

_C1_DCL:0.5680005869,HCToxin_C1_DCL:0.7933224235)100/100:0.5397123252,fusaoctaxin_C2_pseu
doDCL:1.1939093378)70.2/94:0.0965372186,leucinostatin_C1:1.2364660253)93.5/91:0.1877899787,(fus
ahexin_C3_DCL:1.0008402962,fusaoctaxin_C1_:1.3398863408)39.8/45:0.1081207182)0/12:0.00000287
82,((((ergotamine_LPSA1_C2:1.3546326726,serinocyclin_C6_pseudoDCL:0.8968587231)68.8/22:0.1031
916751,(serinocyclin_C7_DCL:0.7028005312,serinocyclin_C5_DCL:1.0097124449)88.2/97:0.219874379
8)32.2/24:0.0930473489,serinocyclin_C2_pseudoDCL:1.1826850670)65.4/22:0.0983435024,okaramine_
C2_Ct:1.8949811056)35.7/20:0.0382945996)87.8/53:0.0723531070,(((((aspercryptin_C2_DCL:0.825272
9005,(cyclo_C3_DCL:0.7594861719,fusahexin_C5_DCL:0.8271115515)98.1/100:0.2575407909)34.6/76:
0.0534768039,aspercryptin_C4:1.0965888256)34.2/74:0.0586881941,(cyclo_C1_DCL:0.7480526244,fus
ahexin_C1_DCL:0.6592443255)99.9/100:0.3391316127)68.8/89:0.1161050963,((fusaoctaxin_C3_DCL:0.
4078220627,fusaoctaxin_C5_DCL:0.4065693003)100/100:0.6179019713,(fusaoctaxin_C4_DCL:0.45919
62909,fusaoctaxin_C6_DCL:0.3420060521)100/100:0.5834238144)95.4/100:0.1917213637)39.6/51:0.05
91739553,((((((((b2raf_C7_DCL:0.3192038387,b2raf_C8_DCL:0.2621839164)36.9/68:0.0840821462,W4
93_C2_DCL:0.3186714082)58.3/59:0.0566291538,W493_C3:0.3375597684)48.6/57:0.0389506226,W49
3_C6_DCL:0.3635673282)100/100:0.6192299222,(beauveriolide_C4_DCL_Ct:0.8383144829,emericella
mide_C4:0.5075616742)98.2/100:0.2273071840)88.9/100:0.1174512273,n_acetyl_tryptophan_C1_DCL:
2.1859251647)81.9/83:0.0603517707,(beauveriolide_C2_pseudoDCL:0.8954568834,emericellamide_C2
_pseudoDCL:0.5509492276)99.8/100:0.3155819096)95.9/84:0.1600643959,fusaoctaxin_C7_DCL:0.913
6234876)89.7/78:0.1010455895)77.3/53:0.0337094845)94.3/65:0.0917451775,((destruxin_C2_DCL:0.82
78538568,fusadapamide_C4:0.9808136967)100/100:0.5408477116,((fumiquinazoline_C2_DCL:0.523589
4035,tryptoquialanine_C2_DCL:0.4948631737)100/100:0.7175240038,oxepinamide_C2_DCL:1.0584479
265)81.7/99:0.1863498600)71.5/69:0.1266619993)94.4/95:0.1493789699,((b2raf_C1_pseudoDCL:0.240
2431642,W493_C1_DCL:0.2810021239)100/100:1.3868592950,(beauveriolide_C1:0.8702843507,emeri
cellamide_C1_pseudoDCL:0.5186437870)99.6/100:0.4462471077)96.3/100:0.2846522014)98.7/100:0.3
769826124)100/100:0.6840123569,(((((((((((acetylaszonalenin_E1_Ct:0.8668342340,oxepinamide_E1:0.
8119244145)98.6/100:0.2818668577,tryptoquialanine_E1:0.7303400974)59.3/99:0.1181013745,((serinoc
yclin_E2:0.6945530596,serinocyclin_E4:0.7506407432)51.7/100:0.0738261729,serinocyclin_pseudoE3:0
.9700675999)19.2/94:0.0833015330)98.3/100:0.1391594656,((Cyclo_E1:0.8056452750,Cyclo_E2:0.772
8922727)93.6/95:0.1665566911,aspercryptin_E1:0.7699738659)67.8/87:0.0516275654)56.8/90:0.065231
0899,(apicidin_E1:0.4732562196,HCToxin_E1:0.5512840285)100/100:0.5824208189)84.1/92:0.0546369
331,((emericellamide_pseudoE2:1.1560444916,(((B2raf_E2:0.1785266505,B2raf_E3:0.2696297289)99.5
/100:0.1380679650,(W493_E2:0.3192953172,W493_E4:0.4180170131)61.5/83:0.0693395685)86.6/84:0.
0697030570,W493_E3:0.3143981896)100/100:0.4559793875)98.2/99:0.2504429431,((fusaoctaxin_E2:0.
4353157995,fusaoctaxin_E4:0.3508067093)100/100:0.5252612636,(fusaoctaxin_E3:0.3332394472,fusa
octaxin_E5:0.3673267229)100/100:0.4644286569)95/93:0.1981488500)78.5/90:0.0666987500)95.7/91:0
.1486298265,(fusaoctaxin_pseudoE1:1.7001494096,fusaoctaxin_E6:0.7889557961)87.8/87:0.19793631
82)73.4/73:0.0972917533,N-
acetyltryptophan_E1:1.3677334610)66.5/68:0.0993123704,serinocyclin_pseudoE1:1.4237046083)90.4/8
5:0.2451279112,(emericellamide_pseudoE1:2.7111496851,aspergillicin_E1:1.8344459815)0/50:0.005120
1811)72.3/76:0.3219786575,penicillin_E1:1.8921421889)100/100:0.9605205704)97.5/100:0.5534585254
,penicillin_C2:1.6358594192)100/100:0.9243473031,aspergillicin_C5_nMT:0.5099856341)31.6/88:0.0309
186459,(((((((((AbT1_C1_nMT:0.0904756961,(AbT1_C5:0.1281933844,AbT1_C6_nMT:0.0836849337)85
.8/90:0.0352051535)63.1/89:0.0172631870,(AbT1_C3_nMT:0.0000028006,AbT1_C8_nMT:0.000000999
9)100/100:0.0806331096)85.6/91:0.0319919476,AbT1_C4:0.2953663517)99.6/100:0.1056207042,(AbT1
_C2:0.0000021429,AbT1_C7:0.0000024807)100/100:0.3665065773)98.7/70:0.1088145137,(((KK1_C1:0.
3208322751,(KK1_C3:0.1591938041,KK1_C9:0.2541053422)99/100:0.0649223629)85/43:0.022439098
3,(((KK1_C4_nMT:0.2338066155,(KK1_C6_nMT:0.2760343633,KK1_C7:0.2273126461)43/57:0.0433098
448)73.4/66:0.0355318576,KK1_C5_nMT:0.2662730942)94.6/88:0.0577846283,KK1_C8_nMT:0.240329
1505)88.2/83:0.0287869142)7/28:0.0109773120,KK1_C2_nMT:0.2481678652)98.5/67:0.0561750448)76.
1/52:0.0142803544,(cyclosporine_C4_nMT:0.3177503650,cyclosporine_C5_nMT:0.3055667545)94.1/97:
0.0589467412)86.7/59:0.0220438284,((cyclosporine_C1:0.5605128462,cyclosporine_C8_nMT:0.350988
7994)3.4/63:0.0247469892,cyclosporine_C6:0.3886283508)92.8/95:0.0448980015)89.2/62:0.025886376

8,((cyclosporine_C2_nMT:0.2895355635,cyclosporine_C3_nMT:0.3546804537)87.6/99:0.0339286349,(cy
closporine_C7_nMT:0.3216430833,cyclosporine_C11:0.2750567123)77.3/78:0.0553612888)94/77:0.042
8209341)43/60:0.0157206205,(beauvericin_C2_nMT:0.4418313823,(cyclosporine_C9:0.3748892550,cycl
osporine_C10_nMT:0.3097833545)79.2/81:0.0595707197)37.7/62:0.0309691939)100/100:0.2568273633
)96/98:0.0980495712,tentoxin_C3_nMT:0.4858830861)96.7/98:0.0902730347,destruxin_C4_nMT:0.3720
899625)65.9/96:0.0284103075,((destruxin_C5_nMT:0.3353170262,tentoxin_C1_nMT:0.4279476203)87/1
00:0.0619070884,sansalvamide_C4:0.5528254371)36.5/97:0.0472791738)100/100:0.3056489412);

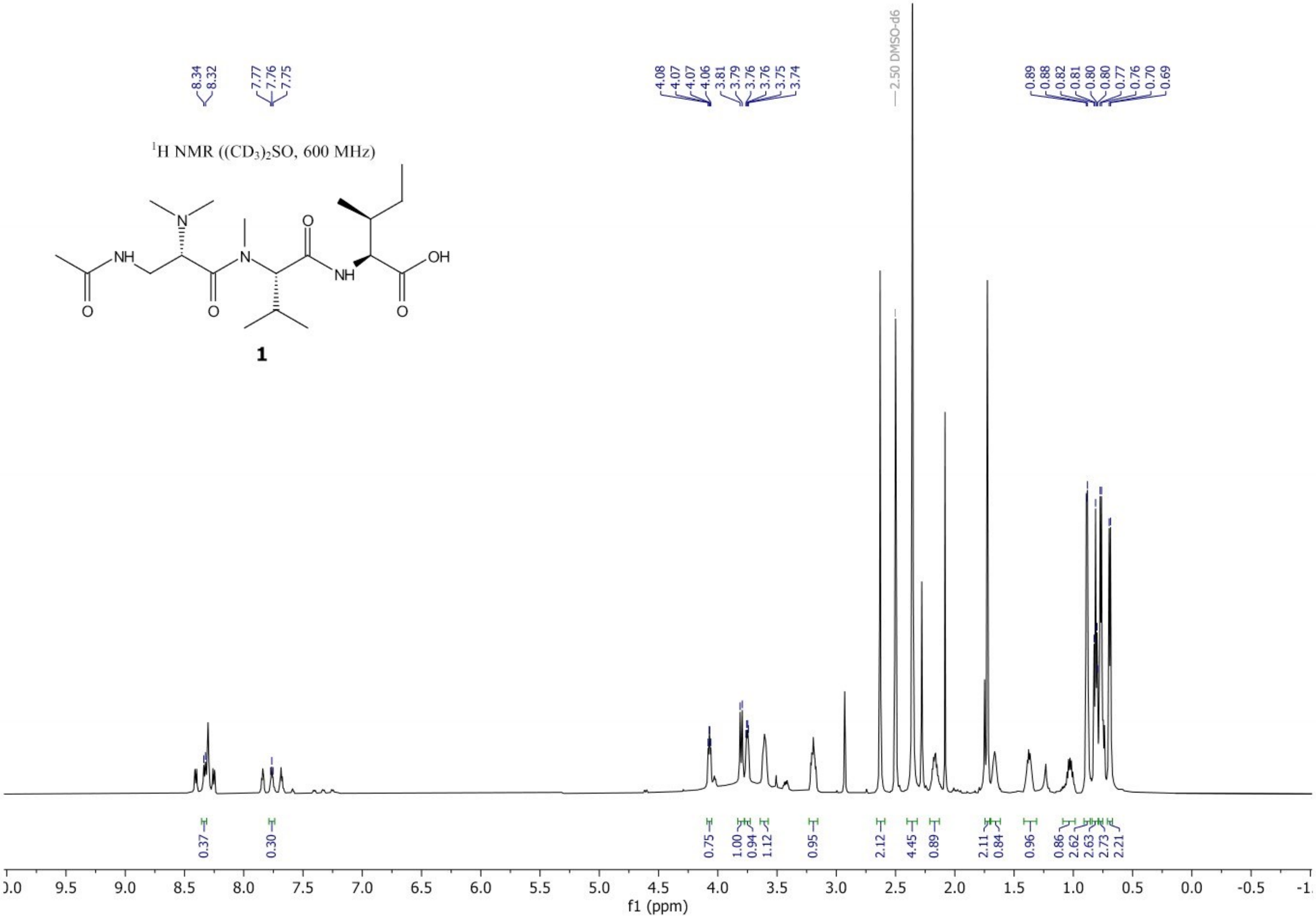

Figure S1. ^{1}H NMR ($(CD_3)_2SO$, 600 MHz) of compound **1**

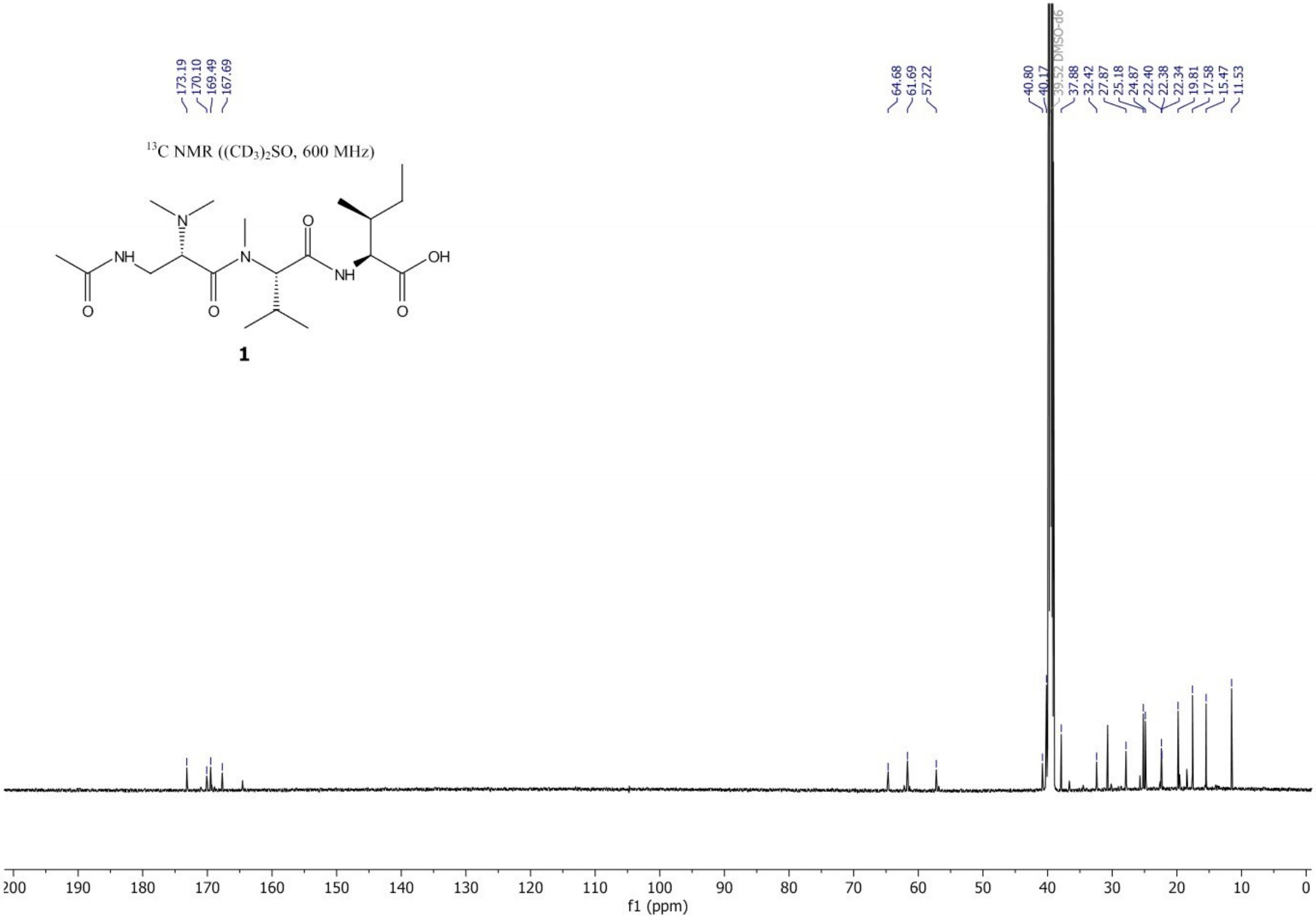

Figure S2. ^{13}C NMR ($(CD_3)_2SO$, 600 MHz) of compound **1**

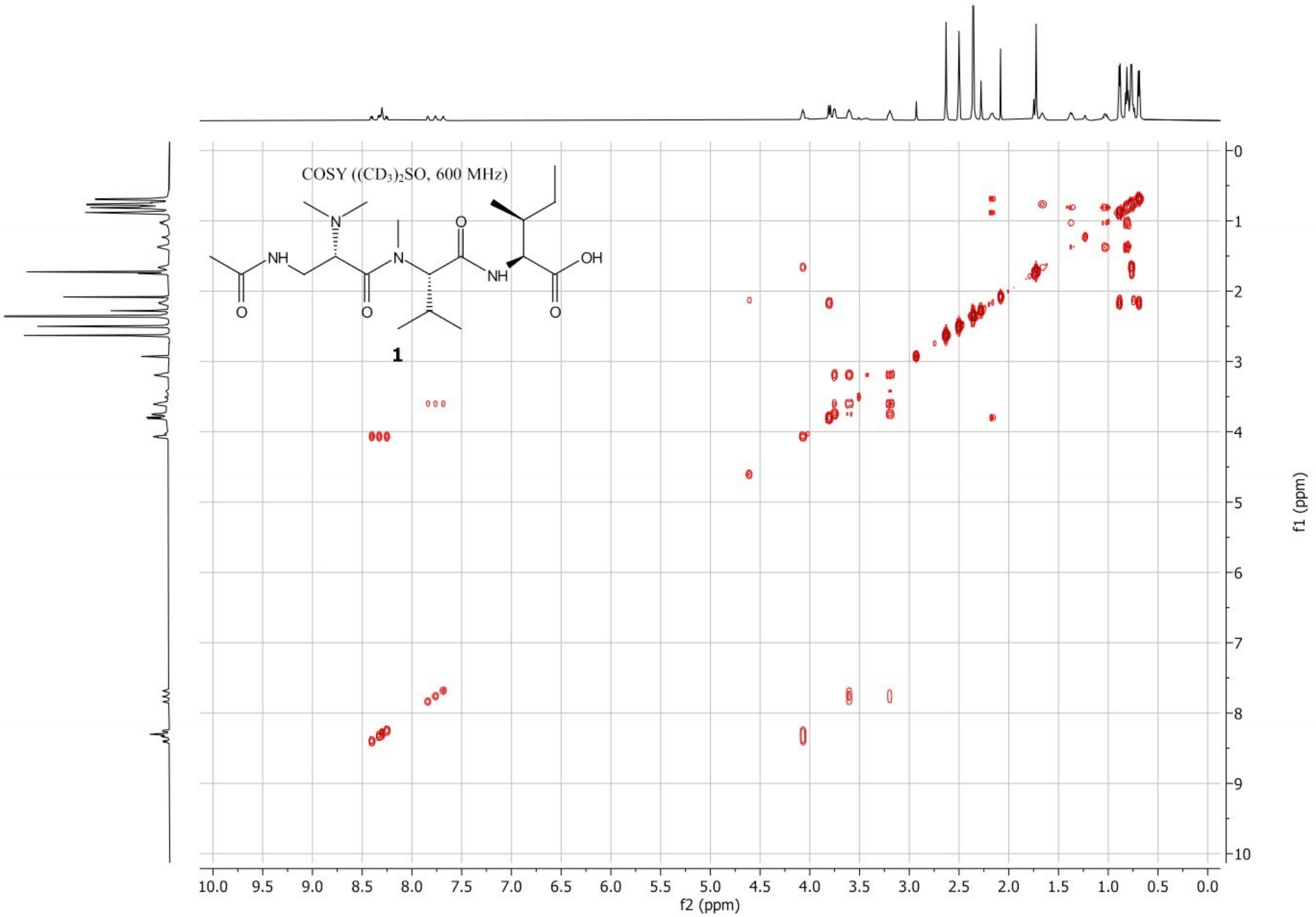

Figure S3. COSY ($(CD_3)_2SO$, 600 MHz) of compound **1**

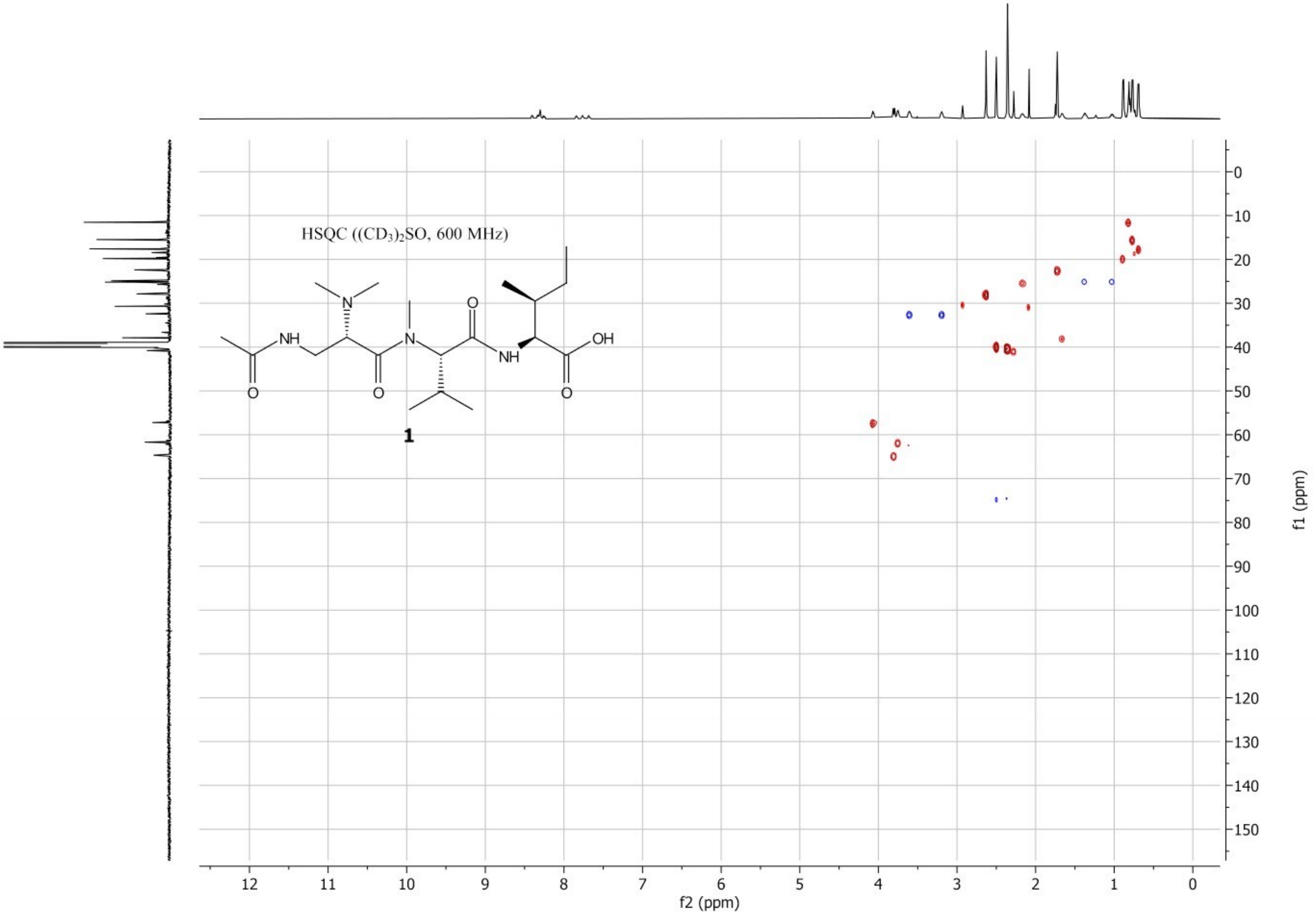

Figure S4. HSQC ($(CD_3)_2SO$, 600 MHz) of compound **1**

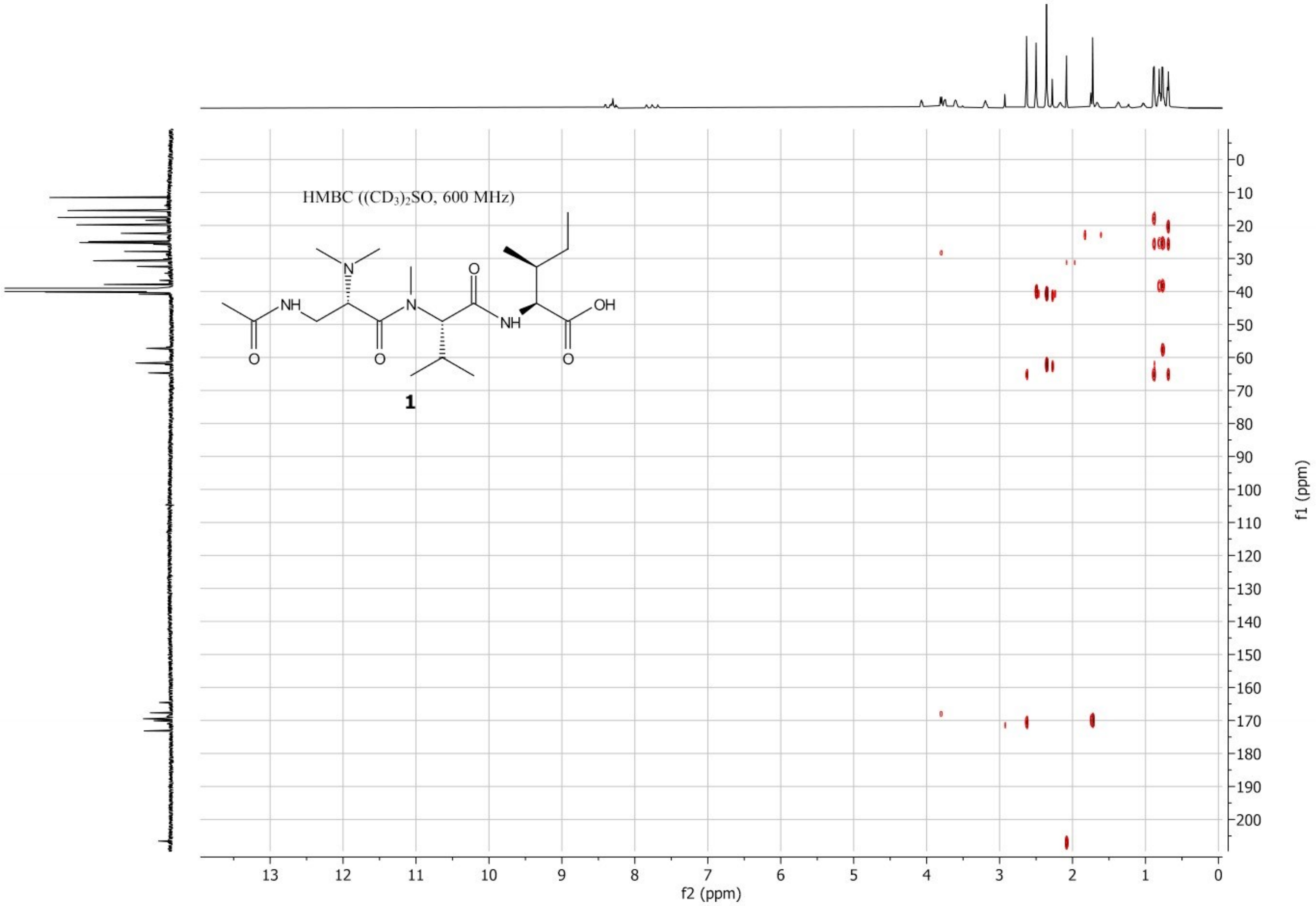

Figure S5. HMBC ($(CD_3)_2SO$, 600 MHz) of compound **1**

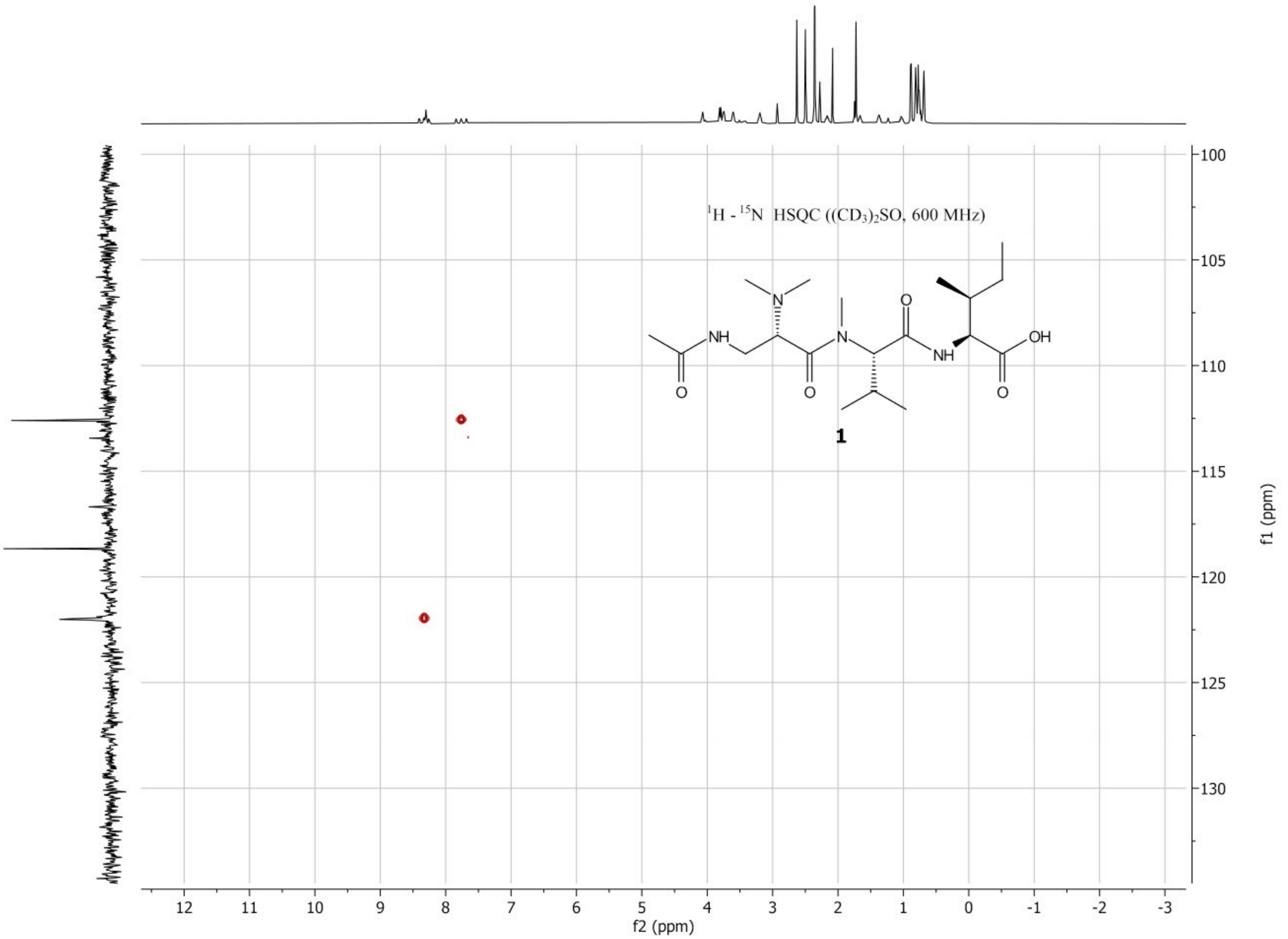

Figure S6. ^1H - ^{15}N HSQC $((CD_3)_2SO$, 600 MHz) of compound **1**

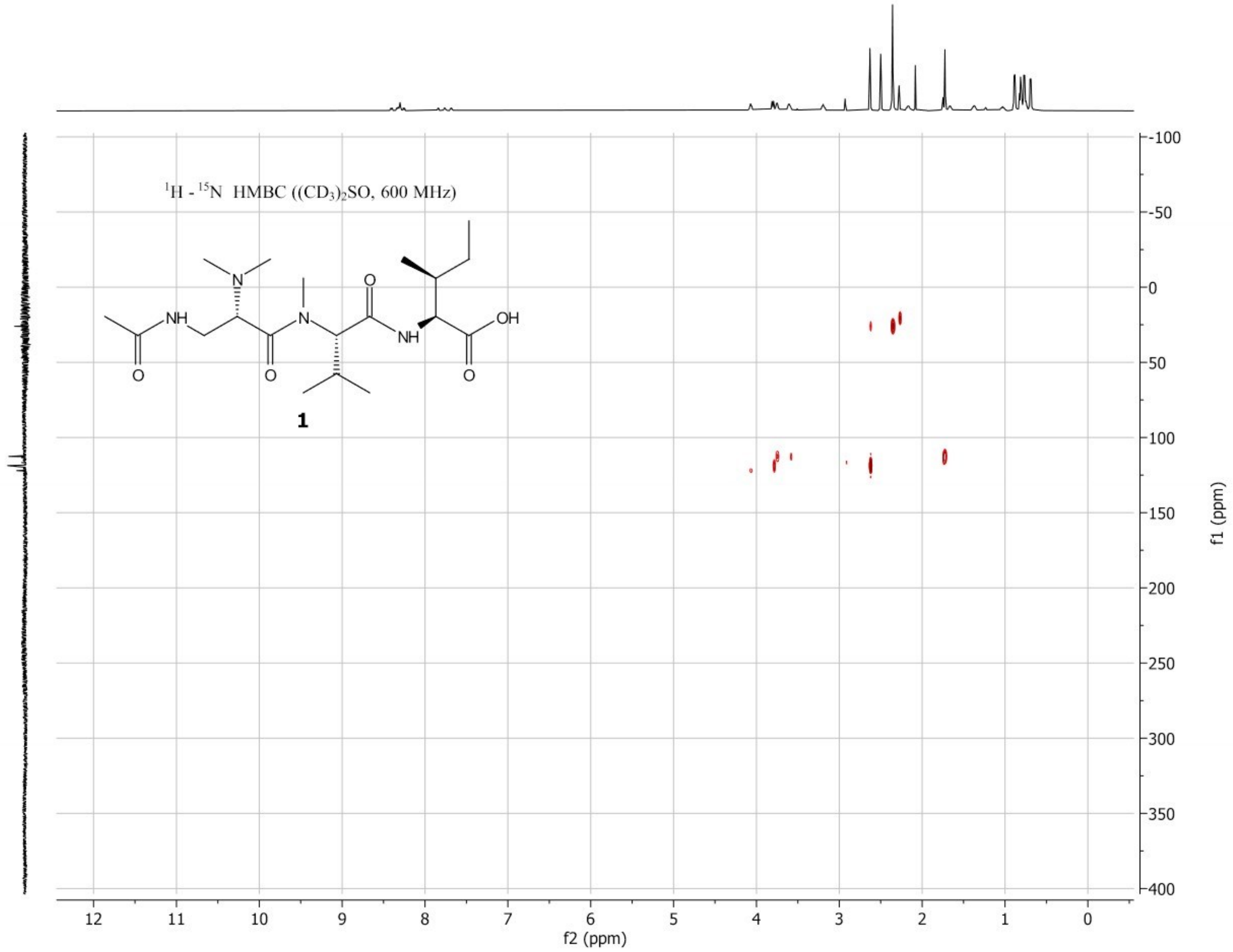

Figure S7. ^{1}H - ^{15}N HMBC ($(CD_3)_2SO$, 600 MHz) of compound **1**

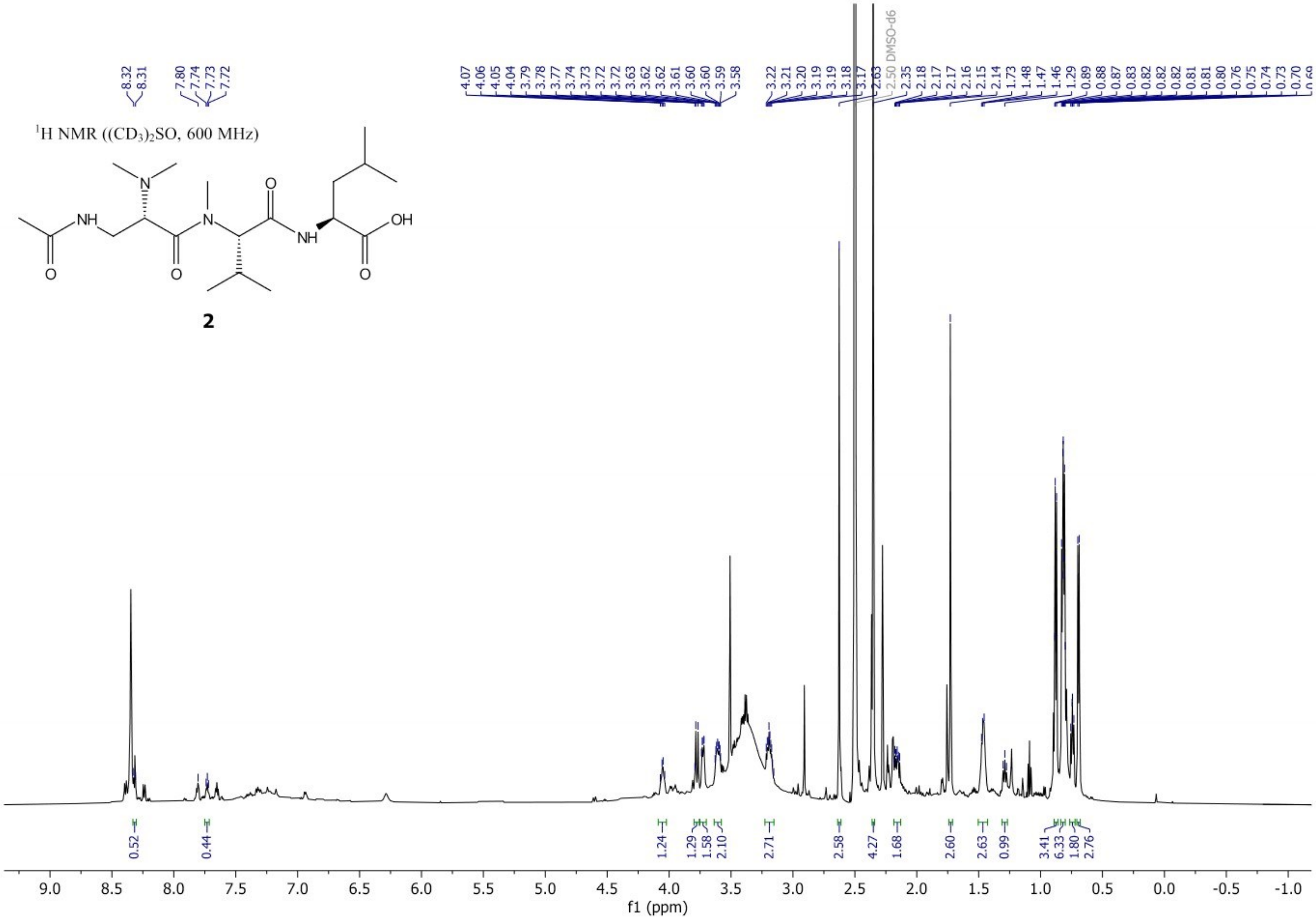

Figure S8. ^{1}H NMR ($(CD_3)_2SO$, 600 MHz) of compound **2**

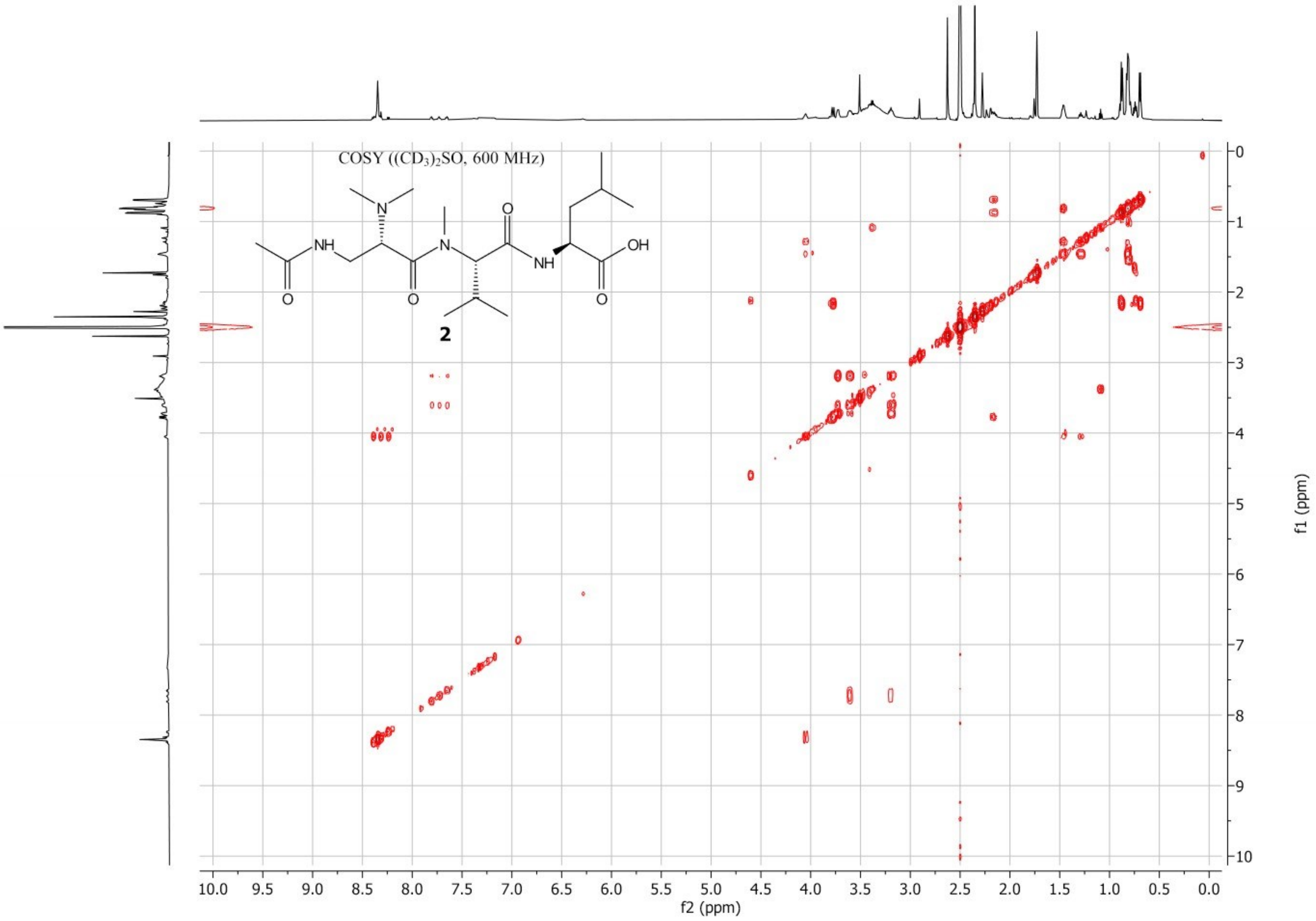

Figure S9. COSY ((CD$_3$)$_2$SO, 600 MHz) of compound **2**

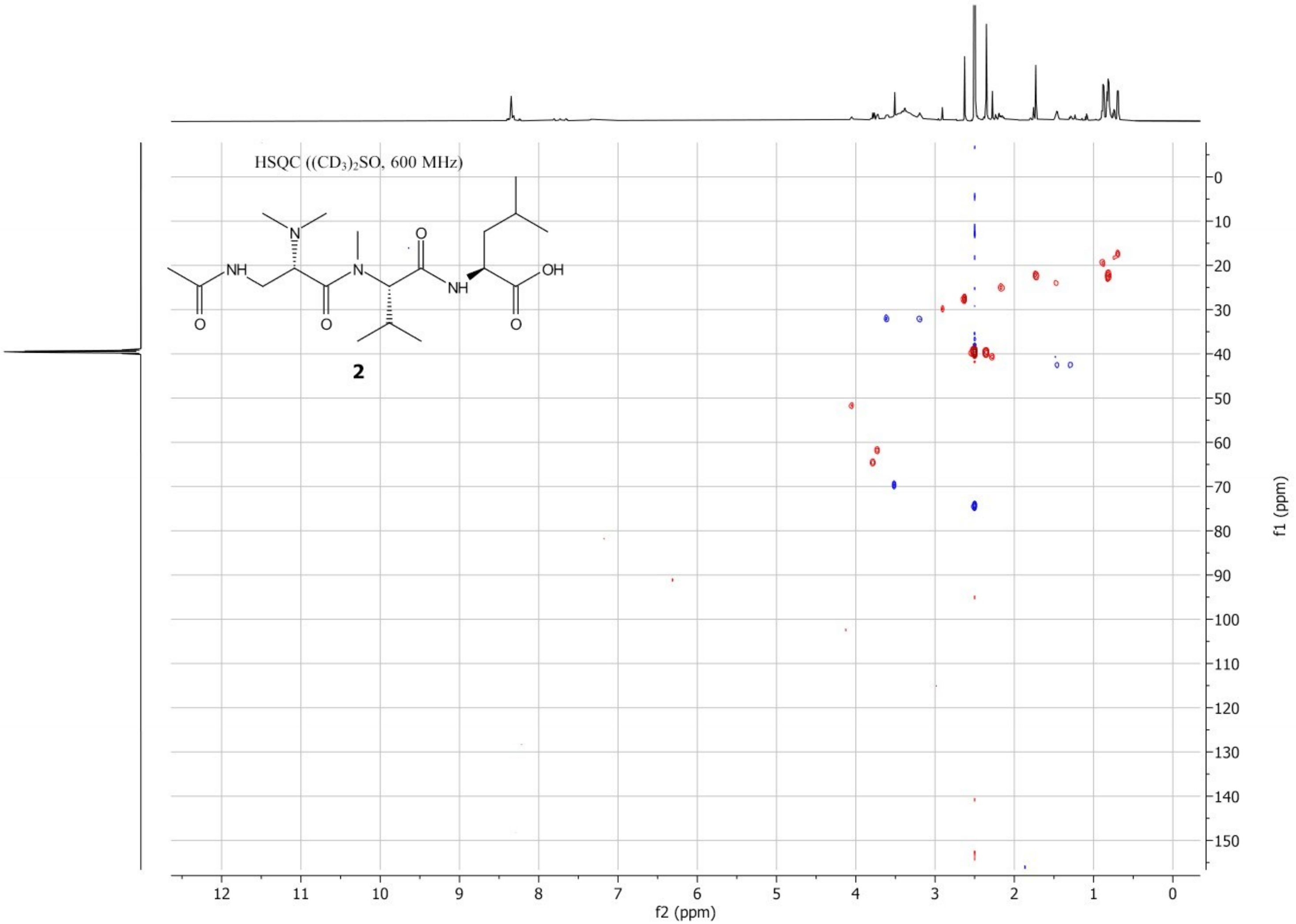

Figure S10. HSQC ($(CD_3)_2SO$, 600 MHz) of compound **2**

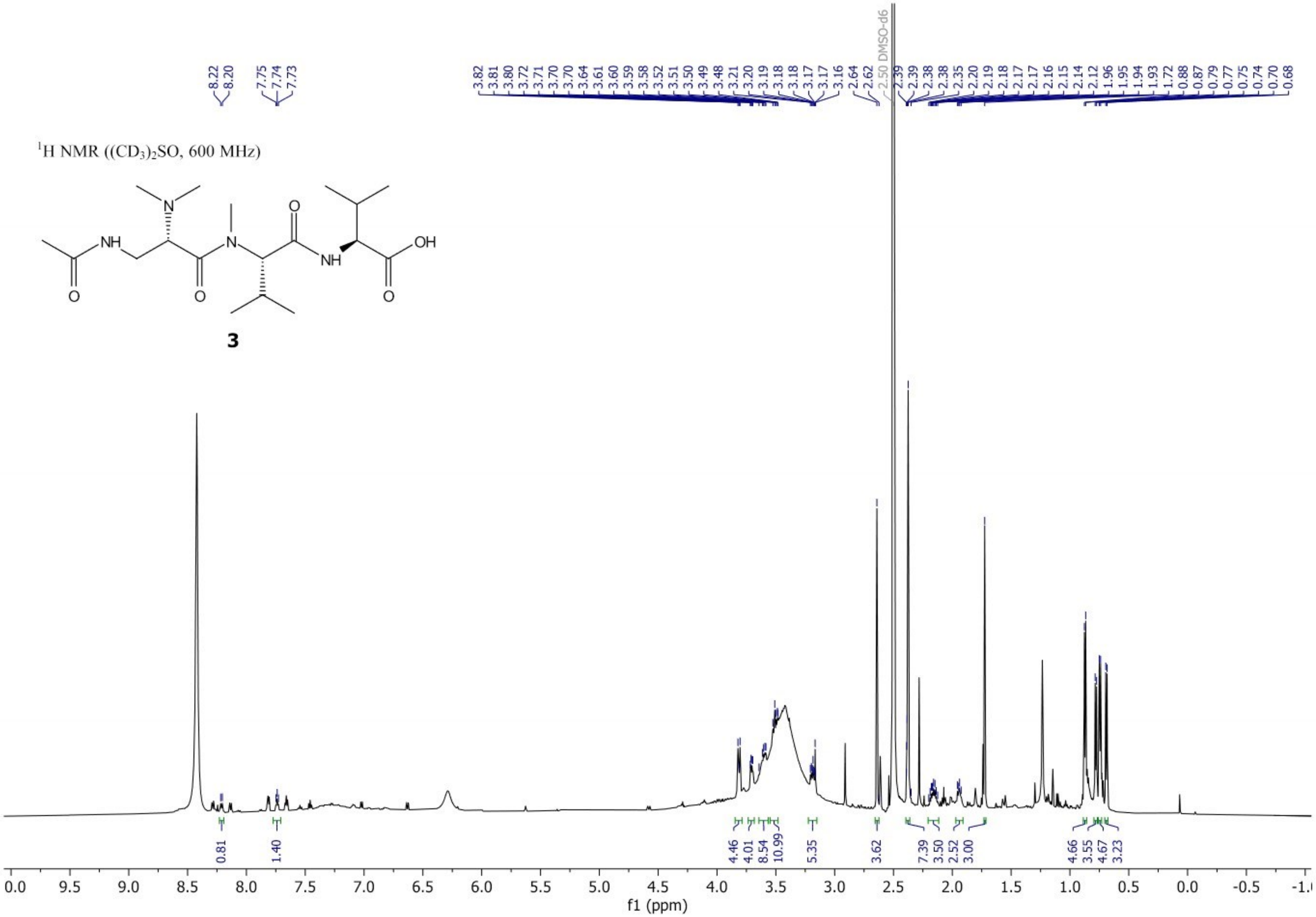

Figure S11. ^{1}H NMR ($(CD_3)_2SO$, 600 MHz) of compound **3**

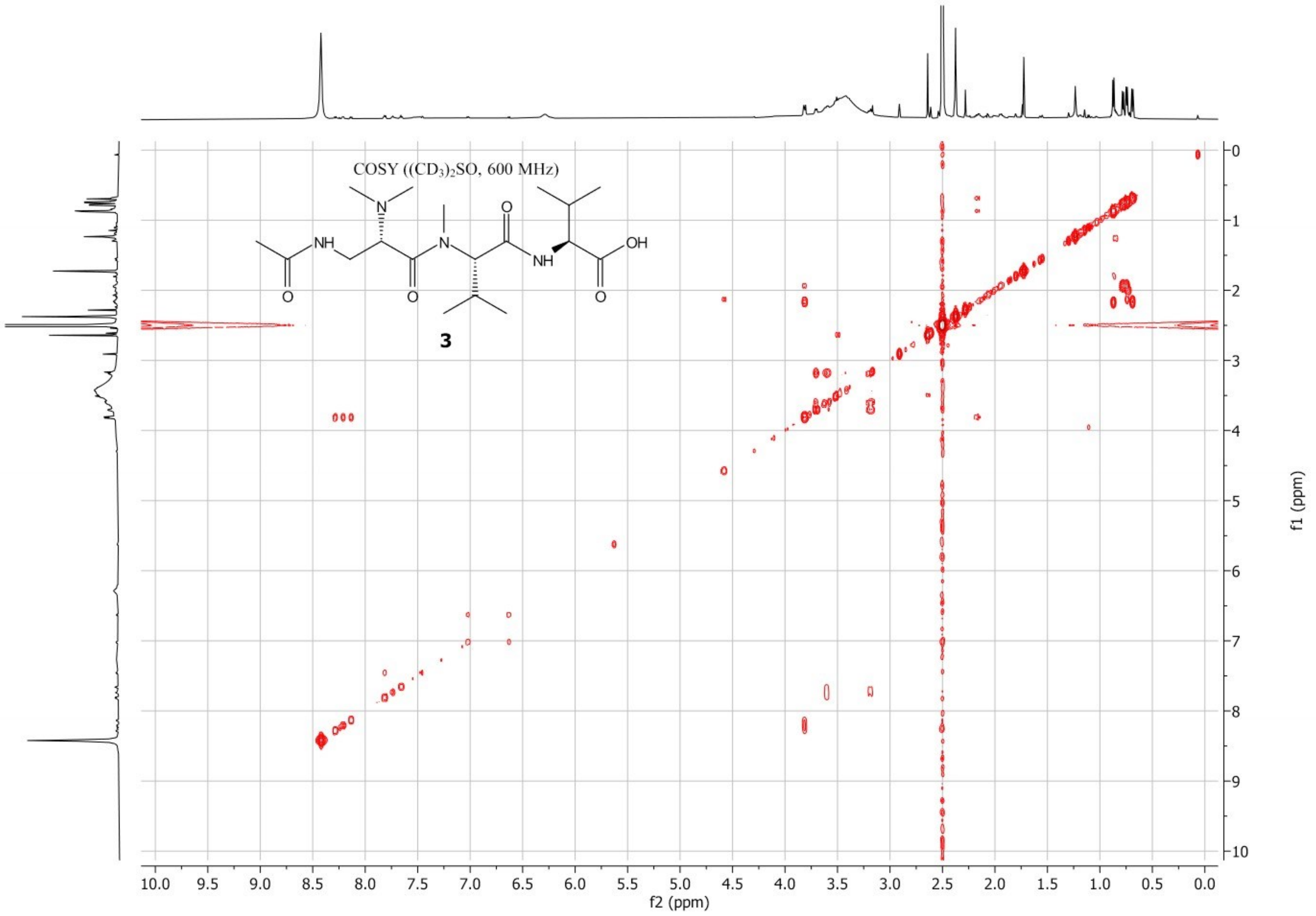

Figure S12. COSY ($(CD_3)_2SO$, 600 MHz) of compound **3**

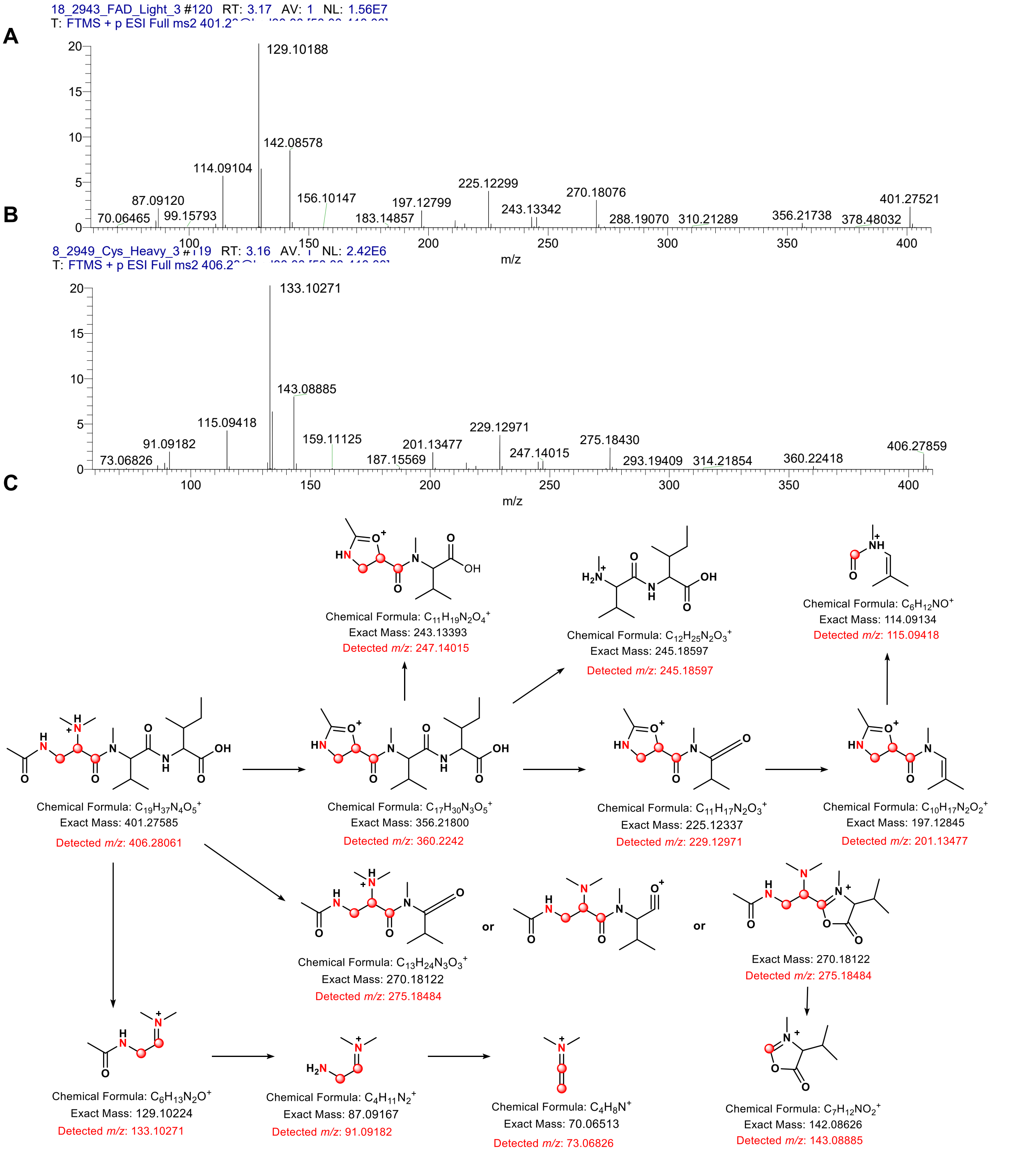

Figure S13. A) MSMS spectra of fusadapamide A. B) MSMS spectra of fusadapamide A incorporating isotopically labeled Dap (+5 Da, *m/z* 406.28) C) Proposed MSMS fragment structures from parent ion *m/z* 406.28, with heavy isotope C and N labeled in red. "Exact Mass" denotes fragment *m/z* from spectra in panel A), "Detected m/z" (red text) shows fragment *m/z* from spectra in panel B).

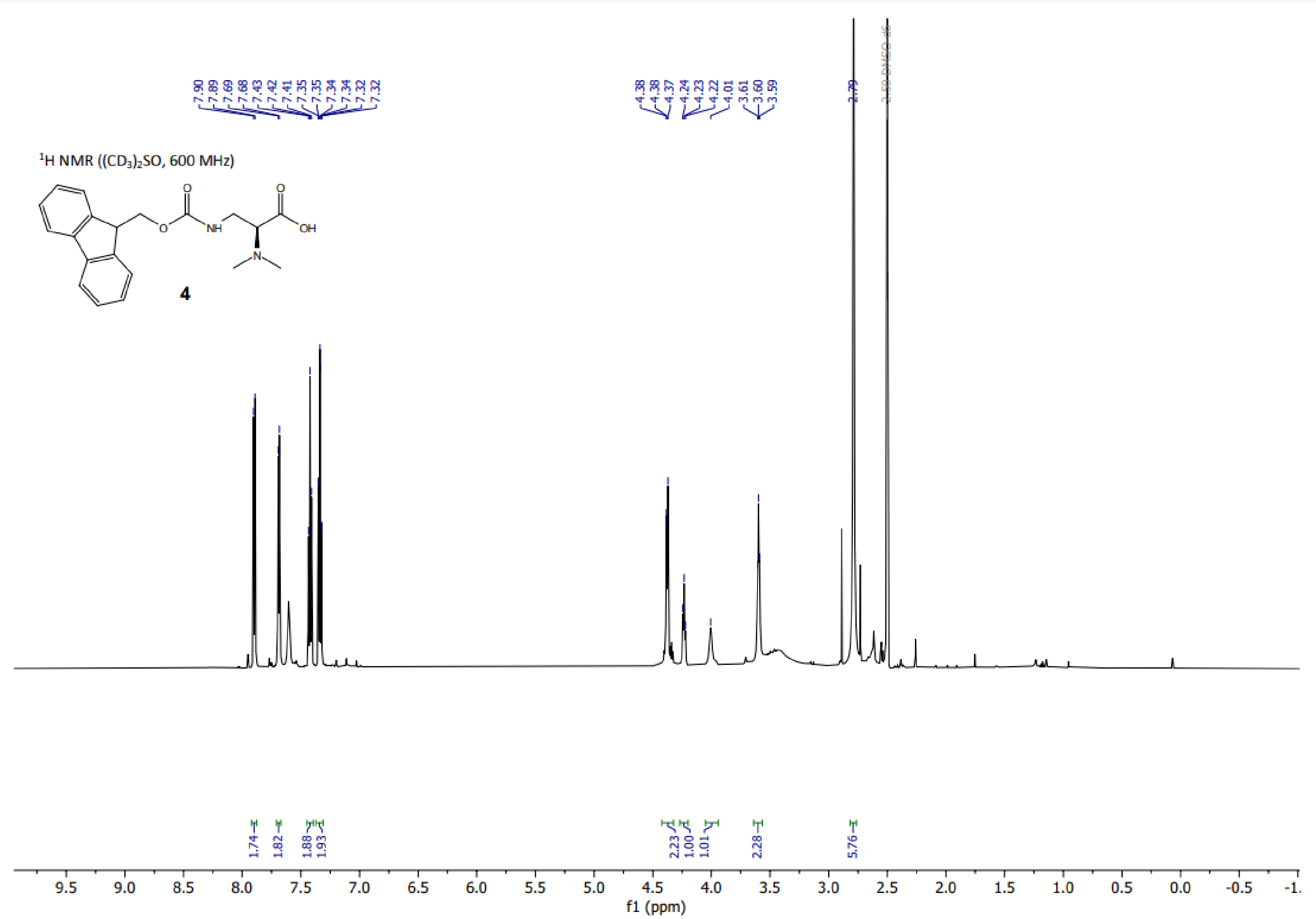

Figure S14. ^{1}H NMR ((CD$_3$)$_2$SO, 600 MHz) of compound **4**

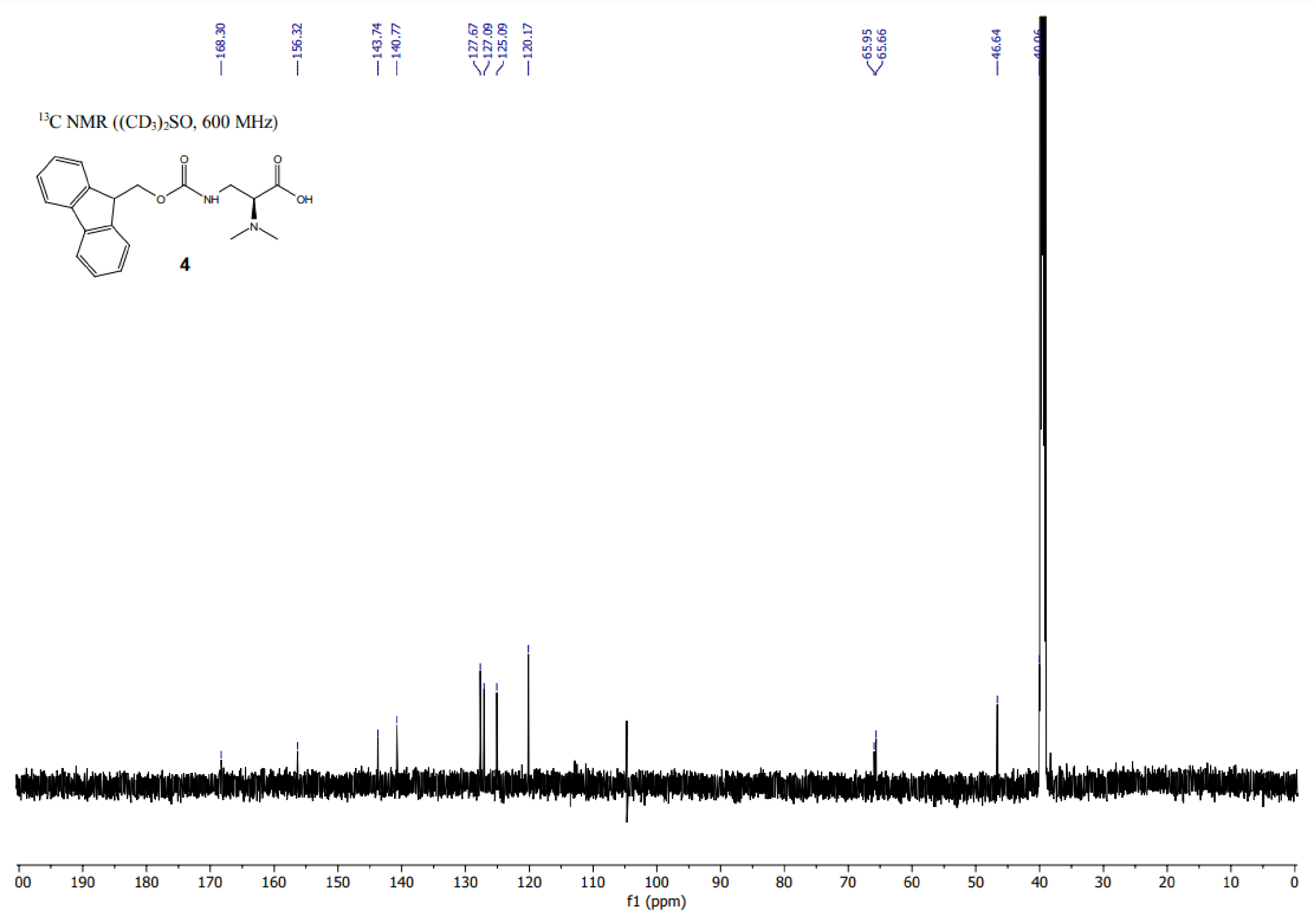

Figure S15. ^{13}C NMR ($(CD_3)_2SO$, 600 MHz) of compound **4**

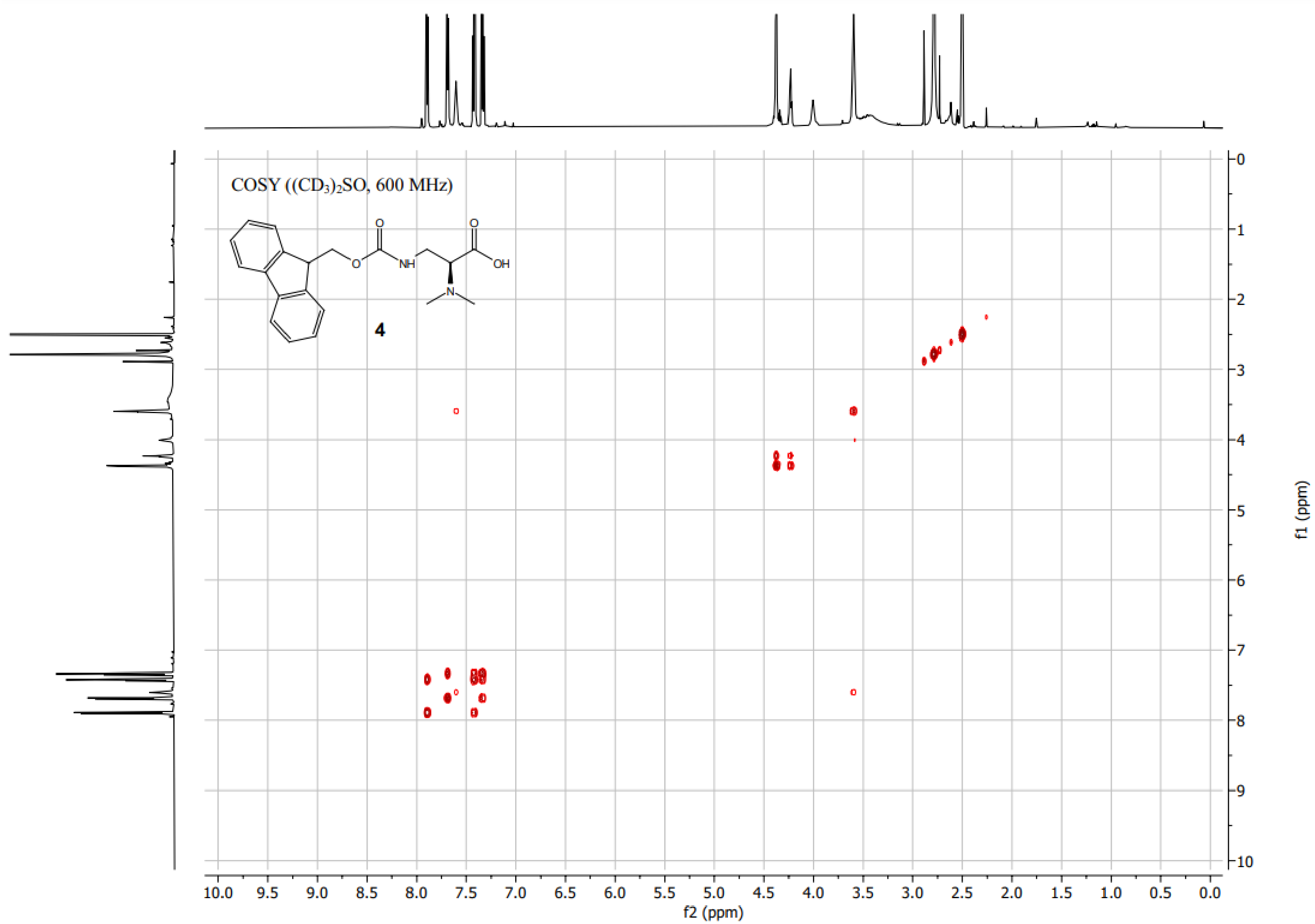

Figure S16. COSY ($(CD_3)_2SO$, 600 MHz) of compound **4**

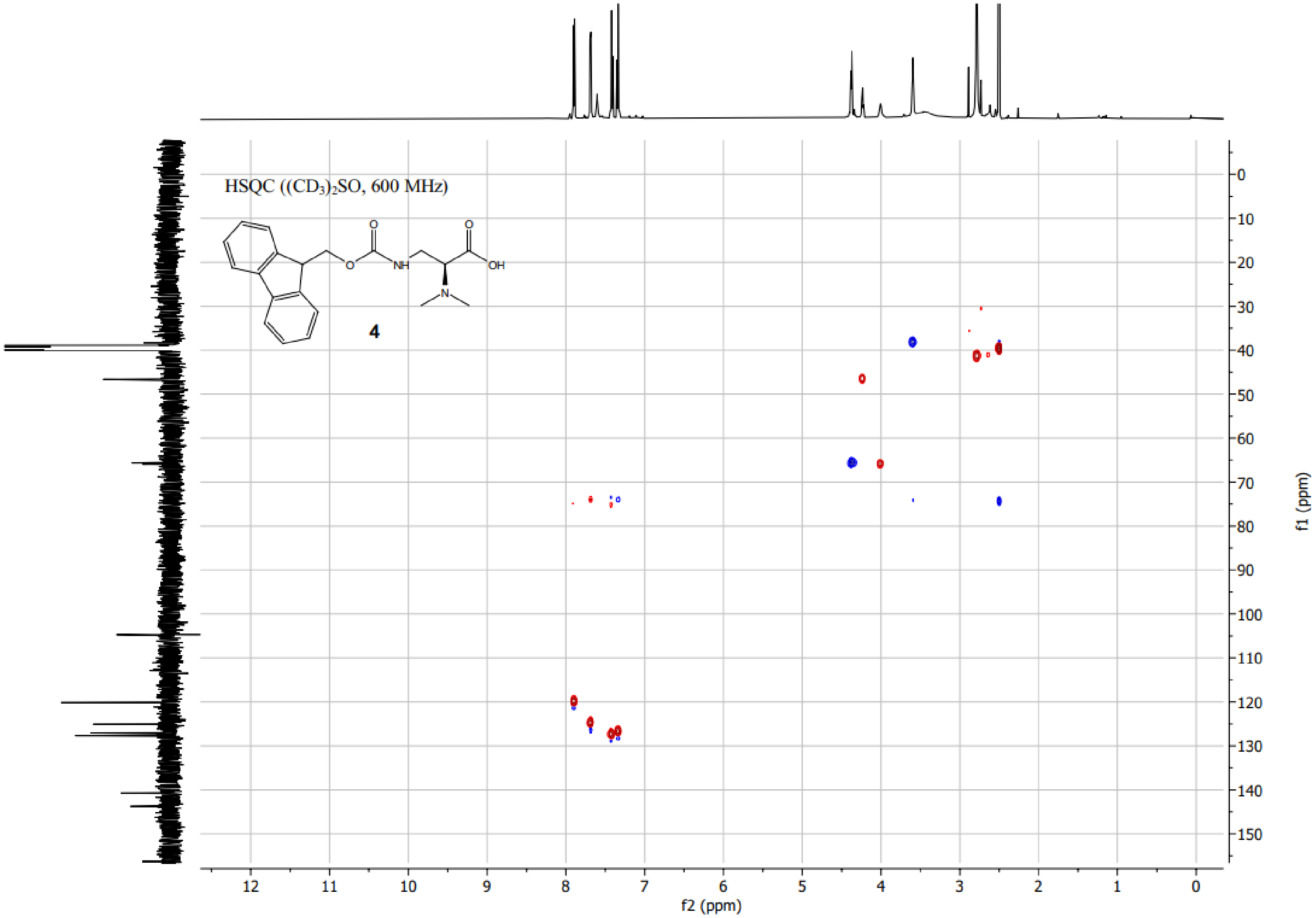

Figure S17. HSQC ($(CD_3)_2SO$, 600 MHz) of compound **4**

α-N-dimethyl-Dap (Fmoc protected) (4) (^{1}H NMR ((CD_3)$_2$SO, 600 MHz) δ 7.90 (d, *J* = 7.4 Hz, 2H), 7.69 (d, *J* = 7.5 Hz, 2H), 7.42 (t, *J* = 7.4 Hz, 2H), 7.34 (t, *J* = 1.2 Hz, 2H), 4.42 – 4.34 (m, 2H), 4.23 (t, *J* = 6.7 Hz, 1H), 4.01 (broad, 1H), 3.60 (t, *J* = 5.7 Hz, 2H), 2.79 (s, 6H)

^{13}C NMR ((CD_3)$_2$SO, 151 MHz) δ 168.3, 156.3, 143.7, 140.8, 127.7, 127.1, 125.1, 120.2, 66.0, 65.7, 46.6, 40.1.

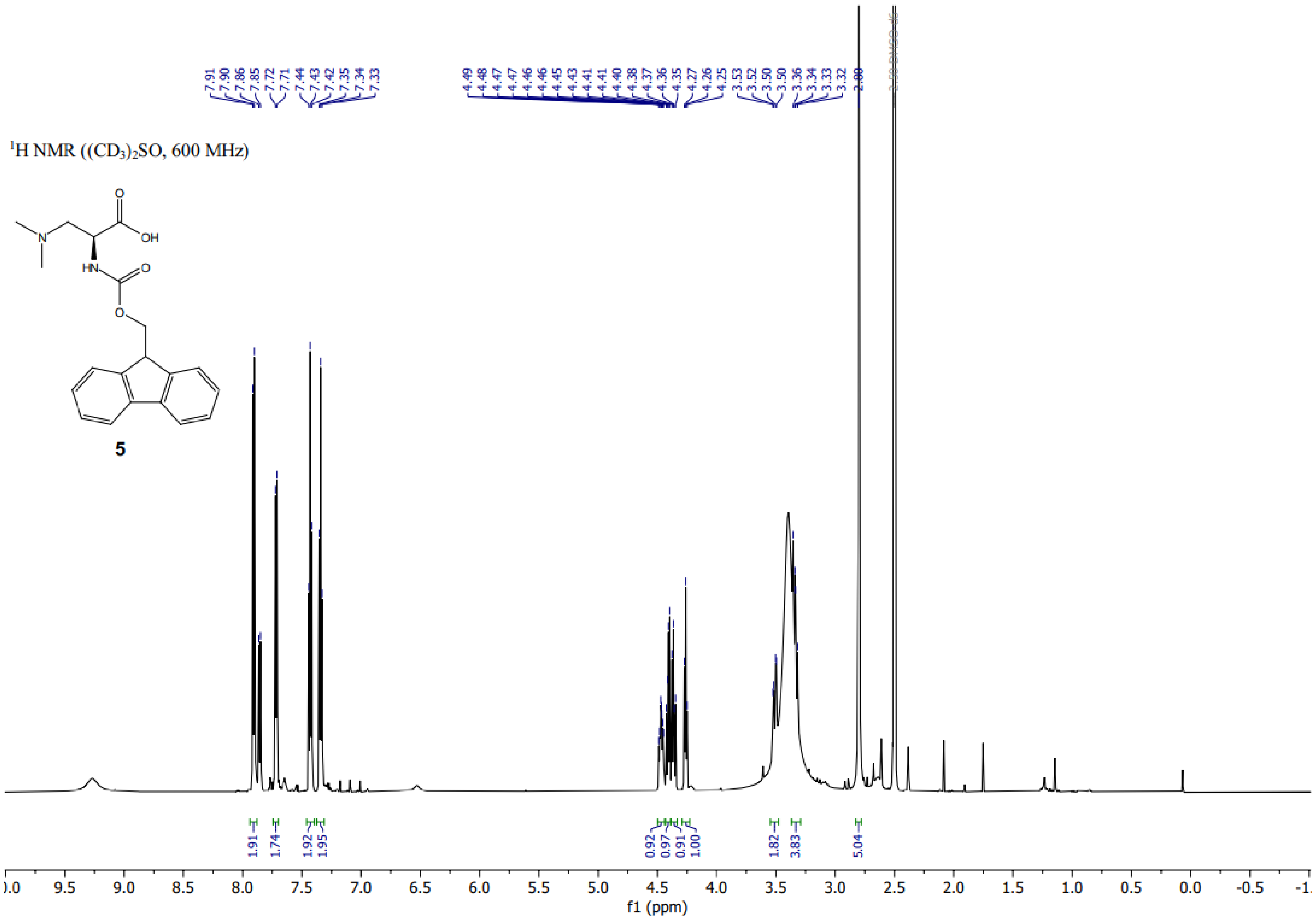

Figure S18. ^{1}H NMR ($(CD_3)_2SO$, 600 MHz) of compound **5**

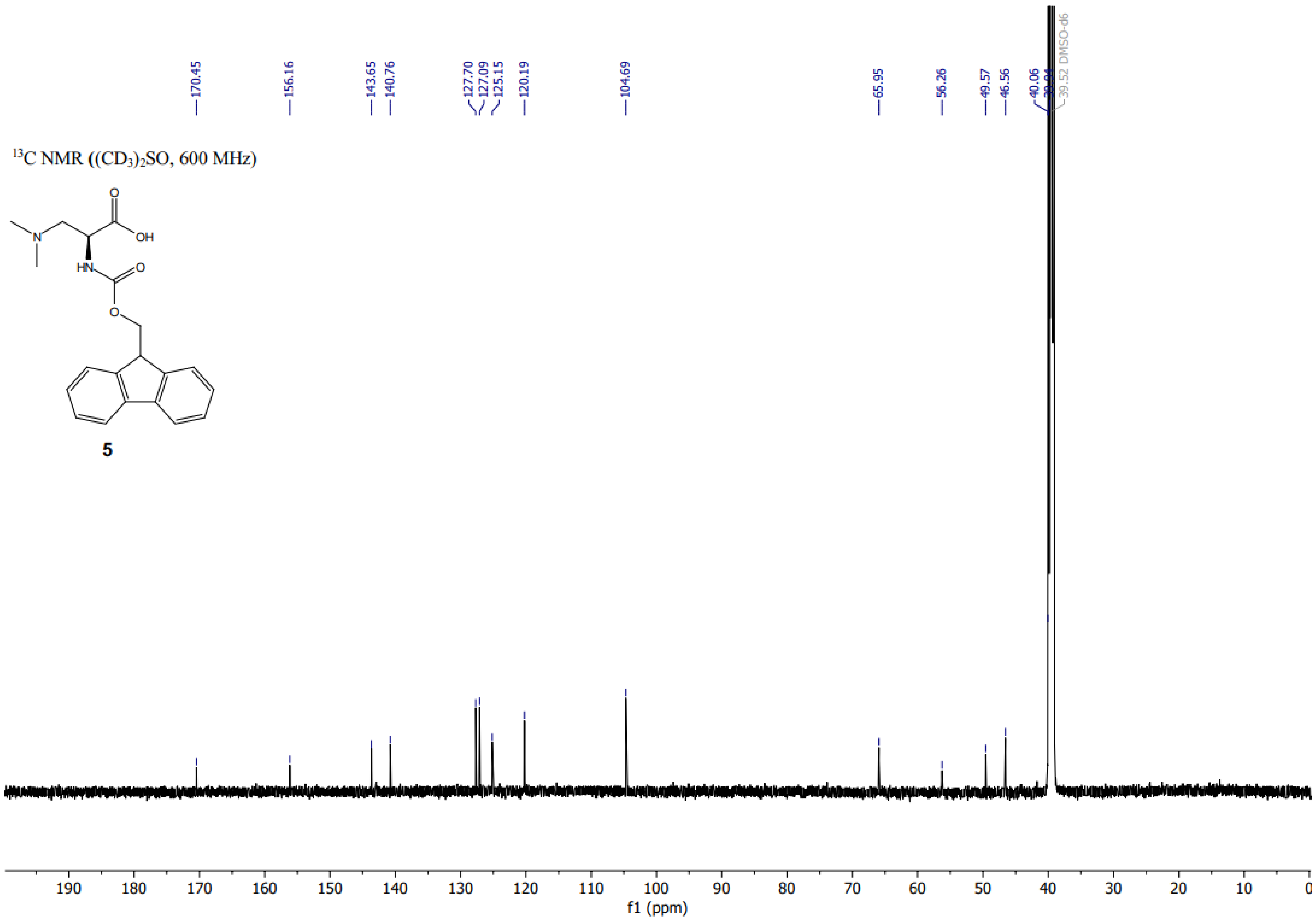

Figure S19. ^{13}C NMR ((CD_3)$_2$SO, 600 MHz) of compound **5**

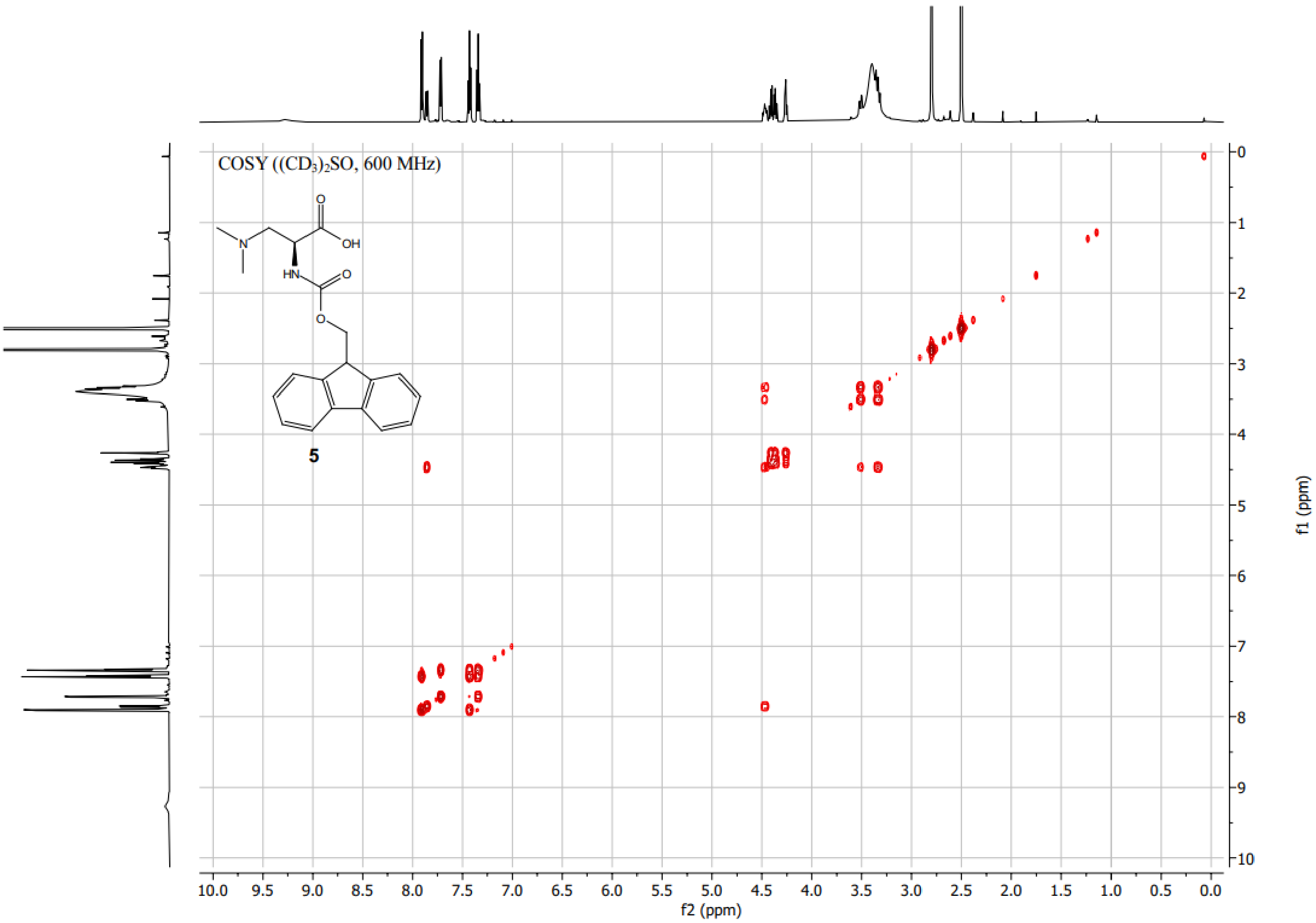

Figure S20. COSY ($(CD_3)_2SO$, 600 MHz) of compound **5**

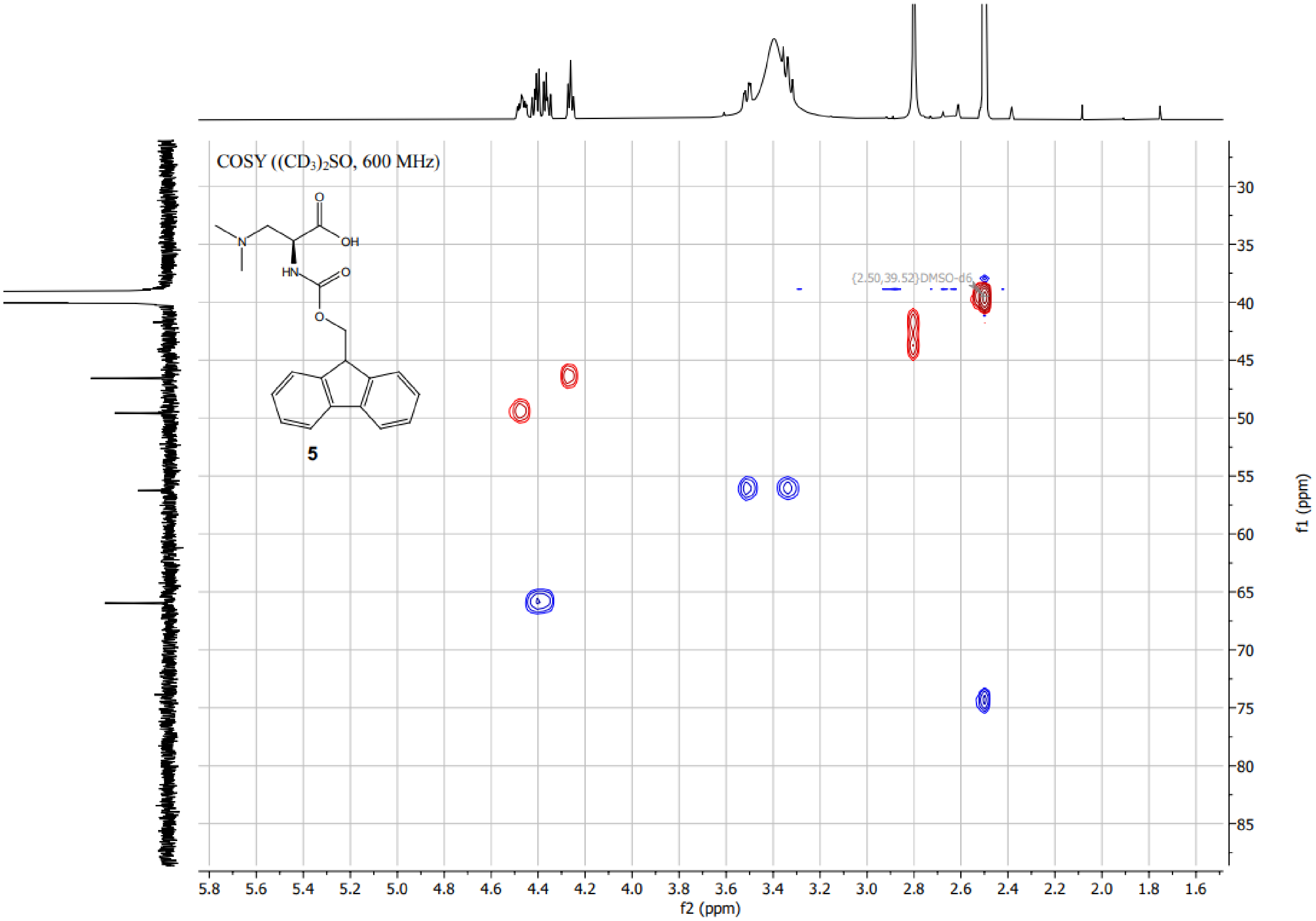

Figure S21. HSQC (($(CD_3)_2SO$, 600 MHz) of compound **5**

β-N-dimethyl-Dap (Fmoc protected) (5) (^{1}H NMR ($(CD_3)_2SO$, 600 MHz) δ 7.91 (d, *J* = 7.5 Hz, 2H), 7.72 (d, *J* = 7.5 Hz, 2H), 7.43 (t, *J* = 7.4 Hz, 2H), 7.34 (t, *J* = 7.4 Hz, 2H), 4.47 (td, *J* = 9.3, 4.1 Hz, 1H), 4.41 (dd, *J* = 10.6, 7.0 Hz, 1H), 4.36 (dd, *J* = 10.6, 6.7 Hz, 1H), 4.26 (t, *J* = 6.8 Hz, 1H), 3.51 (dd, *J* = 13.3, 4.2 Hz, 1H), 3.34 (dd, *J* = 13.2, 10.6 Hz, 1H), 2.80 (s, 6H).

^{13}C NMR ($(CD_3)_2SO$, 151 MHz) δ 170.5, 156.2, 143.7, 140.8, 127.7, 127.1, 125.2, 120.2, 66.0, 56.3, 49.6, 46.6, 40.1,

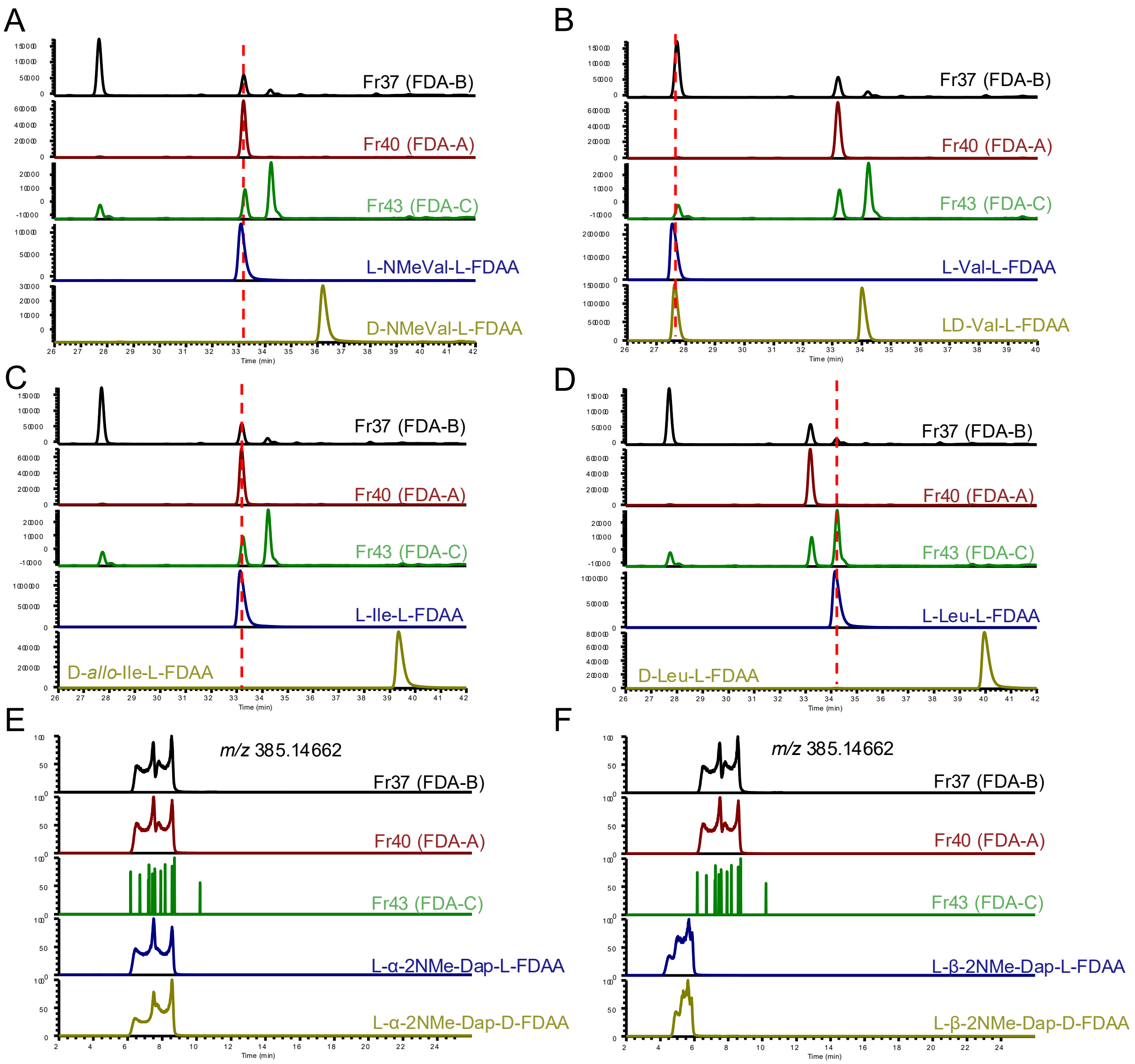

Figure S22. LC/MS chromatrograms of hydrolyzed and FDAA-derivatized HPLC fractions containing fusadapamides (FDA) A, B and C, compared to derivatized amino acid standards. **A-D)** Comparison to derivated commercial standards (RT 26 - 42 minutes, λ=340nm). Dotted red lines indicate location of peaks with *m/z* matching relevant derivatized standards. **E-F)** Comparison to synthesized N-dimethyl diaminopropionic acid (Dap) standards (RT 2 – 12 minutes, *m/z* 385.14662). Dual peaks suggest racemization of 2NMe-Dap during hydrolysis.

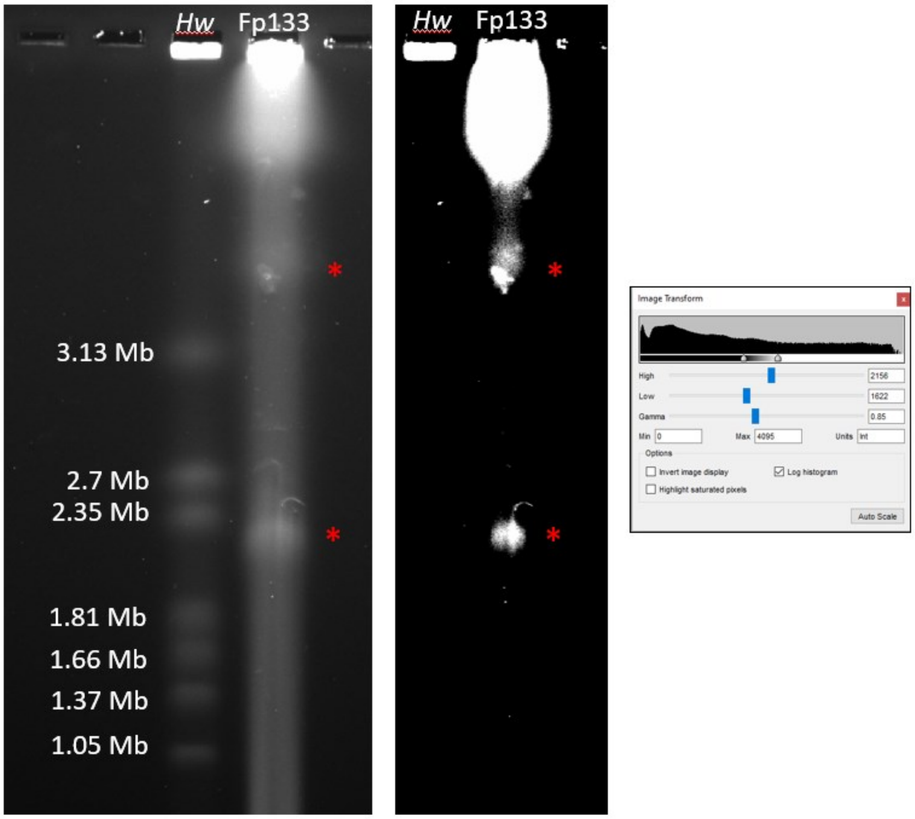

4

Figure S23. CHEF gel confirmation of small chromosome sizes. DNA visualized by ethidium bromide staining. Abbreviation *Hw* = *Hansenula wingeii* ladder. Red asterisks denote position of bands in *Fp*133 lane, corresponding to the genomic assembly-based predicted lengths of Chr5 (upper band, predicted length = 3.80 Mb) and Chr6 (lower band, predicted length = 2.08 Mb). Image on left is the unedited gel image with overlaid chromosome sizes of ladder. Image on right is manipulated to ‘flatten’ the image, exaggerating the putative chromosomal bands in the Fp133 lane (manipulation parameters displayed in inset box).

Table S1. Nanopore sequencing and assembly chromosome statistics for *Fp*133.

ID	Length (bp)	Genes/Mb	Repeat DNA(%)	RIP(%)
Chr1	12,048,428	331	8.1	3.95
Chr2	9,813,843	343	12.5	3.56
Chr3	8,641,347	343	12.8	3.39
Chr4	8,526,295	321	9.2	3.13
Chr5	3,800,058	251	59.8	0.18
Chr6	2,080,721	265	60.1	0.12

Nanopore stats	Fp133
# reads	206,801
longest read	219,176
median length	14,255
mean length	21,779
mean qscore	10.8
N50 (read length)	39,071
total bases (Gb)	4.5
mean coverage	94X

Table S2: List of *F. poae* isolates screened and results of PCR screening for *APS1* (apicidin NRPS) and *fda1* (fusadapamide NRPS) sequences.

Code	Year	CCFC Accession DAOMC #	Morden Research Centre ID	Province	Country	Crop	*aps1*	*fda1*	Associated reference
Fp001	2006	252342	-	Ontario	Canada	Barley	-	-	Witte et al. 2021
Fp002	2006	-	-	Ontario	Canada	Wheat	-	-	Witte et al. 2021
Fp003	2006	-	-	Ontario	Canada	Wheat	-	-	Witte et al. 2021
Fp004	2009	-	-	Ontario	Canada	Barley	-	-	Witte et al. 2021
Fp005	2010	-	-	Ontario	Canada	Barley	-	-	Witte et al. 2021
Fp006	2010	-	-	Ontario	Canada	Barley	-	-	Witte et al. 2021
Fp007	2010	-	-	Ontario	Canada	Barley	-	-	Witte et al. 2021
Fp008	2010	-	-	Ontario	Canada	Barley	-	-	Witte et al. 2021
Fp009	2010	-	-	Ontario	Canada	Barley	-	-	Witte et al. 2021
Fp010	2010	-	-	Ontario	Canada	Barley	-	-	Witte et al. 2021
Fp011	2010	-	-	Ontario	Canada	Barley	+	-	Witte et al. 2021
Fp012	2010	-	-	Ontario	Canada	Barley	-	-	Witte et al. 2021
Fp013	2010	252343	-	Ontario	Canada	Barley	+	-	Witte et al. 2021
Fp014	2010	-	-	Ontario	Canada	Barley	-	-	Witte et al. 2021
Fp015	2010	-	-	Ontario	Canada	Barley	-	-	Witte et al. 2021
Fp016	2010	252208	-	Ontario	Canada	Barley	-	-	Witte et al. 2021
Fp017	2010	-	-	Ontario	Canada	Barley	-	-	Witte et al. 2021
Fp018	2010	-	-	Ontario	Canada	Oat	-	-	Witte et al. 2021
Fp019	2010	-	-	Ontario	Canada	Oat	-	-	Witte et al. 2021
Fp020	2010	-	-	Ontario	Canada	Oat	-	-	Witte et al. 2021
Fp021	2010	252209	-	Ontario	Canada	Oat	-	-	Witte et al. 2021
Fp022	2010	-	-	Ontario	Canada	Oat	-	-	Witte et al. 2021
Fp023	2010	-	-	Ontario	Canada	Oat	+	+	Witte et al. 2021
Fp024	2010	252210	-	Ontario	Canada	Oat	-	-	Witte et al. 2021
Fp025	2010	-	-	Ontario	Canada	Oat	-	-	Witte et al. 2021
Fp026	2010	252211	-	Ontario	Canada	Oat	-	-	Witte et al. 2021
Fp027	2010	-	-	Ontario	Canada	Oat	-	-	Witte et al. 2021
Fp028	2010	-	-	Ontario	Canada	Oat	-	-	Witte et al. 2021
Fp029	2010	252212	-	Ontario	Canada	Oat	-	-	Witte et al. 2021
Fp030	2010	252213	-	Ontario	Canada	Oat	-	-	Witte et al. 2021
Fp031	2010	-	-	Ontario	Canada	Oat	-	-	Witte et al. 2021
Fp032	2010	-	-	Ontario	Canada	Oat	-	-	Witte et al. 2021
Fp033	2010	252214	-	Ontario	Canada	Oat	-	-	Witte et al. 2021
Fp034	2010	252215	-	Ontario	Canada	Oat	-	-	Witte et al. 2021
Fp035	2010	252216	-	Ontario	Canada	Oat	-	-	Witte et al. 2021
Fp036	2010	-	-	Ontario	Canada	Wheat	-	-	Witte et al. 2021

Fp037	2010	-	-	Ontario	Canada	wheat	-	-	Witte et al. 2021
Fp038	2010	252217	-	Ontario	Canada	Wheat	-	-	Witte et al. 2021
Fp039	2012	252218	-	Ontario	Canada	Barley	-	-	Witte et al. 2021
Fp040	2012	-	-	Ontario	Canada	Barley	-	-	Witte et al. 2021
Fp041	2012	-	-	Ontario	Canada	Barley	-	-	Witte et al. 2021
Fp042	2012	252219	-	Ontario	Canada	Barley	-	-	Witte et al. 2021
Fp043	2012	-	-	Ontario	Canada	Barley	-	-	Witte et al. 2021
Fp044	2012	252220	-	Ontario	Canada	Barley	-	-	Witte et al. 2021
Fp045	2012	-	-	Ontario	Canada	Barley	-	-	Witte et al. 2021
Fp046	2012	-	-	Ontario	Canada	Barley	-	-	Witte et al. 2021
Fp047	2012	-	-	Ontario	Canada	Barley	-	-	Witte et al. 2021
Fp048	2012	-	-	Ontario	Canada	Barley	-	-	Witte et al. 2021
Fp049	2012	252221	-	Ontario	Canada	Barley	-	-	Witte et al. 2021
Fp050	2012	252222	-	Ontario	Canada	Barley	-	-	Witte et al. 2021
Fp051	2012	252223	-	Ontario	Canada	Barley	-	-	Witte et al. 2021
Fp052	2012	-	-	Ontario	Canada	Barley	-	-	Witte et al. 2021
Fp053	2012	-	-	Ontario	Canada	Barley	-	-	Witte et al. 2021
Fp054	2012	-	-	Ontario	Canada	Barley	-	-	Witte et al. 2021
Fp055	2012	-	-	Ontario	Canada	Barley	-	-	Witte et al. 2021
Fp056	2012	-	-	Ontario	Canada	Barley	-	-	Witte et al. 2021
Fp057	2012	-	-	Ontario	Canada	Barley	-	-	Witte et al. 2021
Fp058	2012	-	-	Ontario	Canada	Barley	+	-	Witte et al. 2021
Fp059	2012	252224	-	Ontario	Canada	Barley	-	-	Witte et al. 2021
Fp060	2012	-	-	Ontario	Canada	Barley	-	-	Witte et al. 2021
Fp061	2012	-	-	Ontario	Canada	Barley	-	-	Witte et al. 2021
Fp062	2012	-	-	Ontario	Canada	Barley	-	-	Witte et al. 2021
Fp063	2012	252225	-	Ontario	Canada	Barley	-	-	Witte et al. 2021
Fp064	2012	-	-	Ontario	Canada	Barley	-	-	Witte et al. 2021
Fp065	2012	252226	-	Ontario	Canada	Oat	-	-	Witte et al. 2021
Fp066	2012	252227	-	Ontario	Canada	Oat	-	-	Witte et al. 2021
Fp067	2013	-	-	Ontario	Canada	Oat	-	-	Witte et al. 2021
Fp068	2012	-	-	Ontario	Canada	Oat	-	-	Witte et al. 2021
Fp069	2012	-	-	Ontario	Canada	Oat	-	-	Witte et al. 2021
Fp070	2012	-	-	Ontario	Canada	Oat	-	-	Witte et al. 2021
Fp071	2012	-	-	Ontario	Canada	Oat	-	-	Witte et al. 2021
Fp072	2012	252228	-	Ontario	Canada	Oat	+	-	Witte et al. 2021
Fp073	2012	252229	-	Ontario	Canada	Oat	-	-	Witte et al. 2021
Fp074	2012	-	-	Ontario	Canada	Oat	-	-	Witte et al. 2021
Fp075	2012	-	-	Ontario	Canada	Oat	-	-	Witte et al. 2021
Fp076	2012	252230	-	Ontario	Canada	Wheat	-	-	Witte et al. 2021
Fp077	2012	-	-	Ontario	Canada	Wheat	-	-	Witte et al. 2021

Fp078	2012	252231	-	Ontario	Canada	Wheat	-	-	Witte et al. 2021
Fp079	2012	-	-	Ontario	Canada	Wheat	-	-	Witte et al. 2021
Fp080	2012	-	-	Ontario	Canada	Wheat	-	-	Witte et al. 2021
Fp081	2012	252232	-	Ontario	Canada	Wheat	-	-	Witte et al. 2021
Fp082	2013	-	-	Ontario	Canada	Barley	-	-	Witte et al. 2021
Fp083	2013	-	-	Ontario	Canada	Barley	-	-	Witte et al. 2021
Fp084	2013	252233	-	Ontario	Canada	Barley	-	-	Witte et al. 2021
Fp085	2013	-	-	Ontario	Canada	Barley	-	-	Witte et al. 2021
Fp086	2013	252234	-	Ontario	Canada	wheat	-	-	Witte et al. 2021
Fp087	2014	252335	-	Saskatchewan	Canada	Oat	-	-	This study
Fp088	2014	252336	-	Saskatchewan	Canada	Oat	+	+	This study
Fp089	2014	-	-	Saskatchewan	Canada	Oat	-	-	This study
Fp090	2014	-	-	Quebec	Canada	Oat	-	-	Witte et al. 2021
Fp091	2015	-	-	Ontario	Canada	Barley	-	-	Witte et al. 2021
Fp092	2015	-	-	Ontario	Canada	Barley	-	-	Witte et al. 2021
Fp093	2015	-	-	Ontario	Canada	Barley	-	-	Witte et al. 2021
Fp094	2015	-	-	Ontario	Canada	Oat	-	-	Witte et al. 2021
Fp095	2015	-	-	Ontario	Canada	Oat	-	-	Witte et al. 2021
Fp096	2015	-	-	Ontario	Canada	Oat	-	-	Witte et al. 2021
Fp097	2015	-	-	Ontario	Canada	Oat	-	-	Witte et al. 2021
Fp098	2015	252235	-	Ontario	Canada	Wheat	-	-	Witte et al. 2021
Fp099	2015	-	-	Ontario	Canada	Wheat	-	-	Witte et al. 2021
Fp100	2015	-	-	Saskatchewan	Canada	Oat	-	-	This study
Fp101	2015	-	-	Saskatchewan	Canada	Oat	+	+	This study
Fp102	2015	-	-	Saskatchewan	Canada	Oat	-	-	This study
Fp103	2015	252337	-	Saskatchewan	Canada	Oat	+	+	This study
Fp104	2015	-	-	Saskatchewan	Canada	Oat	+	+	This study
Fp105	2015	252236	-	Ontario	Canada	Oat	-	-	Witte et al. 2021
Fp106	2015	-	-	Ontario	Canada	Oat	-	-	Witte et al. 2021
Fp107	2015	-	-	Ontario	Canada	Oat	+	+	Witte et al. 2021
Fp108	2015	252237	-	Ontario	Canada	Oat	-	-	Witte et al. 2021
Fp109	2015	-	-	Ontario	Canada	Oat	-	-	Witte et al. 2021
Fp110	2016	-	-	Ontario	Canada	Barley	-	-	Witte et al. 2021
Fp111	2016	-	-	Ontario	Canada	Barley	-	-	Witte et al. 2021
Fp112	2016	-	-	Ontario	Canada	Barley	-	-	Witte et al. 2021
Fp113	2016	-	-	Ontario	Canada	Barley	-	-	Witte et al. 2021
Fp114	2016	-	-	Ontario	Canada	Barley	-	-	Witte et al. 2021
Fp115	2016	-	-	Ontario	Canada	Barley	-	-	Witte et al. 2021
Fp116	2016	-	-	Ontario	Canada	Barley	-	-	Witte et al. 2021
Fp117	2016	-	-	Ontario	Canada	Barley	-	-	Witte et al. 2021
Fp118	2016	252238	-	Ontario	Canada	Oat	-	-	Witte et al. 2021

Fp119	2016	-	-	Ontario	Canada	Oat	-	-	Witte et al. 2021
Fp120	2016	-	-	Ontario	Canada	Oat	-	-	Witte et al. 2021
Fp121	2016	-	-	Ontario	Canada	Oat	-	-	Witte et al. 2021
Fp122	2016	252239	-	Ontario	Canada	Oat	-	-	Witte et al. 2021
Fp123	2016	-	-	Ontario	Canada	Wheat	-	-	Witte et al. 2021
Fp124	2016	-	-	Ontario	Canada	Wheat	+	-	Witte et al. 2021
Fp125	2016	252240	-	Ontario	Canada	Wheat	-	-	Witte et al. 2021
Fp126	2016	252338	-	Saskatchewan	Canada	Oat	-	-	This study
Fp127	2016	252339	-	Saskatchewan	Canada	Oat	-	-	This study
Fp128	2016	-	-	Saskatchewan	Canada	Oat	-	-	This study
Fp129	2016	-	-	Saskatchewan	Canada	Oat	-	-	This study
Fp130	2016	-	-	Saskatchewan	Canada	Oat	-	-	This study
Fp131	2016	-	-	Saskatchewan	Canada	Oat	-	-	This study
Fp132	2016	252340	-	Saskatchewan	Canada	Oat	-	-	This study
Fp133	2016	252341	-	Saskatchewan	Canada	Oat	+	+	This study
Fp134	2016	-	-	Saskatchewan	Canada	Oat	+	+	This study
Fp135	2016	-	-	Saskatchewan	Canada	Oat	-	-	This study
Fp136	2016	-	-	Saskatchewan	Canada	Oat	-	-	This study
Fp137	2016	-	-	Saskatchewan	Canada	Oat	-	-	This study
Fp138	2016	-	-	Saskatchewan	Canada	Oat	+	+	This study
Fp139	2016	-	-	Saskatchewan	Canada	Oat	-	-	This study
Fp140	2016	-	-	Saskatchewan	Canada	Oat	+	-	This study
Fp141	2016	-	-	Ontario	Canada	Oat	-	-	Witte et al. 2021
Fp142	2016	-	-	Ontario	Canada	Oat	-	-	Witte et al. 2021
Fp143	2016	-	-	Ontario	Canada	Oat	-	-	Witte et al. 2021
Fp144	2016	252241	-	Ontario	Canada	Oat	-	-	Witte et al. 2021
Fp145	2016	-	-	Ontario	Canada	Oat	-	-	Witte et al. 2021
Fp146	2016	252242	-	Ontario	Canada	Oat	+	-	Witte et al. 2021
Fp147	2016	-	-	Ontario	Canada	Oat	-	-	Witte et al. 2021
Fp148	2016	-	-	Ontario	Canada	Oat	-	-	Witte et al. 2021
Fp149	2016	-	-	Quebec	Canada	Oat	-	-	Witte et al. 2021
Fp150	2016	-	-	Quebec	Canada	Oat	-	-	Witte et al. 2021
Fp151	2016	-	-	Quebec	Canada	Oat	-	-	Witte et al. 2021
Fp152	2016	-	-	Quebec	Canada	Oat	-	-	Witte et al. 2021
Fp153	2016	-	-	Quebec	Canada	Oat	+	-	Witte et al. 2021
Fp154	2016	-	-	Quebec	Canada	Oat	-	-	Witte et al. 2021
Fp155	2016	252243	-	Quebec	Canada	Oat	-	-	Witte et al. 2021
Fp156	2016	-	-	Quebec	Canada	Oat	-	-	Witte et al. 2021
Fp157	2016	252244	-	Quebec	Canada	Oat	+	-	Witte et al. 2021
Fp158	2016	-	-	Quebec	Canada	Oat	-	-	Witte et al. 2021
Fp159	2016	-	-	Quebec	Canada	Oat	-	-	Witte et al. 2021

Fp160	2016	-	-	Quebec	Canada	Oat	-	-	Witte et al. 2021
Fp161	N/A	170319	-	Christchurch	New Zealand	poultry feed	-	-	Witte et al. 2021
Fp162	N/A	175360	-	Ontario	Canada	Maize	-	-	Witte et al. 2021
Fp163	1979	175362	-	Ontario	Canada	Maize	-	-	Witte et al. 2021
Fp164	1980	177426	-	Ontario	Canada	Wheat	-	-	Witte et al. 2021
Fp165	N/A	-	-	Manitoba	Canada	Wheat	-	-	Witte et al. 2021
Fp166	N/A	-	-	Manitoba	Canada	Wheat	-	-	Witte et al. 2021
Fp167	N/A	-	-	Manitoba	Canada	Wheat	-	-	Witte et al. 2021
Fp168	N/A	212321	-	Zurich	Switzerland	Corn	-	-	Witte et al. 2021
Fp169	N/A	212322	-	Zurich	Switzerland	Corn	-	-	Witte et al. 2021
Fp170	1985	-	-	Saskatchewan	Canada	Bromus inermis	+	+	This study
Fp171	1985	-	-	Saskatchewan	Canada	Bromus inermis	-	-	This study
Fp172	1985	-	-	Saskatchewan	Canada	Bromus inermis	-	-	This study
Fp173	1985	-	-	Saskatchewan	Canada	Bromus inermis	+	+	This study
Fp174	1991	215453	-	Ontario	Canada	Maize	-	-	Witte et al. 2021
Fp175	N/A	220671	-	N/A	Germany	Oat	-	-	Witte et al. 2021
Fp176	N/A	220672	-	N/A	Germany	Anthaxanthum odorarum	-	-	Witte et al. 2021
Fp177	N/A	220674	-	N/A	Japan	Wheat	-	-	Witte et al. 2021
Fp178	N/A	225733	-	Ontario	Canada	Maize	-	-	Witte et al. 2021
Fp180	1984	235670	-	Bloemfontein, Orange Free State	South Africa	Wheat	-	-	Witte et al. 2021
Fp181	N/A	235688	-	N/A	Spain		-	-	Witte et al. 2021
Fp182	1978	238070	-	Victoria	Australia	Dianthus caryophyllus	-	-	Witte et al. 2021
Fp183	1987	238076	-	Victoria	Australia	Brassica oleracea var. gemmifera	-	-	Witte et al. 2021
Fp185	2006	238878	-	Ontario	Canada		-	-	Witte et al. 2021
Fp186	2006	238879	-	Ontario	Canada		-	-	Witte et al. 2021
Fp187	2007	239526	-	Ontario	Canada	Barley	-	-	Witte et al. 2021
Fp188	2007	239527	-	Ontario	Canada	Wheat	-	-	Witte et al. 2021
Fp189	1981	-	-	Prince Edward Island	Canada	Wheat	-	-	Witte et al. 2021
Fp190	2017	-	-	Ontario	Canada	Barley	-	-	Witte et al. 2021
Fp191	2017	-	-	Ontario	Canada	Barley	-	-	Witte et al. 2021
Fp192	2017	-	-	Ontario	Canada	Barley	-	-	Witte et al. 2021
Fp193	2017	-	-	Ontario	Canada	Barley	-	-	Witte et al. 2021
Fp194	2017	-	-	Ontario	Canada	Barley	-	-	Witte et al. 2021
Fp195	2017	-	-	Ontario	Canada	Barley	-	-	Witte et al. 2021
Fp196	2017	-	-	Ontario	Canada	Barley	-	-	Witte et al. 2021
Fp197	2017	-	-	Ontario	Canada	Barley	-	-	Witte et al. 2021

Fp198	2017	-	-	Ontario	Canada	Barley	+	-	Witte et al. 2021
Fp199	2017	-	-	Ontario	Canada	Barley	-	-	Witte et al. 2021
Fp200	2017	-	-	Ontario	Canada	Barley	+	-	Witte et al. 2021
Fp201	2017	-	-	Ontario	Canada	Barley	-	-	Witte et al. 2021
Fp202	2017	-	-	Ontario	Canada	Barley	-	-	Witte et al. 2021
Fp203	2017	-	-	Ontario	Canada	Barley	-	-	Witte et al. 2021
Fp204	2017	-	-	Ontario	Canada	Barley	-	-	Witte et al. 2021
Fp205	2017	-	-	Ontario	Canada	Barley	-	-	Witte et al. 2021
Fp206	2017	-	-	Ontario	Canada	Barley	-	-	Witte et al. 2021
Fp207	2017	-	-	Ontario	Canada	Barley	-	-	Witte et al. 2021
Fp208	2017	-	-	Ontario	Canada	Barley	-	-	Witte et al. 2021
Fp209	2017	-	-	Ontario	Canada	Barley	-	-	Witte et al. 2021
Fp210	2017	-	-	Ontario	Canada	Barley	-	-	Witte et al. 2021
Fp211	2017	-	-	Ontario	Canada	Barley	-	-	Witte et al. 2021
Fp212	2017	-	-	Ontario	Canada	Barley	-	-	Witte et al. 2021
Fp213	2017	-	-	Ontario	Canada	Oat	-	-	Witte et al. 2021
Fp214	2017	-	-	Ontario	Canada	Oat	-	-	Witte et al. 2021
Fp215	2017	-	-	Ontario	Canada	Oat	-	-	Witte et al. 2021
Fp216	2017	-	-	Ontario	Canada	Oat	-	-	Witte et al. 2021
Fp217	2017	-	-	Ontario	Canada	Oat	-	-	Witte et al. 2021
Fp218	2017	-	-	Ontario	Canada	Oat	-	-	Witte et al. 2021
Fp219	2017	-	-	Ontario	Canada	Oat	-	-	Witte et al. 2021
Fp220	2017	-	-	Ontario	Canada	Oat	+	-	Witte et al. 2021
Fp221	2017	-	-	Ontario	Canada	Oat	-	-	Witte et al. 2021
Fp222	2017	-	-	Ontario	Canada	Oat	-	-	Witte et al. 2021
Fp223	2017	-	-	Ontario	Canada	Oat	-	-	Witte et al. 2021
Fp224	2017	-	-	Ontario	Canada	Oat	-	-	Witte et al. 2021
Fp225	2017	-	-	Ontario	Canada	Oat	-	-	Witte et al. 2021
Fp226	2017	-	-	Ontario	Canada	Oat	-	-	Witte et al. 2021
Fp227	2017	-	-	Ontario	Canada	Oat	-	-	Witte et al. 2021
Fp228	2017	-	-	Ontario	Canada	Oat	+	+	Witte et al. 2021
Fp229	2017	-	-	Ontario	Canada	Oat	-	-	Witte et al. 2021
Fp230	2017	-	-	Ontario	Canada	Oat	-	-	Witte et al. 2021
Fp231	2017	-	-	Ontario	Canada	Wheat	-	-	Witte et al. 2021
Fp232	2017	-	-	Ontario	Canada	Wheat	-	-	Witte et al. 2021
Fp233	2017	-	-	Ontario	Canada	Wheat	-	-	Witte et al. 2021
Fp234	2017	-	-	Ontario	Canada	Wheat	-	-	Witte et al. 2021
Fp235	2017	-	-	Ontario	Canada	Wheat	-	-	Witte et al. 2021
Fp236	2017	-	-	Ontario	Canada	Wheat	-	-	Witte et al. 2021
Fp237	2018	-	-	Ontario	Canada	Barley	-	-	This study†
Fp238	2018	-	-	Ontario	Canada	Barley	-	-	This study†

Fp239	2018	-	-	Ontario	Canada	Barley	-	-	This study†
Fp240	2018	-	-	Ontario	Canada	Barley	-	-	This study†
Fp241	2018	-	-	Ontario	Canada	Barley	-	-	This study†
Fp242	2018	-	-	Ontario	Canada	Barley	-	-	This study†
Fp243	2018	-	-	Ontario	Canada	Barley	-	-	This study†
Fp244	2018	-	-	Ontario	Canada	Oat	-	-	This study†
Fp245	2018	-	-	Ontario	Canada	Oat	-	-	This study†
Fp246	2018	-	-	Ontario	Canada	Oat	-	-	This study†
Fp247	2018	-	-	Ontario	Canada	Oat	+	-	This study†
Fp248	2018	-	-	Ontario	Canada	Oat	-	-	This study†
Fp249	2018	-	-	Ontario	Canada	Oat	-	-	This study†
Fp250	2018	-	-	Ontario	Canada	Oat	-	-	This study†
Fp252	2018	-	-	Ontario	Canada	Oat	-	-	This study†
Fp253	2018	-	-	Ontario	Canada	Oat	-	-	This study†
Fp254	2018	-	-	Ontario	Canada	Oat	-	-	This study†
Fp255	2018	-	-	Ontario	Canada	Oat	-	-	This study†
Fp256	2018	-	-	Ontario	Canada	Wheat	-	-	This study†
Fp257	2018	-	-	Ontario	Canada	Wheat	-	-	This study†
Fp258	2018	-	-	Ontario	Canada	Wheat	-	-	This study†
Fp260	2018	-	-	Ontario	Canada	Wheat	-	-	This study†
Fp262	2018	-	-	Quebec	Canada	Barley	-	-	This study†
Fp263	2018	-	-	Quebec	Canada	Barley	-	-	This study†
Fp264	2018	-	-	Quebec	Canada	Barley	-	-	This study†
Fp265	2018	-	-	Quebec	Canada	Oat	-	-	This study†
Fp266	2018	-	-	Quebec	Canada	Oat	-	-	This study†
Fp267	2018	-	-	Quebec	Canada	Oat	-	-	This study†
Fp268	2018	-	-	Quebec	Canada	Oat	-	-	This study†
Fp269	2018	-	-	Quebec	Canada	Oat	-	-	This study†
Fp270	2018	-	-	Quebec	Canada	Oat	-	-	This study†
Fp271	2018	-	-	Quebec	Canada	Oat	-	-	This study†
Fp272	2018	-	-	Quebec	Canada	Oat	-	-	This study†
Fp273	2018	-	-	Quebec	Canada	Wheat	-	-	This study†
Fp274	2018	-	-	Quebec	Canada	Wheat	-	-	This study†
Fp275	2018	-	-	Quebec	Canada	Wheat	-	-	This study†
Fp276	2018	-	-	Quebec	Canada	Wheat	-	-	This study†
Fp277	2018	-	-	Quebec	Canada	Wheat	-	-	This study†
Fp278	2018	-	-	Quebec	Canada	Wheat	-	-	This study†
Fp279	2018	-	-	Quebec	Canada	Wheat	-	-	This study†
Fp280	2018	-	-	Quebec	Canada	Wheat	+	-	This study†
Fp281	2018	-	-	Quebec	Canada	Wheat	+	-	This study†
Fp282	2018	-	-	Quebec	Canada	Wheat	+	-	This study†

Fp283	2018	-	-	Quebec	Canada	Wheat	-	-	This study†
Fp284	2018	-	-	Quebec	Canada	Wheat	-	-	This study†
Fp285	2018	-	-	Quebec	Canada	Wheat	-	-	This study†
Fp286	2018	-	-	Quebec	Canada	Wheat	-	-	This study†
Fp287	2018	-	MRC 399	Manitoba	Canada	Oat	-	-	This study
Fp288	2018	-	MRC 400	Manitoba	Canada	Oat	-	-	This study
Fp289	2018	-	MRC 401	Manitoba	Canada	Oat	-	-	This study
Fp290	2018	-	MRC 402	Manitoba	Canada	Oat	-	+	This study
Fp291	2018	-	MRC 403	Manitoba	Canada	Oat	-	-	This study
Fp292	2018	-	MRC 404	Manitoba	Canada	Oat	-	-	This study
Fp293	2018	-	MRC 405	Manitoba	Canada	Oat	-	-	This study
Fp294	2018	-	MRC 406	Manitoba	Canada	Oat	-	-	This study
Fp295	2018	-	MRC 407	Manitoba	Canada	Oat	-	-	This study
Fp296	2018	-	MRC 408	Manitoba	Canada	Oat	+	-	This study
Fp297	2018	-	MRC 409	Manitoba	Canada	Oat	-	-	This study
Fp298	2018	-	MRC 410	Manitoba	Canada	Oat	+	-	This study
Fp299	2018	-	MRC 411	Manitoba	Canada	Oat	-	-	This study
Fp300	2018	-	MRC 412	Manitoba	Canada	Oat	-	-	This study
Fp301	2018	-	MRC 413	Manitoba	Canada	Oat	+	-	This study
Fp302	2018	-	MRC 415	Manitoba	Canada	Oat	-	-	This study
Fp303	2018	-	MRC 416	Manitoba	Canada	Oat	-	-	This study
Fp304	2018	-	MRC 417	Manitoba	Canada	Oat	-	-	This study
Fp305	2018	-	MRC 418	Manitoba	Canada	Oat	-	-	This study
Fp306	2018	-	MRC 419	Manitoba	Canada	Oat	+	+	This study
Fp307	2018	-	MRC 420	Manitoba	Canada	Oat	-	-	This study
Fp308	2018	-	MRC 421	Manitoba	Canada	Oat	+	+	This study
Fp309	2018	-	MRC 422	Manitoba	Canada	Oat	+	+	This study
Fp310	2018	-	MRC 423	Manitoba	Canada	Oat	-	-	This study
Fp311	2018	-	MRC 424	Manitoba	Canada	Oat	-	-	This study
Fp312	2018	-	MRC 425	Manitoba	Canada	Oat	-	-	This study
Fp313	2018	-	MRC 426	Manitoba	Canada	Oat	-	-	This study
Fp314	2018	-	MRC 427	Manitoba	Canada	Oat	-	-	This study
Fp315	2018	-	MRC 428	Manitoba	Canada	Oat	-	-	This study
Fp316	2018	-	MRC 429	Manitoba	Canada	Oat	-	-	This study
Fp317	2018	-	MRC 430	Manitoba	Canada	Oat	-	-	This study
Fp318	2018	-	MRC 431	Manitoba	Canada	Oat	-	-	This study
Fp319	2018	-	MRC 432	Manitoba	Canada	Oat	-	-	This study
Fp320	2018	-	MRC 433	Manitoba	Canada	Oat	-	-	This study
Fp321	2018	-	MRC 434	Manitoba	Canada	Oat	-	-	This study
Fp322	2018	-	MRC 435	Manitoba	Canada	Oat	-	-	This study
Fp323	2018	-	MRC 436	Manitoba	Canada	Oat	-	-	This study

Fp324	2018	-	MRC 437	Manitoba	Canada	Oat	-	-	This study
Fp325	2018	-	MRC 438	Manitoba	Canada	Oat	+	+	This study
Fp326	2018	-	MRC 439	Manitoba	Canada	Oat	-	-	This study
Fp327	2018	-	MRC 440	Manitoba	Canada	Oat	+	+	This study
Fp328	2018	-	MRC 441	Manitoba	Canada	Oat	+	+	This study
Fp329	2018	-	MRC 442	Manitoba	Canada	Oat	-	-	This study
Fp330	2018	-	MRC 443	Manitoba	Canada	Oat	-	-	This study
Fp331	2018	-	MRC 444	Manitoba	Canada	Oat	-	nd	This study
Fp332	2018	-	MRC 445	Manitoba	Canada	Oat	-	-	This study
Fp333	2018	-	MRC 446	Manitoba	Canada	Oat	-	-	This study
Fp334	2018	-	MRC 447	Manitoba	Canada	Oat	-	-	This study
Fp335	2018	-	MRC 448	Manitoba	Canada	Oat	+	+	This study
Fp336	2018	-	MRC 449	Manitoba	Canada	Oat	-	-	This study
Fp337	2018	-	MRC 450	Manitoba	Canada	Oat	-	-	This study
Fp338	2018	-	MRC 451	Manitoba	Canada	Oat	-	-	This study
Fp339	2018	-	MRC 452	Manitoba	Canada	Oat	+	+	This study
Fp340	2018	-	MRC 453	Manitoba	Canada	Oat	-	-	This study
Fp341	2018	-	MRC 454	Manitoba	Canada	Oat	-	-	This study
Fp342	2018	-	MRC 455	Manitoba	Canada	Oat	-	-	This study
Fp343	2018	-	MRC 456	Manitoba	Canada	Oat	-	-	This study
Fp344	2018	-	MRC 457	Manitoba	Canada	Oat	-	-	This study
Fp345	2018	-	MRC 458	Manitoba	Canada	Oat	-	-	This study
Fp346	2018	-	MRC 459	Manitoba	Canada	Oat	-	-	This study
Fp347	2018	-	MRC 460	Manitoba	Canada	Oat	-	-	This study
Fp348	2018	-	MRC 461	Manitoba	Canada	Oat	-	-	This study
Fp349	2018	-	MRC 462	Manitoba	Canada	Oat	-	-	This study
Fp350	2019	-	MRC 621	Saskatchewan	Canada	Oat	+	+	This study
Fp351	2019	-	MRC 622	Saskatchewan	Canada	Oat	-	-	This study
Fp355	2019	-	MRC 629	Saskatchewan	Canada	Oat	-	-	This study
Fp357	2019	-	MRC 634	Manitoba	Canada	Oat	-	-	This study
Fp358	2019	-	MRC 635	Manitoba	Canada	Oat	-	-	This study
Fp360	2019	-	MRC 641	Manitoba	Canada	Oat	-	-	This study
Fp361	2019	-	MRC 642	Manitoba	Canada	Oat	+	+	This study
Fp362	2019	-	MRC 643	Manitoba	Canada	Oat	-	-	This study
Fp363	2019	-	MRC 644	Manitoba	Canada	Oat	-	-	This study
Fp364	2019	-	MRC 646	Manitoba	Canada	Oat	-	-	This study
Fp365	2019	-	MRC 647	Manitoba	Canada	Oat	-	-	This study
Fp366	2019	-	MRC 654	Manitoba	Canada	Oat	-	-	This study
Fp367	2019	-	MRC 655	Manitoba	Canada	Oat	+	-	This study
Fp369	2019	-	MRC 661	Saskatchewan	Canada	Oat	-	-	This study
Fp370	2019	-	MRC 665	Manitoba	Canada	Oat	-	-	This study

Fp371	2019	-	MRC 667	Manitoba	Canada	Oat	-	-	This study
Fp372	2019	-	MRC 669	Manitoba	Canada	Oat	-	-	This study
Fp373	2019	-	MRC 670	Saskatchewan	Canada	Oat	-	-	This study
Fp374	2019	-	MRC 671	Saskatchewan	Canada	Oat	-	-	This study
Fp375	2019	-	MRC 675	Manitoba	Canada	Oat	-	-	This study
Fp377	2019	-	MRC 681	Saskatchewan	Canada	Oat	-	-	This study
Fp378	2019	-	MRC 684	Manitoba	Canada	Oat	-	-	This study
Fp379	2019	-	MRC 686	Manitoba	Canada	Oat	-	-	This study
Fp380	2019	-	MRC 687	Saskatchewan	Canada	Oat	+	+	This study
Fp381	2019	-	MRC 688	Manitoba	Canada	Oat	-	-	This study
Fp382	2019	-	MRC 691	Manitoba	Canada	Oat	-	-	This study
Fp384	2019	-	MRC 692	Manitoba	Canada	Oat	+	-	This study
Fp386	2019	-	MRC 697	Manitoba	Canada	Oat	+	-	This study

Table S3. PCR primers used in this study

Gene	Primer name	Primer sequence (5' - 3')	Details
fda1	NRP FPOAC2_13301 F11939 (1938)	TCCCAAGACTTGTCGACGTG	within gene
FPOAC2_13301	NRP FPOAC2_13301 R12654 (1939)	GCTGCGATTTCGAGCTCAAG	
NRPS	Fp 2516F (1930)	GGGGGTTAATCACCTGGTGG	upstream F
	Fp17228R (1933)	CCGTACCGCCTATTCCAGAC	downstream R
fda2	FPOAC2_13302 F517 (1934)	TTTCACCGTTGCATGTGCTG	within gene
FPOAC2_13302	FPOAC2_13302 1259R (1935)	TCGCACCACTGACTACCAAC	
MFS transporter	FPOAC2_13302 _10F (1927)	TCCCCACTCTGTTTGGATCC	upstream F
	FPOAC2_13302 2503R (1928)	CTAGTTTGGCTACCCCGCAT	downstream R
fda3	FPOAC2_13303 128F (1944)	CTCCAAACGGCACTGATCCT	within gene
FPOAC2_13303	FPOAC2_13303 677R (1945)	AGTGAGAGTCTTCCGGTCGA	
FAD REDOX	FPOAC2_13303 upF (1948)	TATCCGGAGAGATGTCTACTCCC	upstream F
	FPOAC2_13303 downR (1949)	TGGAGCTGACTGATGTGCTT	downstream R
fda4	FPOAC2_13305 176F (1946)	GTGAAAGCTCTTCCCGGTGA	within genes
FPOAC2_13305	FPOAC2_13305 771R (1947)	CCAGTACCTGTGACACACCC	
Cysteine	FPOAC2_13305 upF (1950)	CACTTGCGGCCTGGGATATT	upstream F
synthase	FPOAC2_13305 dwnR (1951)	CGCCGAAAACGTGATTGTGT	downstream R
	fda4f	ATGGCTAGCATGGCAAAAAC CGAATTCCT	recombinant F with NheI restriction site
	fda4r	TTCGGATCCTTACTGCCTGA CATCAAGC	recombinant R with BamHI restriction site
fda5	FPOAC2_13306 intconfF	CGATGCCTTAAATCGCGCAA	within gene
FPOAC2_13306	FPOAC2_13306 intconfR	TCCCTGGCTCCACGATCATA	
GNAT	FPOAC2_13306 upF	TGAGTCGAGTCCAGCTACGA	upstream F
	FPOAC2_13306 dwnR	TGGGTCTGACATATTTGGT	downstream R
APS1	APS1 2115F (1940)	ATCGGCAATCTCCCGTCATC	within gene
FPOAC2_13359	APS1 2692R (1941)	GATTCGTTGCATCCACTCGC	
	APS1upstreamF (1942)	TGTTTGCTCTGTGTGACCGT	upstreamF
	APS1downstreamR (1943)	AGCCGGATCTTCCCAAGTTG	downstreamR
Hygromycin R.	1113 Hyg588U	AGCTGCGCCGATGGTTTCTACAA	within gene
	1114 Hyg588L	GCGCGTCTGCTGCTCCATACAA	
Geneticin R.	1393 nptIIF1	GGGCGCCCGGTTCTTTTTG	within gene
	1394 nptIIR1	ACACCCAGCCGGCCACAGTCG	
TEF1α	TEF1α fwd:	ATGGGTAAGGAGGAGAAGACT	within gene
	TEF1α rev:	GGAAGTACCAGTGATCATGTT	

Table S4. Protein ID, predicted PFAM domain annotations and averaged mRNA expression values (transcripts per million reads, TPM) from Fp133 cultured on MMK2 and YES agar media. Greyed rows with bolded letters were predicted to be relevant to fusadapamide biosynthesis in this study.

Protein ID	PFAM domains	E-value	Fp133 MMK2 2d - Mean TPM	Fp133 MMK2 6d - Mean TPM	Fp133 YES 2d - Mean TPM	Fp133 YES 6d - Mean TPM
FPOAC2_13297	Unknown	N/A	0.41	3.83	0.99	5.75
FPOAC2_13298	Unknown	N/A	1.08	1.00	0.87	1.65
FPOAC2_13299	Cysteine protease (PF00648.21)	1.40E-49	0.16	0.17	0.51	0.34
FPOAC2_13300	DUF4451 (PF14616.6)	3.00E-19	0.48	0.18	0.11	0.04
**Fda1**	**NRPS**	**N/A**	**21.67**	**81.71**	**27.88**	**153.37**
**Fda2**	**MFS transporter (PF07690.16)**	**1.70E-49**	**97.23**	**328.35**	**120.46**	**422.84**
**Fda3**	**FAD-dependent oxidoreductase (PF01266.24)**	**8.40E-28**	**74.96**	**60.53**	**80.10**	**61.96**
**Fda4**	**Cysteine synthase (PLP + RHOD domains)**	**1E-30 (PLP), 3.7E-06 (RHOD)**	**175.42**	**33.96**	**249.99**	**126.97**
**Fda5**	**N-acetyltransferase**	**2.90E-03**	**133.04**	**111.90**	**137.15**	**274.01**
FPOAC2_13307	Ors-D	4.50E-09	0.37	0.82	0.69	1.00
FPOAC2_13308	Unknown	N/A	2.00	2.70	2.37	1.94
FPOAC2_13309	NACHT, P-loop NTPase, WD-40 repeats	4E-70, 8.9E-09, 3.19E-05	10.18	32.22	11.03	41.13
FPOAC2_13310	Ors-D	2.51E-11	4.44	29.53	16.91	73.35

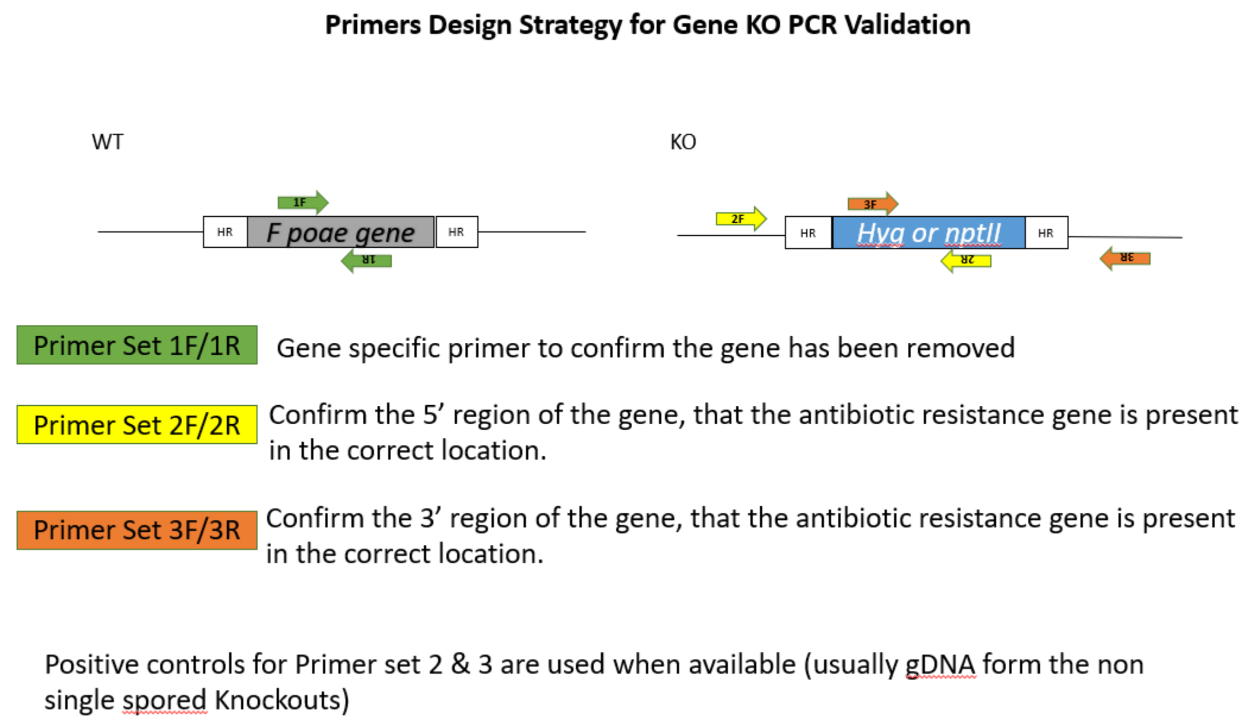

Figure S24. PCR primer design strategy for gene deletion validation.

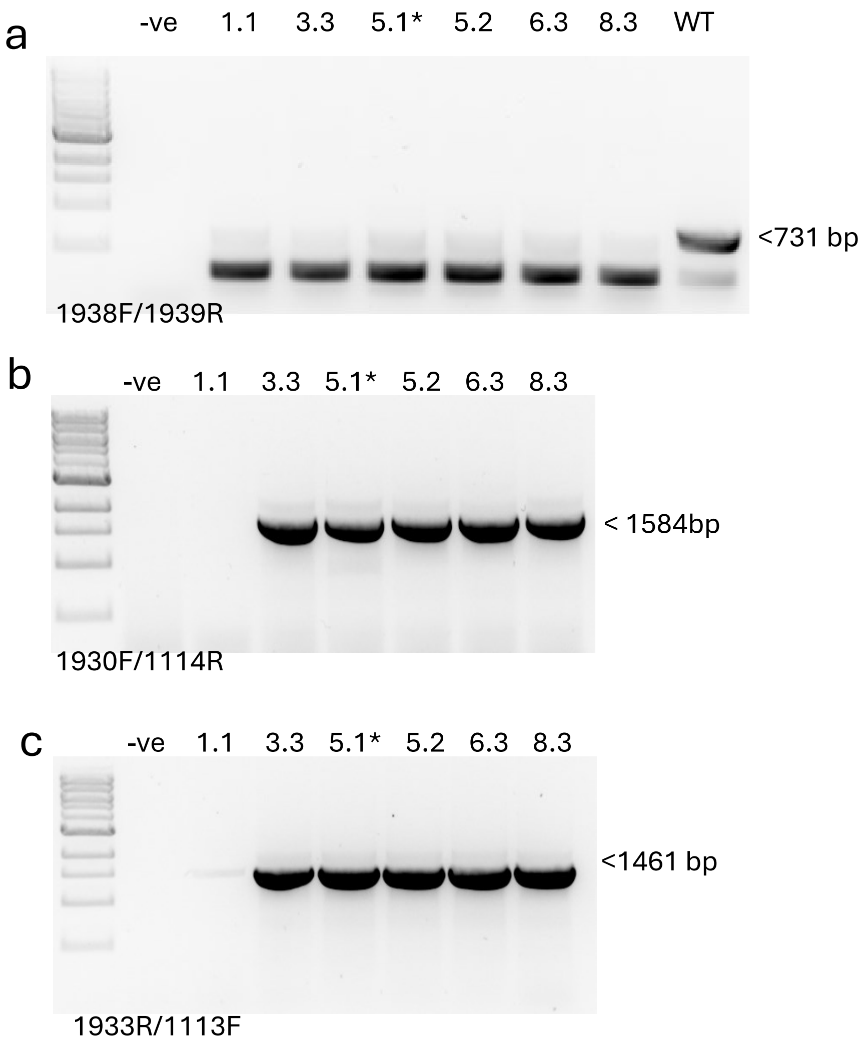

Figure S25. PCR validation of *Fp*133ΔFPOAC2_13301 (*fda1*) deletion transformants. Single spored isolate 5.1 (asterisks) was selected for metabolomics and *in planta* trials. **a)** The Primer set 1: 1938F/1939R amplifies 731bp region within the target gene. Present in WT, but absent in all KO clones. Low molecular weight band represents nonspecific binding (present in all strains). **b)** Primer set 2: PCR test for Hygromycin resistance gene 5' presence. In clone 1.1, gene is absent, but the 5'region is not showing expected product. All other clones displayed expected PCR product. **c)** Primer set 3: PCR test for Hygromycin resistance gene 3' presence. In Clone 1.1 a very faint product is displayed. All other clones have expected size.

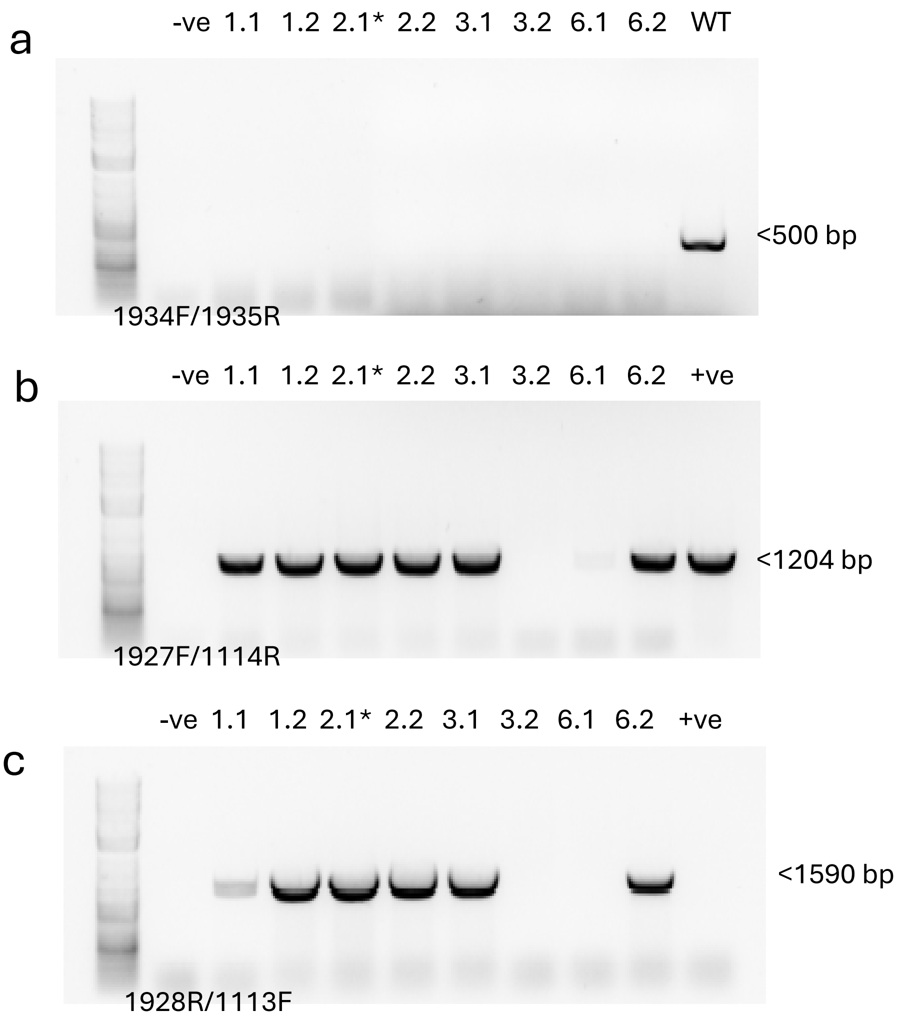

Figure S26. PCR validation of *Fp*133ΔFPOAC2_13302 (MFS transporter) deletion transformants. Single spored clone 2.1 was submitted for metabolomics profiling. **a)** Primer set 1: 1934F/1935R amplifies 500bp region within the gene. Present in WT, but absent in all KO clones. **b)** Primer set 2: Testing for Hygromycin resistance gene 5' region in correct location. Clone 3.2 and 6.1 not showing expected size. Positive control used was *Fp133ΔFPOAC2_13302 clone 1* (not single spored). **c)** Primer set 3: Testing for Hygromycin resistance gene 3' region in correct location. Clone 3.2 and 6.1 do not show expected size, and clone 1.1 has very faint band only (not visible in positive control). Positive control used was *Fp133ΔFPOAC2_13302 clone 1* (not single spored).

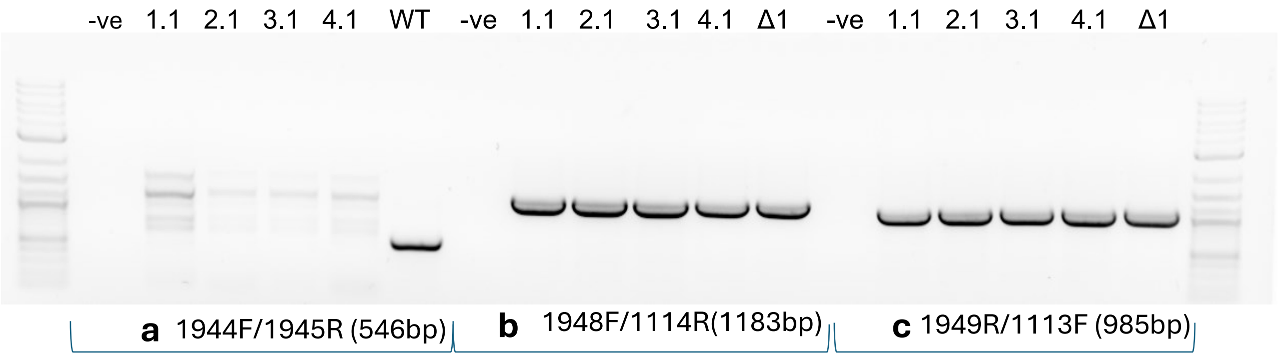

Figure S27. **a)** PCR validation of *Fp*133ΔFPOAC2_13303 (FAD dependent oxidoreductase) deletion transformants. Single spored clone 2.1 was submitted for metabolomics profiling. Primer set 1: Hygromycin resistance gene absent in all four clones tested and present in WT **. b)** Primer set 2: Test for Hygromycin resistance gene 5' presence in correct location. Positive control used was ΔFPOAC2_13303_1 (not single spored)**. c)** Primer set 3: Test for Hygromycin resistance gene 3' presence in correct location. Positive control used was ΔFPOAC2_13303_1 (not singly spored)

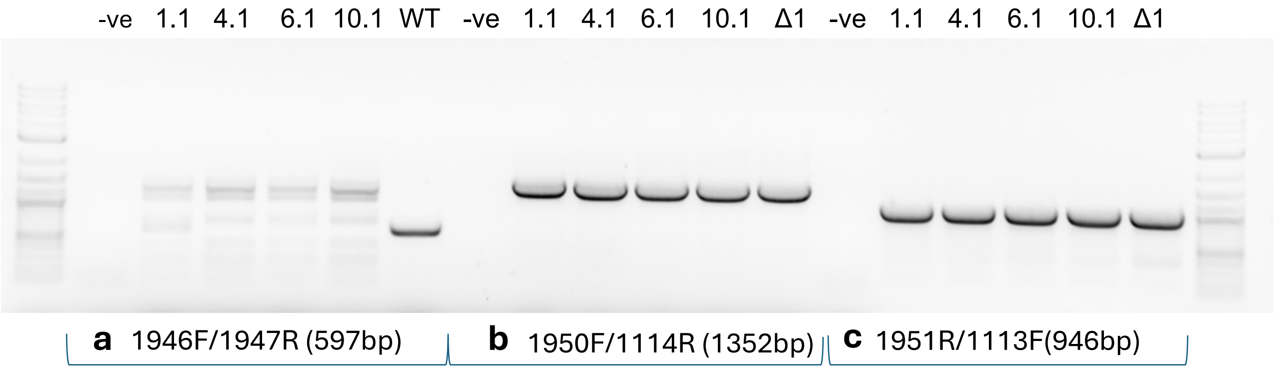

Figure S28. **a)** PCR validation of *Fp*133ΔFPOAC2_13305 (Cysteine synthase) deletion transformants. Single spored clone 1.1 was submitted for metabolomics profiling. Primer set 1: Hygromycin resistance gene absent in all four clones tested and present in WT **. b)** Primer set 2: Test for Hygromycin resistance gene 5' presence in correct location. Positive control used was ΔFPOAC2_13305_1 (not single spored)**. c)** Primer set 3: Test for Hygromycin resistance gene 3' presence in correct location. Positive control used was ΔFPOAC2_13305_1 (not singly spored)

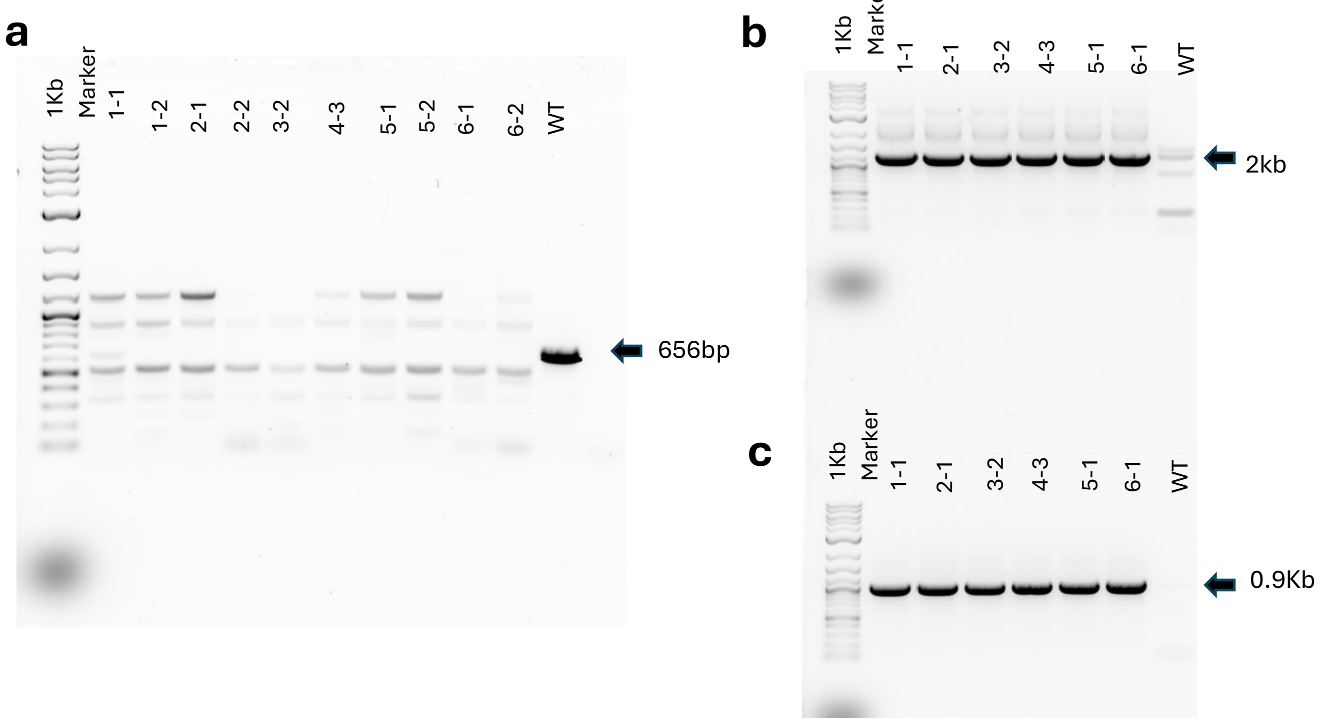

Figure S29. a) PCR validation of *Fp*133ΔFPOAC2_13306 (N-acetyl transferase) deletion transformants. Single spored clone 5-1 was submitted for metabolomics profiling. Primer set 1: Hygromycin resistance gene absent in all four clones tested and present in WT **. b)** Primer set 2: Test for Hygromycin resistance gene 5' presence in correct location. **c)** Primer set 3: Test for Hygromycin resistance gene 3' presence in correct location.

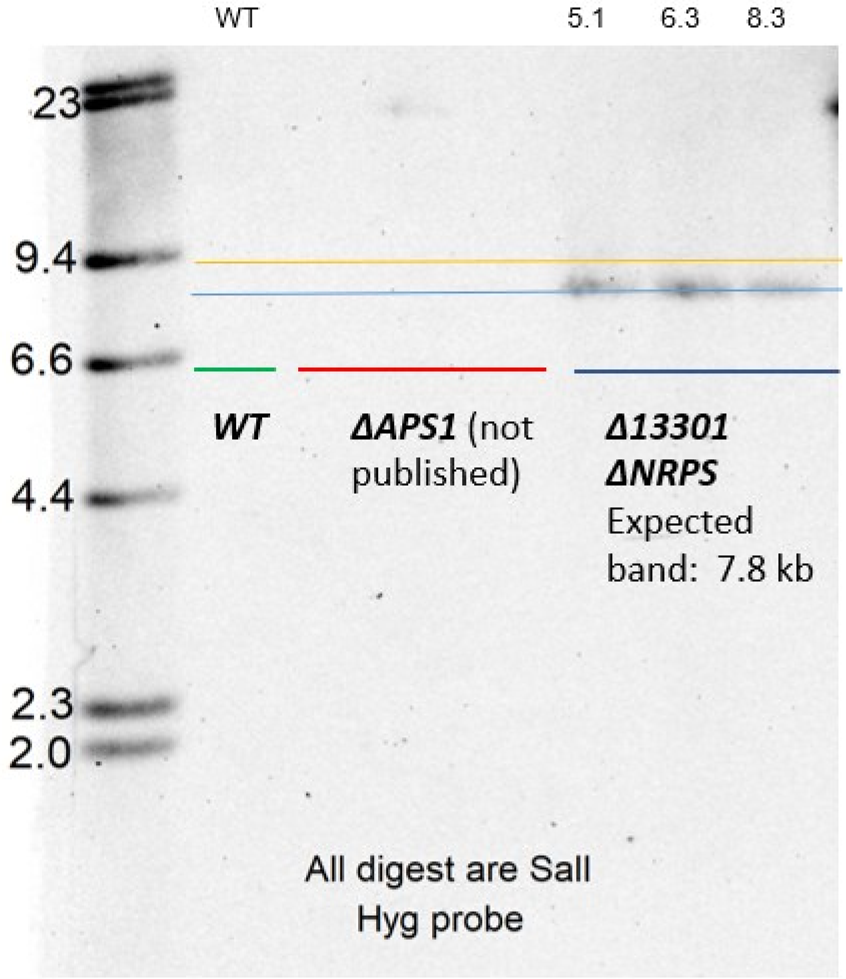

Figure S30. Southern blot gel image for validation of *Fp133Δ13301* (NRPS) gene deletion mutants.

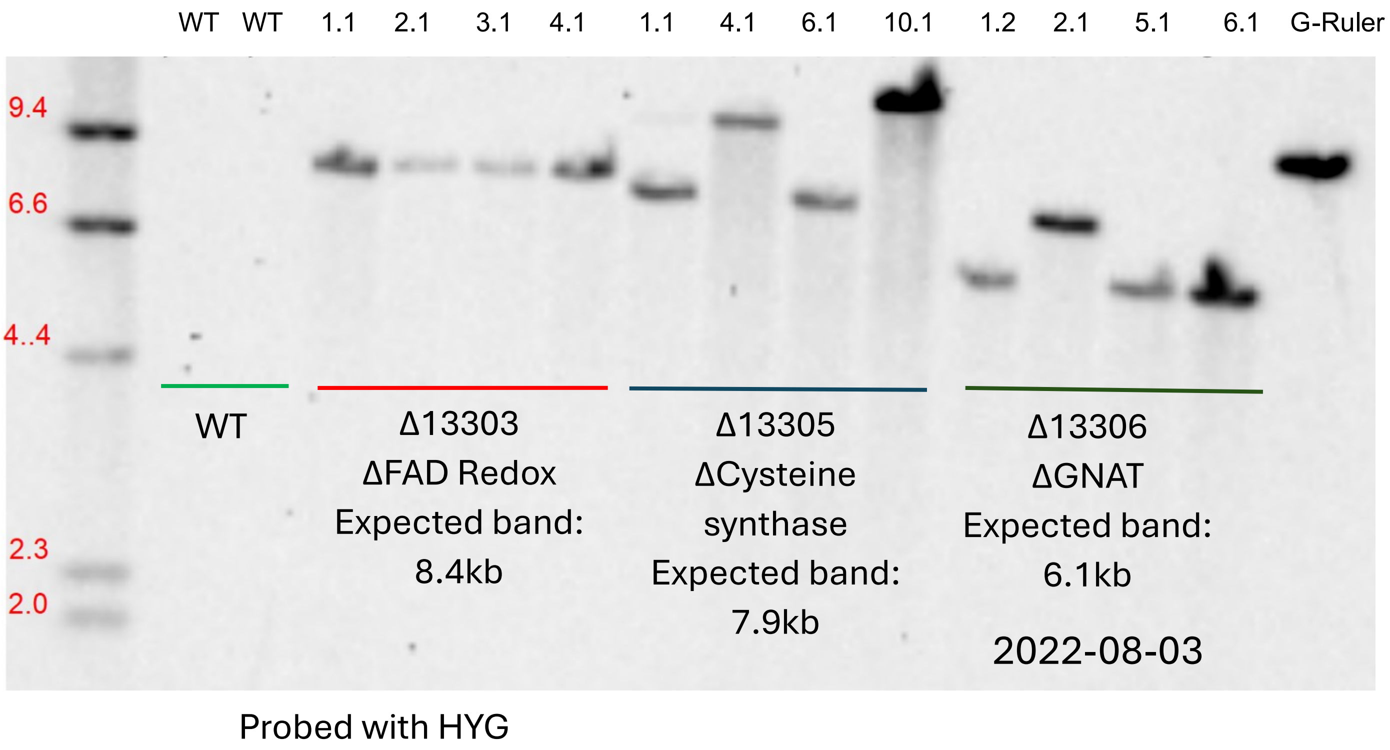

Figure S31. Southern blot gel image for validation of fusadapamide BGC tailoring enzyme gene deletion mutants.

Table S5. Top tBlastn hits comparing Fda1 A-domain protein sequences to all characterized biosynthetic gene clusters from the MIBIG database of natural products. Additionally, stachelhaus specificity codes are included here, as predicted by NRPSPredictor2 as built into the antiSMASH pipeline (v5).

Fda1 NRPS Domain	MIBIG Top tblastn hit (gene ID)	% aa ID	Associated product	Module of hit	Proposed specificity	hit module product	Fda1 stachelhaus code	hit stachelhaus code
A1	Metarhizium anisopliae (MAA_10043)	59.1	Destruxin A	M6	Nme-dap	Nme-alanine	dnwlyggvtk	dvwiyaavik
A2	Metarhizium anisopliae (MAA_10043)	58.7	Destruxin A	M5	Nme-val	Nme-val	dawfyggsfk	dawfyggtfk
A3	Metarhizium anisopliae (MAA_10043)	48.4	Destruxin A	M4	Ile	Ile	dgmfvgaiik	dglfigipvk

Table S6. Fungal NRPS BGCs from the MIBIG database included in the phylogenetic analysis of condensation domains.

Compound name	MIBIG entry	NRPS locus ID	Taxon
AbT1	BGC0000307	aba1	*Aureobasidium pullulans*
acetylaszonalenin	BGC0000293	NFIA_055290	*Neosartorya fischeri*
aculeacin	BGC0001220	ASPACDRAFT_42025	*Aspergillus japonicus*
apicidin	BGC0000304	aps1	*Fusarium incarnatum*
aspercryptin	BGC0001515	ANIA_07884	*Aspergillus nidulans FGSC A4*
aspergillicin	BGC0002160	F9C07_13168	*Aspergillus flavus*
beauvericin	BGC0000313	Beas	*Beauveria bassiana*
beauveriolide	BGC0002259	CCM_01285	*Cordyceps militaris CM01*
BII-rafflesfungin	BG00001966	QCC62999.1	*Phoma sp.*
chrysogine	BGC0001545	FGSG_11395	*Fusarium graminearum*
Cyclo- D-Phe-L-Phe-D-Val-L-Val	BGC0000357	PCH_Pc16g04690	*Penicillium rubens Wisconsin 54-1255*
cyclochlorotine	BGC0001402	PISL3812_02619	*Talaromyces islandicus*
cyclosporin	BGC0000334	simA	*Tolypocladium inflatum NRRL 8044*
destruxin	BGC0000337	MAA_10043	*Metarhizium robertsii ARSEF 23*
emericellamide	BGC0001290	ANIA_02545	*Aspergillus nidulans FGSC A4*
ergotamine	BGC0002232	CCE30225.1	*Claviceps purpurea 20.1*
fumiquinazoline	BGC0000355	AFUA_6G12080	*Aspergillus fumigatus Af293*
fusadapamide	NA	FPOAC2_13301	*Fusarium poae*
fusahexin	NA	FPOAC2_02576	*Fusarium poae*
fusaoctaxin	BGC0002172	FGSG_13878	*Fusarium graminearum PH-1*
HC-Toxin	BGC0001166	hts1	*Alternaria jesenskae*
KK-1	BGC0001636	TRAF135001	*Curvularia clavata*
leucinostatin	BGC0001358	VFPBJ_02539	*Purpureocillium lilacinum*
N-acetyltryptophan	BGC0001679	ANIA_10576	*Aspergillus nidulans FGSC A4*
notoamide	BGC0000818	notE	*Aspergillus versicolor*
okaramine	BGC0001717	okaA	*Penicillium simplicissimum*
oxepinamide	BGC0002208	HK57_00065	*Aspergillus ustus*
penicillin	BGC0000404	pcbAB	*Penicillium chrysogenum*
psychrophilin	BGC0002617	AMQ36132.1	*Penicillium sp. YT-2016*
sansalvamide	BGC0001768	NECHADRAFT_106280	*[Nectria] haematococca mpVI 77-13-4*
serinocyclin	BGC0001240	X797_010654	*Metarhizium robertsii*
tentoxin	NA	AA0116_g8247	*Alternaria tennuisima* 1166
tryptoquialanine	BGC0001142	ADY16697.1	*Penicillium aethiopicum*
W-493	BGC0002188	FPSE_09183	*Fusarium pseudograminearum CS3096*

Table S7. Top BLASTp hits comparing Fda3 and Fda4 predicted protein sequences to the NCBI genbank protein database.

Taxon	Sequence ID	Fda3 homolog protein ID	FDA4 homolog protein ID	Fda3 aa % ID match	Fda4 aa % ID match
Neopestalotiopsis sp. 37M*	SWKT01000031.1	KAF3014063.1	KAF3014062.1	56	70
Coleophoma cylindrospora BP6252	PDLM01000017.1	RDW58722.1	RDW58723.1	42	50
Cudoniella acicularis DSM 108380	JAAMPI010000017.1	KAF4637613.1	KAF4637610.1	49	55
Clonostachys rosea 192-96	CABFNS010000705.1	VUC23399.1	VUC23400.1	38	49
Phaeomoniella chlamydospora UCRPC4	LCWF01000168.1	KKY16047.1	KKY16046.1	42	36
Aspergillus burnettii FRR 5400**	SPNV01000223.1	KAF5858054.1	KAF5858055.1, KAF5858056.1	37	N/A (fragmented)

* The *Neopestalotiopsis sp. 37M* BGC has a Fda5 homolog, KAF3014060.1, with 32% aa ID match.

** The *Aspergillus burnettii* FRR 5400 BGC has a Fda5 homolog, KAF5858057.1 , with 56% aa ID match.

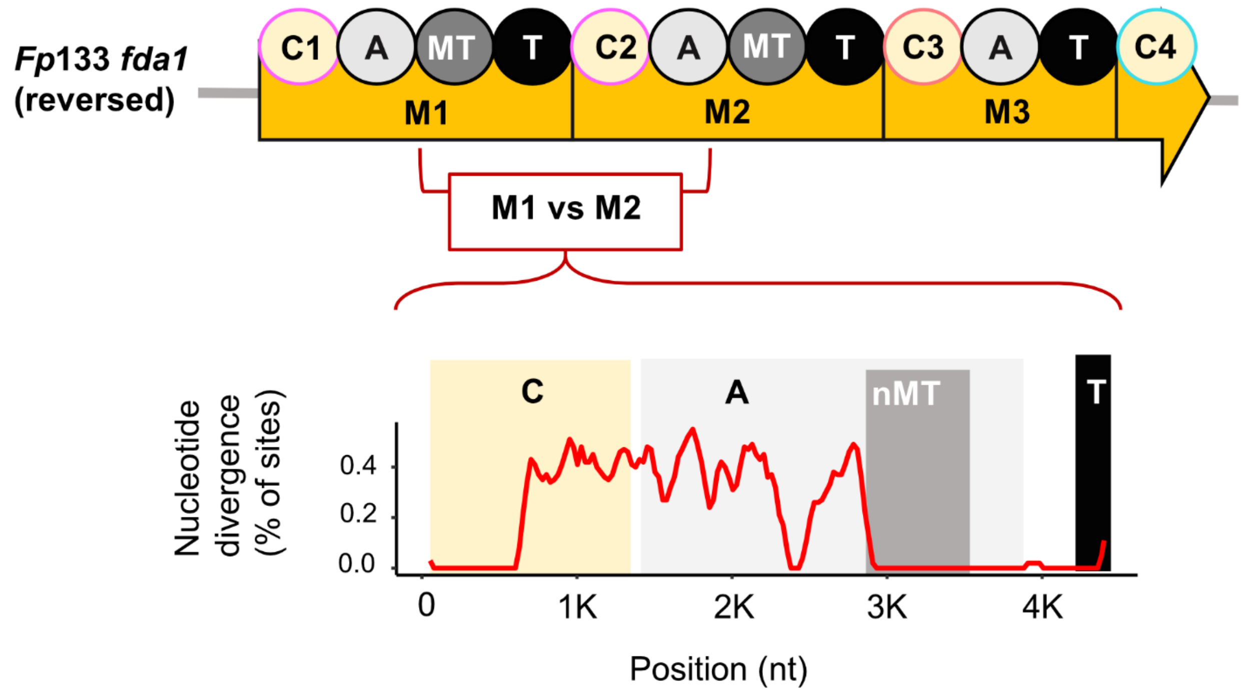

Figure S32. Linegraph comparison of *fda1* nucleotide diversity between modules 1 and 2. Flat regions indicate areas where the two modules are identical.

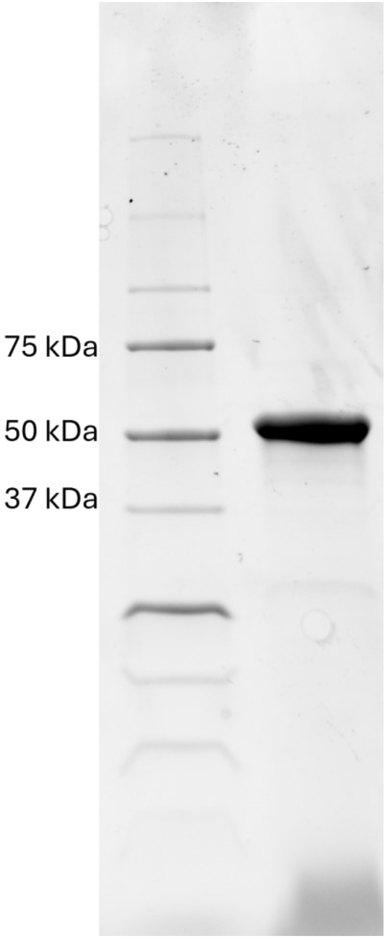

Figure S33. SDS-PAGE of recombinant purified Fda4. Bio-Rad unstained protein ladder #1610363 was loaded to facilitate the estimation of the protein's molecular weight. The molecular weight of Fda4 is predicted to be 54 kDa.

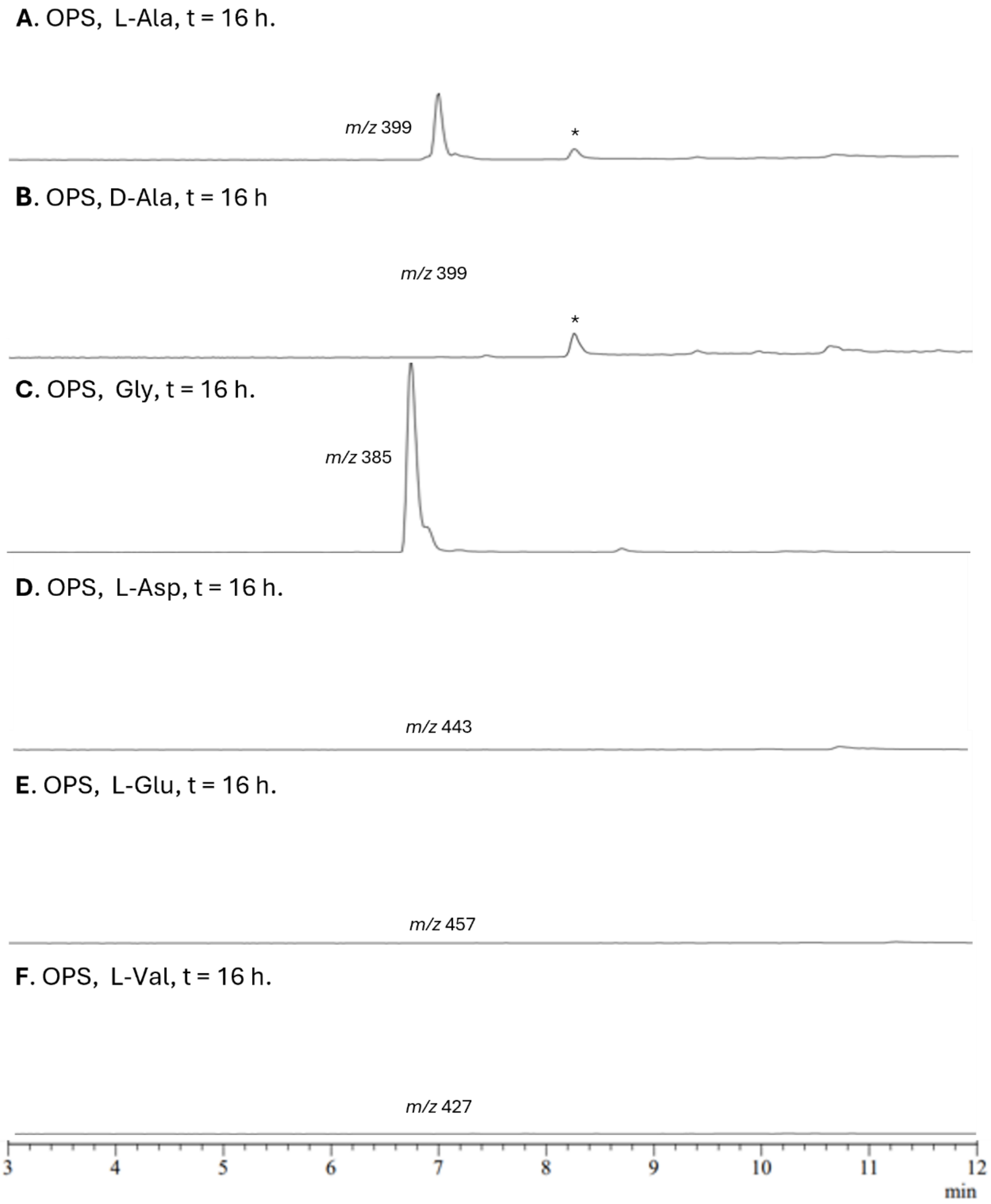

Figure S34. Recombinant purified Fda4 uses L-Ala and Gly as nitrogen sources in a condensation with OPS to produce $[M+H]^+$ *m/z* 399 and $[M+H]^+$ *m/z* 385, respectively. **A**. OPS (10 mM), L-Ala (10 mM),16h; **B**. OPS (10 mM), D-Ala (10 mM), 16h; **C.** OPS (10 mM), Gly (10 mM), 16 h; **D**. OPS (10 mM), L-Asp (10 mM), 16h; **E**. OPS (10 mM), L-Glu (10 mM), 16h. **F**. OPS (10 mM) with L-Val (10 mM), 16 h. DTT (5 mM) PLP (100 μM), Fda4 (10 μM), 100 mM KCl, 50 mM pH 7.4 phosphate buffer. Reactions were derivatized with Fmoc-Cl and run on LCMS. Ion extractions for Product $[M+H]^+$ *m/z* are shown. *Not an assay specific peak.

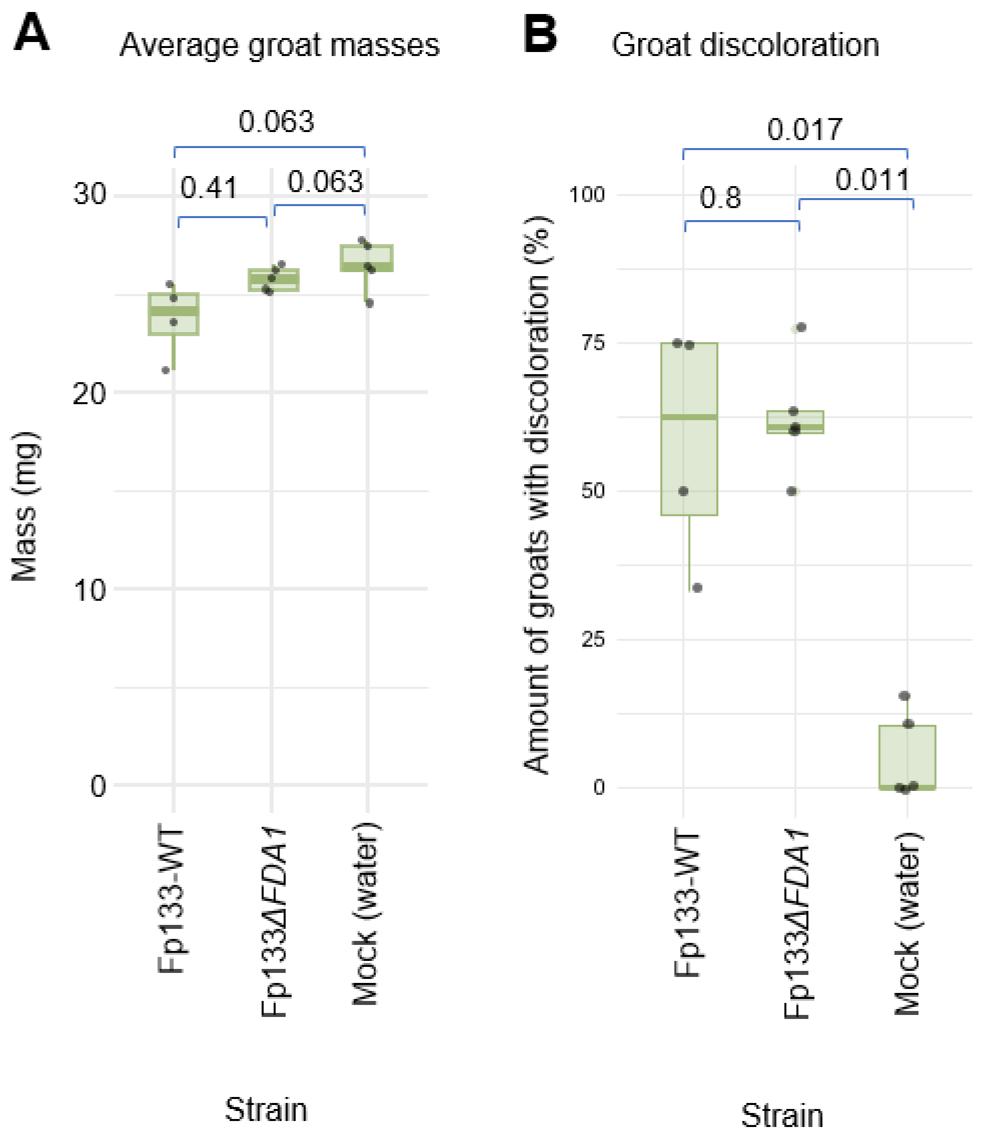

Figure S35. **A.** Average groat masses from Amaze line oats inoculated with spores from Fp133 WT, Fp133ΔFDA1 and Mock (water only) control. Each point represents the average of between 15-26 groat masses from manually inoculated spikelets (each pot, containing 3 plants, equals one sample). P-values shown above brackets represent pairwise non-parametric Wilcoxon tests. The p-value from a Kruskal-Wallis test was 0.061. **B.** Percentage of groats which showed any signs of discoloration, from the samples included in plot A. Discoloration was defined as brown to black shading on the groat coat, and was measured by the experimenter (results are preliminary and should not be interpreted as conclusive). P-values represent pairwise non-parametric Wilcoxon tests. The p-value from a Kruskal-Wallis test was 0.01.

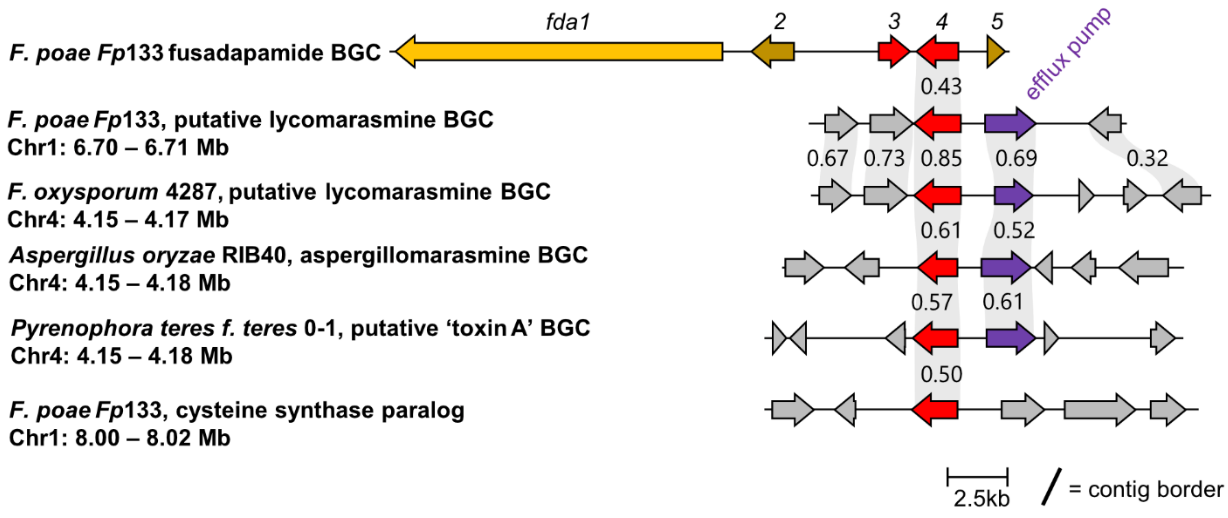

Figure S36. Synteny analysis comparing the *F. poae Fp*133 fusadapamide BGC to putative lycomarasmine clusters from *F. oxysporum* and *Fp*133, the putative 'toxin A' cluster from *Pyrenophora teres*, the characterized aspergillomarasmine A biosynthetic cluster in *Aspergillus oryzae*, and a third *fda4* paralog in *Fp*133. Grey genes are not predicted to play a role in secondary metabolite biosynthesis. Numbers overlaid on synteny blocks represent amino acid identity % match between predicted peptide sequences.

Table S8. RNAseq-derived expression (in transcripts per million) of Fp133 gene models predicted to contain N-acetyltransferase domain HMMs.

Locus_ID	Gene_ID	Chrom	MMK2_d2	MMK2_d6	YES_d2	YES_d6
FPOAC2_00110		Chr1	1.8	6.7	3.6	5.7
FPOAC2_00205		Chr1	6.6	3.1	7.2	4.0
FPOAC2_00333	GCN5	Chr1	17.5	31.4	20.4	30.8
FPOAC2_02101	naa20	Chr1	34.3	34.6	41.3	23.9
FPOAC2_02197		Chr1	5.7	9.6	5.8	11.0
FPOAC2_02200		Chr1	16.5	8.9	20.9	11.6
FPOAC2_02271	ELP3	Chr1	19.9	27.5	23.1	23.0
FPOAC2_03127		Chr1	9.0	15.5	32.7	27.4
FPOAC2_03512	ARD1	Chr1	58.3	39.2	57.3	37.3
FPOAC2_03927		Chr1	6.6	7.5	7.4	10.5
FPOAC2_04142		Chr2	2.2	16.8	471.9	2360.8
FPOAC2_04531		Chr2	7.5	2.6	9.3	4.9
FPOAC2_05004		Chr2	19.7	40.8	32.1	48.0
FPOAC2_05068		Chr2	35.1	48.4	35.9	49.2
FPOAC2_05210	GNA1	Chr2	136.7	100.0	152.1	132.3
FPOAC2_05324		Chr2	2.7	7.5	2.2	5.7
FPOAC2_05367		Chr2	0.1	0.1	0.0	1.0
FPOAC2_05663		Chr2	0.5	2.5	2.2	10.2
FPOAC2_05718		Chr2	0.9	2.6	0.9	4.0
FPOAC2_05984		Chr2	3.8	10.0	3.8	11.3
FPOAC2_06070		Chr2	5.5	7.1	9.0	13.5
FPOAC2_06202		Chr2	6.8	7.7	6.6	8.8
FPOAC2_06654		Chr2	1.4	22.8	4.1	25.9
FPOAC2_06804	ats1_1	Chr2	23.5	19.7	28.0	16.2
FPOAC2_06842	ESA1	Chr2	8.9	18.0	10.0	14.4
FPOAC2_07795		Chr3	3.5	33.9	13.3	67.6
FPOAC2_08557		Chr3	13.7	55.7	29.2	139.7
FPOAC2_08839		Chr3	21.1	8.8	9.7	8.1
FPOAC2_08921		Chr3	11.5	18.8	10.0	21.8
FPOAC2_09088		Chr3	127.2	85.3	104.5	94.6
FPOAC2_09103		Chr3	2.4	3.1	3.5	4.2
FPOAC2_09240	SPT10	Chr3	5.1	8.9	5.7	9.4
FPOAC2_09264		Chr3	1.5	3.3	1.7	3.9
FPOAC2_09314	naa30	Chr3	63.3	127.0	81.2	142.8
FPOAC2_10084		Chr3	7.3	8.3	4.9	4.6
FPOAC2_10098	ats1_2	Chr3	0.8	9.1	0.9	6.2
FPOAC2_10600		Chr4	2.6	2.7	2.5	2.6
FPOAC2_10640		Chr4	0.6	1.5	6.1	1.9
FPOAC2_11234		Chr4	5.3	9.6	4.5	9.3

FPOAC2_11689		Chr4	0.4	0.7	0.3	0.3
FPOAC2_11704	HPA3	Chr4	4.9	17.3	7.1	16.6
FPOAC2_11962		Chr4	9.6	24.1	20.7	33.8
FPOAC2_11996		Chr4	12.1	105.1	34.3	73.9
FPOAC2_12249		Chr4	4.3	18.1	3.7	20.1
FPOAC2_12475		Chr4	2.4	2.5	2.5	2.0
FPOAC2_12726		Chr4	39.2	23.8	11.5	34.9
FPOAC2_12930		Chr4	39.0	82.9	50.7	68.8
FPOAC2_13023		Chr4	274.0	40.1	140.2	51.5
FPOAC2_13306	fda5	Chr5	133.0	111.9	137.2	274.0
